# Data source effects on the perceived distribution of ant diversity in the Espinhaço Mountain Range, Brazil

**DOI:** 10.1038/s44185-026-00135-8

**Published:** 2026-05-15

**Authors:** Davi Vilaça, Inácio Gomes, Ricardo Meireles, Ricardo Solar, Frederico Neves

**Affiliations:** Departamento de Genética, Ecologia e Evolução, Universidade Federal de Minas Gerais, Belo Horizonte, Brazil

**Keywords:** Ecology, Ecology, Environmental sciences

## Abstract

We investigated the influence of the data source on taxonomic composition analyses and species distribution model outputs for ants in the Espinhaço Range. Our objectives were to evaluate the taxonomic composition reported on a global repository (GBIF plus GABI) and regional compilations (PELD-CRSC plus Atlantic Ants), to compare stacked species distribution models (SSDMs) built from each source, and to examine climate–richness relationships using a combined model. We compiled occurrences, cleaned the data and then fitted SSDMs with five algorithms and using five bioclimatic variables selected after variance-inflation screening. We also examined richness maps and climate–richness relationships using Spearman correlations. Subfamily and species compositions diverged across repositories. The global dataset contained only ~10% of the records of the regional dataset, resulting in much lower richness magnitudes. Despite this, the models from both datasets were highly correlated. All models consistently indicated higher species richness in the southern Espinhaço, which was strongly associated with lower isothermality and higher precipitation. Despite the importance of global repositories, regional ones are essential for accurate estimates of ant composition and richness. Thus, integrating datasets improved coverage and highlighted significant knowledge gaps in central and northern Espinhaço.

## Introduction

Biodiversity is declining faster than we can measure it, yet it still underpins essential ecosystem processes and services for human well-being^1–3^. More common threats such as climate change and habitat loss, are driving sharp declines, demanding effective conservation strategies^4,5^. To keep pace, ecologists have turned to massive species-occurrence repositories, which have become key sources of biological information supporting macroecological distribution analyses, gap assessments, invasion risk forecasts, conservation planning, and climate-impact studies^6–8^. Species-occurrence databases are often assumed to adequately represent biodiversity, despite well-documented spatial and taxonomic gaps that emphasize the importance of careful data-source selection^9^.

Major biodiversity data aggregators include speciesLink, iDigBio, VertNet, Canadensys, and the Global Biodiversity Information Facility (GBIF), which hosts ~2.69 billion records and underpins more than 5000 publications (https://www.gbif.org/). Despite well-known concerns about taxonomic accuracy, citation quality, and data vetting, GBIF remains the most widely used biodiversity repository and a central data-mobilization initiative^10–12^. Meanwhile, many rich regional datasets remain underused due to limited dissemination, disincentives to open data, fears of misuse, concerns that open data sharing may reduce academic competitiveness, including loss of publication priority and control over future analyses and weak institutional rewards^13,14^. The differences among these biodiversity repositories extend beyond record volume and accessibility and can strongly influence inferred patterns of species richness, taxonomic composition, and environmental associations^15,16^. Variability in sampling effort, taxonomic resolution, spatial bias, and metadata completeness means that the same *taxa* may be represented in fundamentally different ways across databases, even within the same geographic region^16^. As a result, ecological conclusions derived from a single data source may reflect repository-specific biases rather than underlying biological patterns^17,18^. Explicitly evaluating how alternative data sources shape model outcomes is therefore a necessary step toward more robust biodiversity inference.

Against this backdrop of cross-repository variability, researchers increasingly rely on large species occurrence databases to study distributional patterns^11,13,15^, and SDMs now occupy a central role in it^19^. Typical workflows rely on compiling occurrences, fitting models, validating their performance (e.g., AUC, TSS, Kappa), and interpret outputs^20,21^. The reliability of each step depends on data completeness, curation, and spatial coverage, which often vary across repositories and can propagate biases into models^15,16,18^. Given that SDMs are shaped by the strengths and limitations of their underlying data, meaningful assessment of data-source effects requires repositories that differ substantially in coverage and curation but represent the same biota.

The Espinhaço Range in Minas Gerais and Bahia, Brazil, offers such a natural experiment. This ecotonal and species-rich montane system spans the Cerrado, Atlantic Forest, and Caatinga biomes, as well as the high-altitude, rocky grassland ecosystem known as *campos rupestres*^22^. These diverse features underpin the strong environmental gradients of the region, alongside its distinctive geological and evolutionary history, which together shape biodiversity and ecological processes in the Espinhaço^23,24^. Within this diversity and landscape, ants provide an especially suitable focal group because they are important ecosystem engineers, exhibit high local diversity, and respond sensitively to climatic and habitat variations^25,26^. For example, the *campo rupestre* portion of the Espinhaço has been studied for the distribution of Hymenoptera, including ants, revealing a complex relationship between species distributions and environmental variables, with no significant effect of latitude on the gradient of ant richness^27^. Moreover, local ant diversity is high, but still poorly captured by global aggregators, such as GBIF, despite the existence of curated regional datasets, making this region an ideal setting to compare repositories^28^. Global platforms also exhibit observer and taxonomic biases that emphasize plants and vertebrates^29^, whereas curated regional surveys in the Espinhaço provide taxonomically robust ant data^27,28,30^.

This contrast, sparse global coverage versus rich regional compilations, offers an opportunity to test how data-source choice shapes taxonomic composition and SDM-derived richness. This metric is estimated by stacking individual SDMs, which allows richness to be predicted continuously across space rather than inferred from uneven occurrence records alone^31^. This approach has been widely applied in macroecological research to infer broad-scale biodiversity patterns, identify biodiversity hotspots, and support conservation planning^8^. Importantly, SDM-based richness estimates enable direct comparisons of how different data sources translate into predicted biodiversity patterns, making this metric particularly well-suited for evaluating the effects of data provenance and repository choice on inferred richness gradients.

Therefore, in this study, we assessed how data-source choice influences ant biogeography in the Espinhaço Range. Specifically, we (i) evaluate regional and global repositories quantitatively and qualitatively to identify key gaps and biases; (ii) compare stacked SDMs derived from each source; and (iii) use a combined model to examine climate–richness relationships. We hypothesize that models from global and regional sources will be highly correlated despite differences in taxonomic representativeness and record counts because both are exposed to similar sampling biases (e.g., geographically clustered effort)^15,16^. We further hypothesize that the southern Espinhaço will show higher richness linked to higher precipitation and stronger Atlantic Forest influence, consistent with the idea that several Neotropical ant lineages diversified in forested regions^32,33^.

## Results

### Taxonomic data

**Fig. 1:**
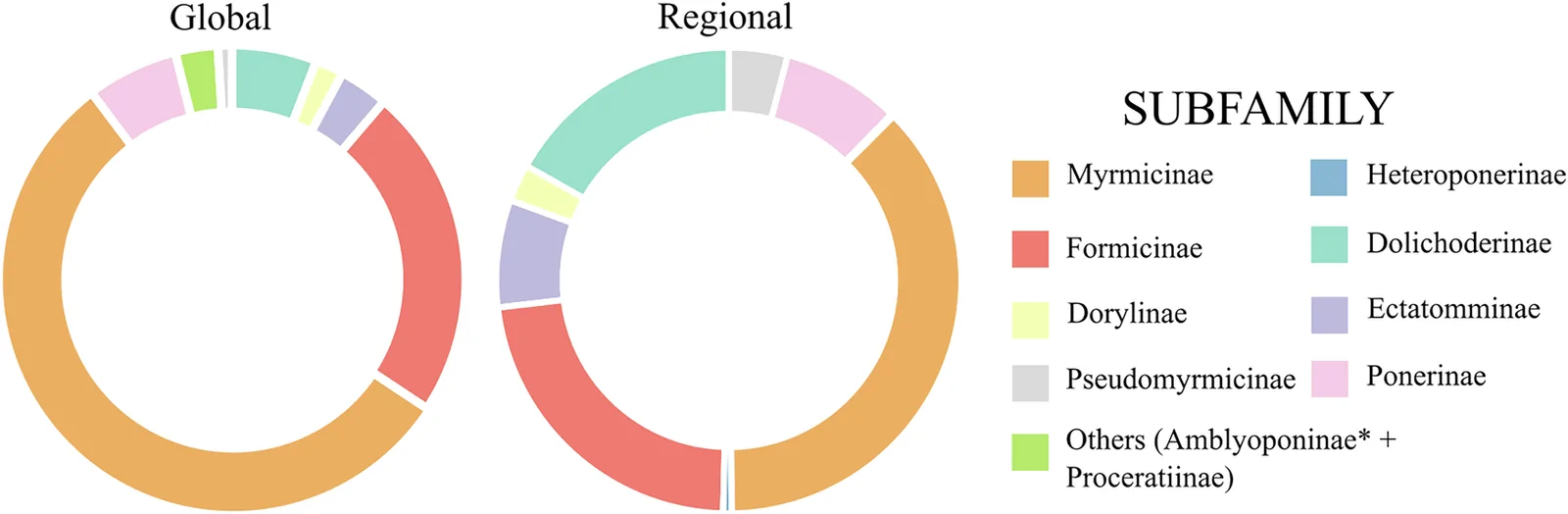
Proportional representation of ant subfamilies in the Espinhaço Range based on global and regional datasets. An asterisk (*) indicates that the subfamily *Amblyoponinae* is absent from the global dataset.

**Fig. 2:**
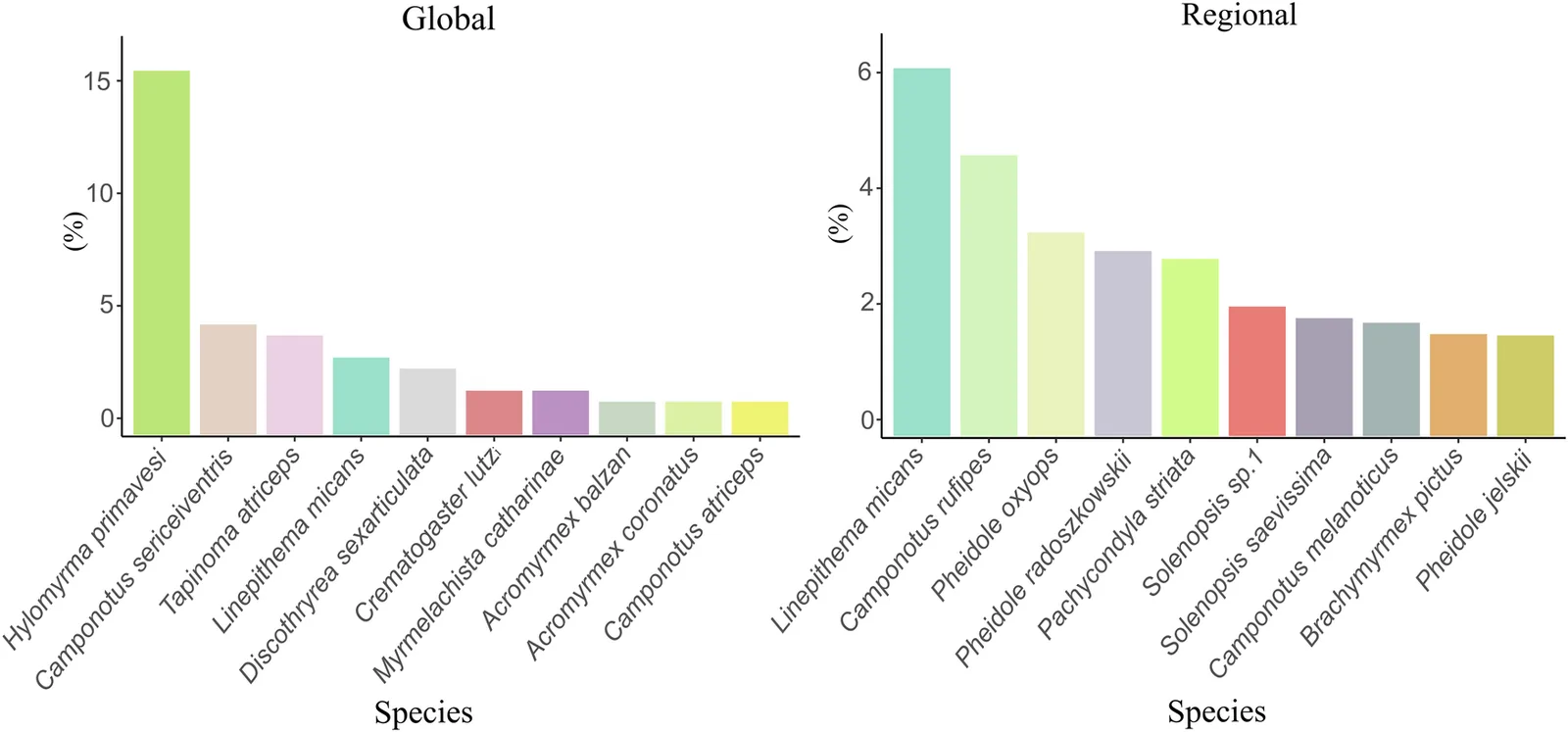
Percentage distribution of the ten most frequently recorded ant species in the Espinhaço Range before the cleaning protocol, based on global (*N* = 204) and regional (*N* = 20,991) datasets. *Linepithema micans* was the only species common to the top ten in both datasets.

Within the boundaries of the Espinhaço Range, the global dataset comprised 9 subfamilies and 101 species, whereas the regional dataset included 10 subfamilies and 1177 morphospecies. After data cleaning restricted to records from the Espinhaço Range, removing morphospecies and records with taxonomic uncertainty (e.g., *indet*., *aff*., *cf*.), species richness in the regional dataset was reduced to 226 species across the same 10 subfamilies, representing approximately 20% of the originally recorded regional species. The global dataset remained unchanged, retaining 101 species distributed across 9 subfamilies. A total of 76 species were shared between the global and regional datasets. Subfamily composition was broadly similar across repositories (Fig. 1). Myrmicinae, Formicinae, Dolichoderinae, and Ponerinae were the most represented subfamilies, consistent with prior accounts for the Espinhaço ant fauna^28^. The global set, however, shows a higher proportion of Heteroponerinae and lacks Amblyoponinae (which was present in the regional data). Among the ten most recorded species in each repository (Fig. 2), the global dataset was dominated by *Hylomyrma primavesi* Ulysséa, 2021, *Camponotus sericeiventris (*Guérin-Méneville, *1838)* and *Tapinoma atriceps* Emery, 1888, whereas the regional dataset was dominated by *Linepithema micans* (Forel, 1908), *Camponotus rufipes* (Fabricius, 1775) and *Pheidole oxyops* Forel, 1908. *L. micans* was the only species shared among the top ten lists of all datasets.

### SSDMs

**Fig. 3:**
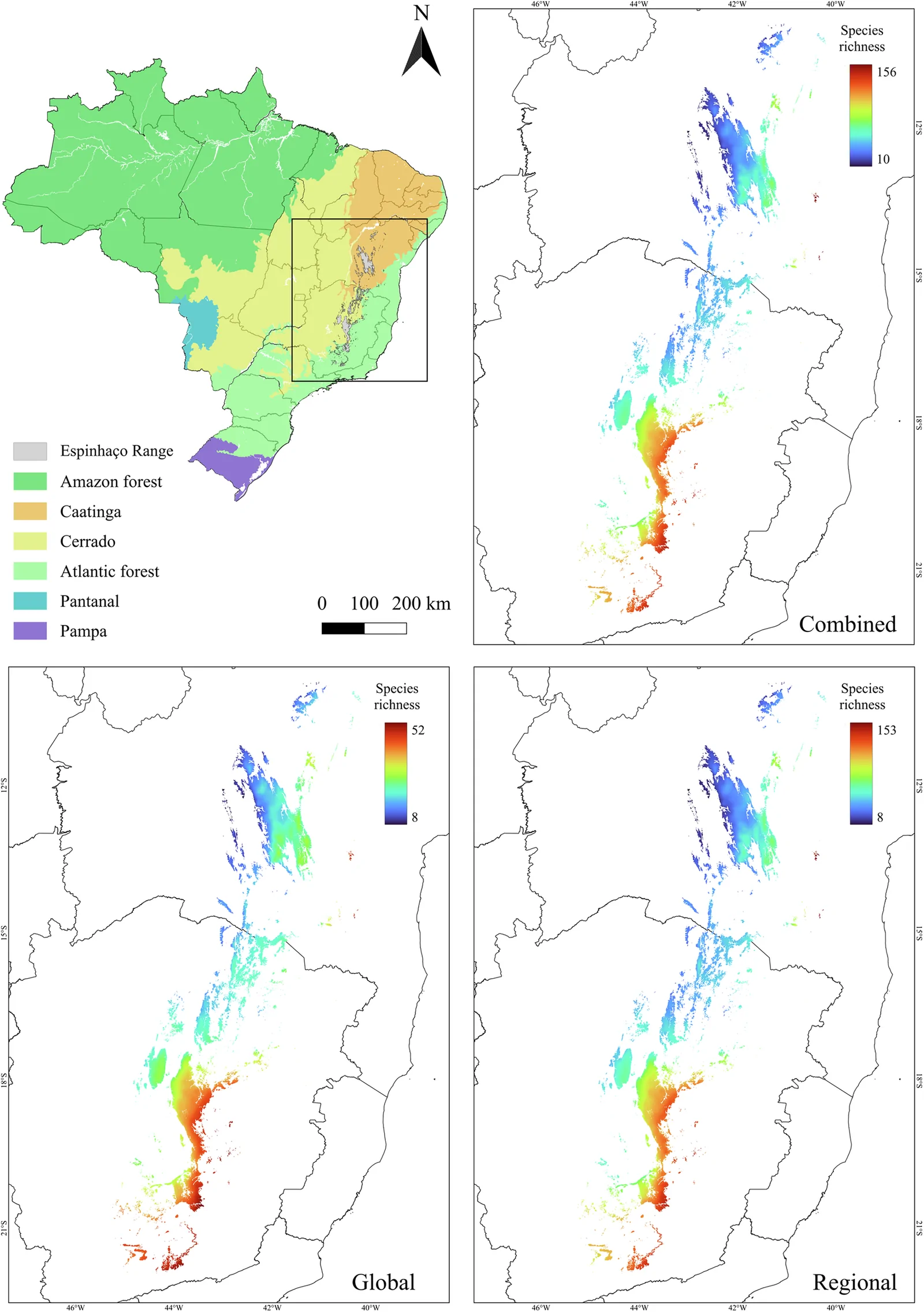
Stacked species distribution model (SSDM) maps of modeled ant species across the Espinhaço Range derived from the global dataset, the regional dataset, and the combined dataset. Brazilian biomes are delineated as follows: yellow = Cerrado; orange = Caatinga; dark green = Atlantic Forest; purple = Pampa; light green = Amazon forest and blue = Pantanal. Species richness is mapped from dark blue (low) to red (high).

**Fig. 4:**
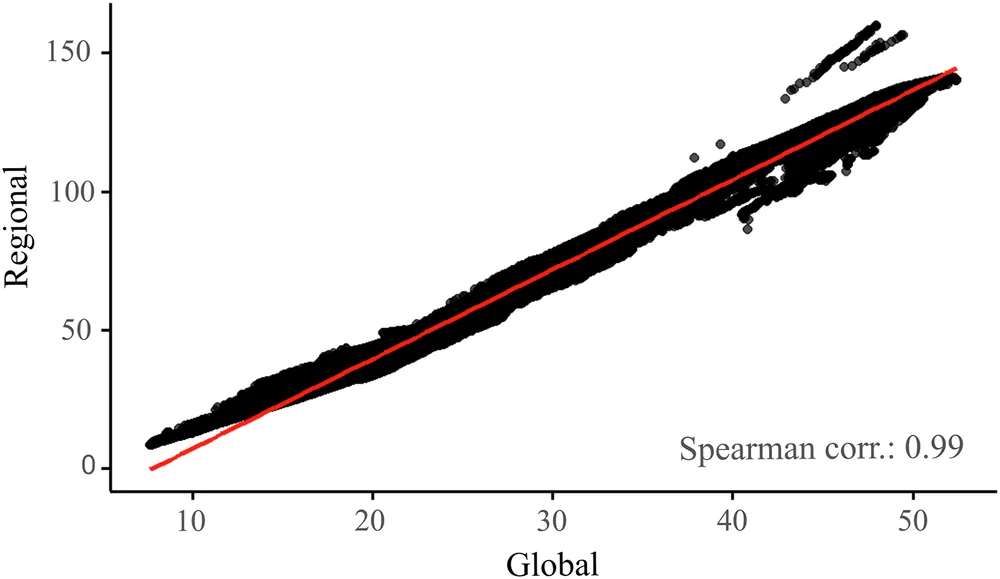
Pixel-wise comparison of modeled species richness between regional and global datasets.

For SDM analyses, we compiled 24,319 occurrence records from the Southeastern and Northeastern regions of Brazil, derived from the global (*n* = 2836) and regional (*n* = 21,483) datasets. Following data filtering, 114 species (global), 344 species (regional), and 352 species (combined) were retained for model construction. We fitted SDMs separately for the global and regional repositories and produced an additional combined model (Fig. 3). The modeled richness values differed sharply: the regional model reached 153 species per pixel at its maximum, while the global model did not exceed 52 species. This order-of-magnitude contrast reflects the much smaller number of occurrences registered in the global dataset. Despite this difference, the spatial patterns of predicted richness were highly concordant between sources (Spearman *ρ* = 0.99; Fig. 4), indicating that the sparse global data still captured the main ecological gradients present in the region.

**Fig. 5:**
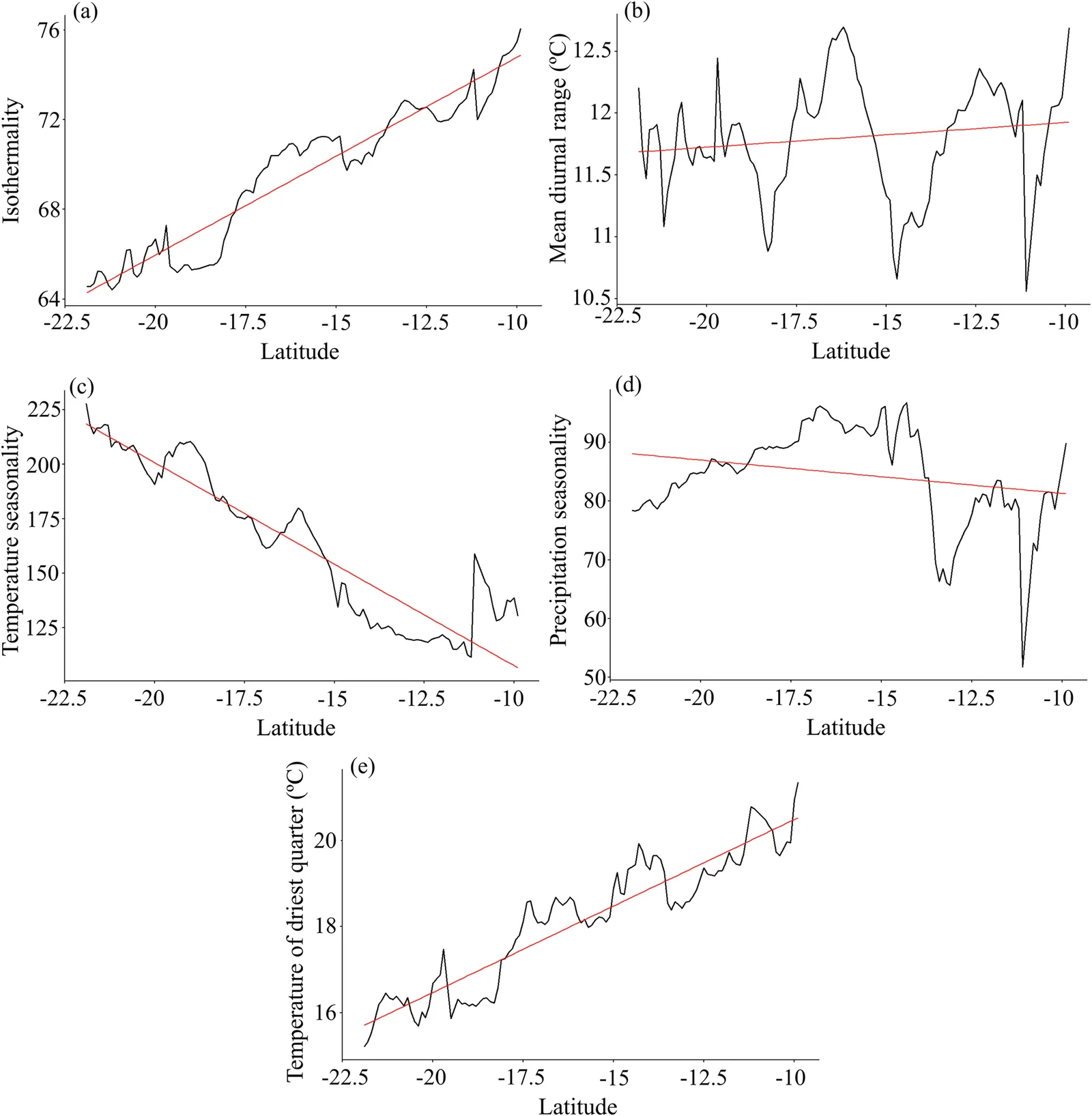
Latitudinal variation of key climatic variables across the Espinhaço Range. Panels show latitudinal trends in **a** isothermality, **b** mean diurnal temperature range, **c** temperature seasonality, **d** precipitation seasonality, and **e** mean temperature of the driest quarter.

Using the combined predicted species richness surface as the response, we examined the relationships between it and environmental predictors through variable-importance estimates and latitudinal patterns (Fig. 5 and Table 1). The jackknife analysis indicated that mean diurnal temperature range and isothermality were the two most influential predictors, contributing 24.7% and 24.0% to model performance, respectively, followed by precipitation seasonality (19.3%), mean temperature of the driest quarter (16.3%), and temperature seasonality (15.6%) (Table 1). Latitudinal analyses revealed marked gradients in climatic conditions across the Espinhaço Range (Fig. 5). Isothermality, which expresses the ratio between mean diurnal temperature range and annual temperature range, increased toward lower latitudes, while temperature seasonality decreased. Together, these patterns indicate a shift toward greater relative daily thermal stability in the southern portion of the Espinhaço Range. Mean diurnal temperature range exhibited relatively limited spatial variation but showed slightly lower values at lower latitudes. Precipitation seasonality displayed a weak latitudinal trend, while the mean temperature of the driest quarter increased consistently toward the north. Together, these patterns indicate that areas of higher predicted species richness coincide with regions characterized by lower isothermality, reduced daily thermal amplitude, and colder conditions during the driest quarter.

**Table 1:** Percent contribution of environmental predictors to the species distribution models

Environmental variables	Percent of contribution (%)
Mean diurnal range (Mean of monthly (max temp−min temp))	24.7
Isothermality	24.0
Precipitation seasonality	19.3
Mean temperature of driest quarter	16.3
Temperature seasonality	15.6

## Discussion

Our comparison revealed a stark imbalance in data availability between sources: the regional repository held roughly 7.5 times more ant records than the global repository. Both sources also showed a south-biased sampling footprint, a pattern noted in previous studies and plausibly linked to the geographic concentration of research institutions and easier access in the south^34,35^. This unequal effort constitutes a Wallacean shortfall in the northern Espinhaço: the sparse records from that region limit representativeness and can introduce bias into models, even with systematic subsampling to mitigate clustering^36^. Targeted surveys in the central and northern sectors, especially along the transition to the Brazilian semiarid, remain a priority to balance coverage and strengthen ecological inferences.

A marked difference between datasets was the prevalence of morphospecies in the regional compilation, in contrast to their near absence in the global dataset. This discrepancy reflects differences in data sources and curation practices: regional datasets often incorporate primary survey data and unpublished records that prioritize local completeness but may include provisional identifications, while global databases typically undergo stricter taxonomic standardization and filtering^11,12,29^. Consequently, although the regional dataset offers finer spatial and ecological resolution, it is more susceptible to taxonomic uncertainty. In contrast, the global dataset provides greater taxonomic consistency but may underrepresent locally rare or undescribed diversity. These trade-offs highlight the importance of dataset-specific curation strategies and reinforce the need for systematic taxonomic validation, particularly for regional-scale ecological analyses^37^.

Differences between repositories were most pronounced at the species level, but relevant contrasts were also evident at the subfamily level, reflecting differences in data provenance, sampling strategies, and metadata availability. For the global dataset, detailed information on sampling methods was largely unavailable, except for human observations, which accounted for approximately 18% of records and were primarily derived from citizen-science platforms (e.g., iNaturalist) (https://www.inaturalist.org). This lack of methodological metadata limits our ability to fully evaluate detection biases in the global compilation. Nevertheless, the global dataset showed a relatively higher proportional representation of subfamilies such as Proceratiinae, particularly the genus *Discothyrea* and Heteroponerinae (e.g., *Acanthoponera*).

In the case of *Discothyrea*, this apparent prominence is likely influenced by targeted sampling and focused taxonomic attention within a comparatively small and sparse dataset, rather than by true ecological dominance. Species of this genus are characteristically cryptic, occur at low colony densities, and nest in concealed microhabitats, making their detection highly dependent on sampling effort and method^38^. In contrast, the regional dataset was accompanied by explicit sampling information and consisted predominantly of standardized pitfall traps or Winkler extractors, which efficiently sample soil and leaf-litter assemblages^39^. Notably, in the global dataset the most frequently recorded species (*Hylomyrma primavesi*) originated from a single collection event^40^, illustrating how sparse sampling and uneven effort can inflate its apparent prevalence. More broadly, the most common species in the global dataset tend to include *Camponotus sericeiventris* and *Acromyrmex* spp., which are generally larger-bodied or highly surface-active ants^41^. In addition, species associated with arboreal or epigaeic foraging strata, such as *Crematogaster*, *Hylomyrma*, and *Tapinoma*. In contrast, the regional dataset is dominated by species characteristic of leaf-litter and soil environments (e.g., *Pheidole* spp., *Solenopsis* spp.)^28,41^, reflecting the use of standardized sampling methods such as pitfall traps. Together, these divergences in taxonomic composition between datasets underscore the role of sampling bias and data sparsity.

The spatial patterns of richness were highly correlated between the global and regional models (*ρ* ≈ 0.99), however, their magnitudes differed by an order of magnitude. The global model’s ceiling of 52 species per pixel, reflects limitations in data availability, producing a classic “garbage in, garbage out” effect, underscoring that regional repositories are indispensable for producing realistic richness values. Global aggregators remain useful for capturing broad-scale gradients, but they underrepresent ants in this region and thus severely under-estimate absolute richness. Integrating the data sources improved geographic coverage and highlighted under-sampled areas, but the reliable signal in our models came primarily from the regional data. Despite these disparities in data quality and richness magnitude, the combined model yielded coherent ecological patterns, revealing strong climatic controls on diversity. However, we note that the calibration extent adopted in this study may not fully capture the complete climatic envelopes of widespread species whose distributions extend beyond Brazil. While the Northeast and Southeast regions encompass substantial environmental heterogeneity^42^, portions of the broader Neotropical niche space remain unrepresented. Future studies could explore species-specific calibration areas or broader Neotropical extents to refine individual model performance. Nevertheless, within the comparative framework of this study, maintaining a consistent spatial domain across datasets was essential to ensure that differences in model outputs reflect data source characteristics rather than differences in spatial extent.

Although sampling density was higher in the southern Espinhaço, warranting a cautious interpretation of climate–richness associations, the observed patterns are consistent with well-established biogeographic gradients for Neotropical ants^32,33^, including the reversed latitudinal pattern in which richness increases toward higher (southern) latitudes rather than toward the equator^33^. In our models, the combined importance of isothermality and mean diurnal temperature range, together accounting for nearly half of the total variable contribution, indicates that short-term thermal stability plays a central role in shaping richness patterns across the Espinhaço Range. Although previous studies have emphasized precipitation and water availability as primary drivers of richness in Neotropical ants, our models attributed relatively greater importance to temperature-related variables. This difference does not contradict established patterns; rather, it reflects that thermal stability and water availability act jointly, capturing complementary aspects of the same underlying climatic constraints. Accordingly, our findings are consistent with the view that species richness in the Espinhaço Range tends to be higher in environments characterized by marked seasonal temperature variation combined with a relatively stable daily thermal regime, a pattern that could reflect favorable energy–water conditions for species persistence^43^.

Our findings partly contrast with previous studies focusing on the *campo rupestre* ecosystem in the Espinhaço Range, which did not detect a latitudinal gradient in species richness^27^. That ecosystem is an OCBIL (old, climatically buffered, infertile landscape) where climate varies more strongly with elevation than with latitude, and long-term environmental stability can obscure latitudinal trends^23^. By analyzing the entire Espinhaço Range and including its diverse habitats across transitions among the Atlantic Forest, Cerrado, and Caatinga, we captured broader moisture and temperature gradients that likely enabled the detection of a latitudinal pattern. This comparison underscores how the spatial extent, habitat scope, and sampling design of a study can strongly influence the observed diversity patterns in environmentally heterogeneous montane systems. Furthermore, the south-to-north richness contrast in our results may also reflect historical biogeography. The southern Espinhaço overlaps the Atlantic Forest, which has been implicated in the origin and diversification of several Neotropical ant lineages^32,33^. Any evolutionary explanation for the current gradient remains speculative without phylogeographic data, and our SDMs cannot resolve historical causation. Still, the present-day pattern is consistent with a scenario in which water-energy dynamics and historical connectivity combined to shape the distribution of diversity.

Finally, our study reinforces recent calls to mobilize and integrate regional datasets for the Espinhaço Range. A substantial portion of ant records from this region remains absent from open-access platforms like GBIF, limiting global accessibility, synthesis, and comparative analyses. Persistent barriers to data sharing, such as limited incentives, fear of data misuse, and lack of curation capacity, continue to impede progress. Practical steps to improve this situation include digitizing and publishing regional occurrence records with rich metadata and voucher specimens, harmonizing taxonomies, standardizing sampling protocols, depositing derivative datasets in open repositories, and conducting new surveys in the under-sampled northern areas. Such actions will help reduce the Wallacean shortfall, improve the realism of SDMs, and provide a stronger empirical basis for conservation planning across the Espinhaço Range.

Our central message is that the choice of data source strongly governs what we can infer about biodiversity in the Espinhaço Range. Regional repositories provided the dense sampling and taxonomic resolution needed for realistic richness estimates and community composition, whereas global aggregators, despite capturing broad spatial gradients, still underrepresented ants and thus underestimated richness estimates. Integrating the two sources improved geographic coverage and exposed under-sampled sectors, but the signals that made our models credible came primarily from the regional data. Using an ensemble of SDMs with low-collinearity climate predictors, we consistently recovered a south richness peak linked to cooler conditions and higher precipitation in the warmest quarter. Because sampling was densest in the south, we interpret these climate–richness associations cautiously: the climate–richness pattern is robust at the modeled scale, but any evolutionary explanation remains tentative and beyond the scope of our data.

Practically, our results point to three priorities. First, mobilize and curate regional data holdings with voucher-backed taxonomy, and publish them through open infrastructures to reduce the Wallacean shortfall. Second, target new surveys in the central and northern Espinhaço to balance sampling effort and improve model realism. Third, report data provenance explicitly and adopt workflow safeguards, such as spatial-thinning matched to raster resolution, rigorous cross-validation, and ensemble modeling, to limit bias. In sum, credible biodiversity inference in complex montane systems depends less on choosing a single “best” algorithm and more on assembling representative data. By demonstrating how regional and global sources differentially shape taxonomic composition and modeled richness, we provide a framework and a set of priorities to guide future research and conservation planning in the Espinhaço Range and other under-sampled regions.

## Methods

### Study area

The Espinhaço Range is a ~1200-km montane belt in eastern Brazil recognized as a UNESCO Biosphere Reserve^44^. Extending across the states of Minas Gerais and Bahia, it intersects three major biomes: Atlantic Forest in the south, Cerrado in the center, and Caatinga in the north, and harbors extensive high-elevation rocky outcrops, known as *campo rupestre*, an ancient ecosystem remarkable for its biodiversity and endemism^15^. We delineated the study area within the Espinhaço domain following the approach described by Silveira et al. ^23^

### Occurrence data

We assembled two occurrence compilations: a global dataset derived from GBIF^45^ and GABI^46^, and a regional dataset obtained from (i) the PELD-CRSC project (Long-Term Ecological Research—*Campos Rupestres Serra do Cipó*, 2012–2018) and (ii) the data paper *ATLANTIC ANTS: a dataset of ants in Atlantic Forests of South America*^30^. To characterize ant diversity, we retained georeferenced records identified to species within Formicidae that fell inside the Espinhaço boundary. These records were used to describe taxonomic composition and cross-repository differences, including summaries of the ten most frequent species and the most represented subfamilies in each dataset. For SDMs, occurrence records were not restricted to the Espinhaço Range, which spans the Southeastern and Northeastern regions of Brazil. Accordingly, data cleaning and model calibration were conducted using records from these broader regions, allowing for a more comprehensive representation of species’ environmental niches^42^. Records prior to 1990 were excluded to align occurrence data with the CHELSA v2.1 climatic baseline (1981–2010)^47^. Taxonomic names were validated based on current ant taxonomic literature, using AntCat (https://www.antcat.org) as a reference platform to verify up-to-date valid species names and synonymies. Morphospecies were removed, as provisional or non-standard labels (e.g., “sp.”, “aff.”) may inconsistently split or merge taxa across sources and bias taxonomic and spatial analyses^37^.

**Fig. 6:**
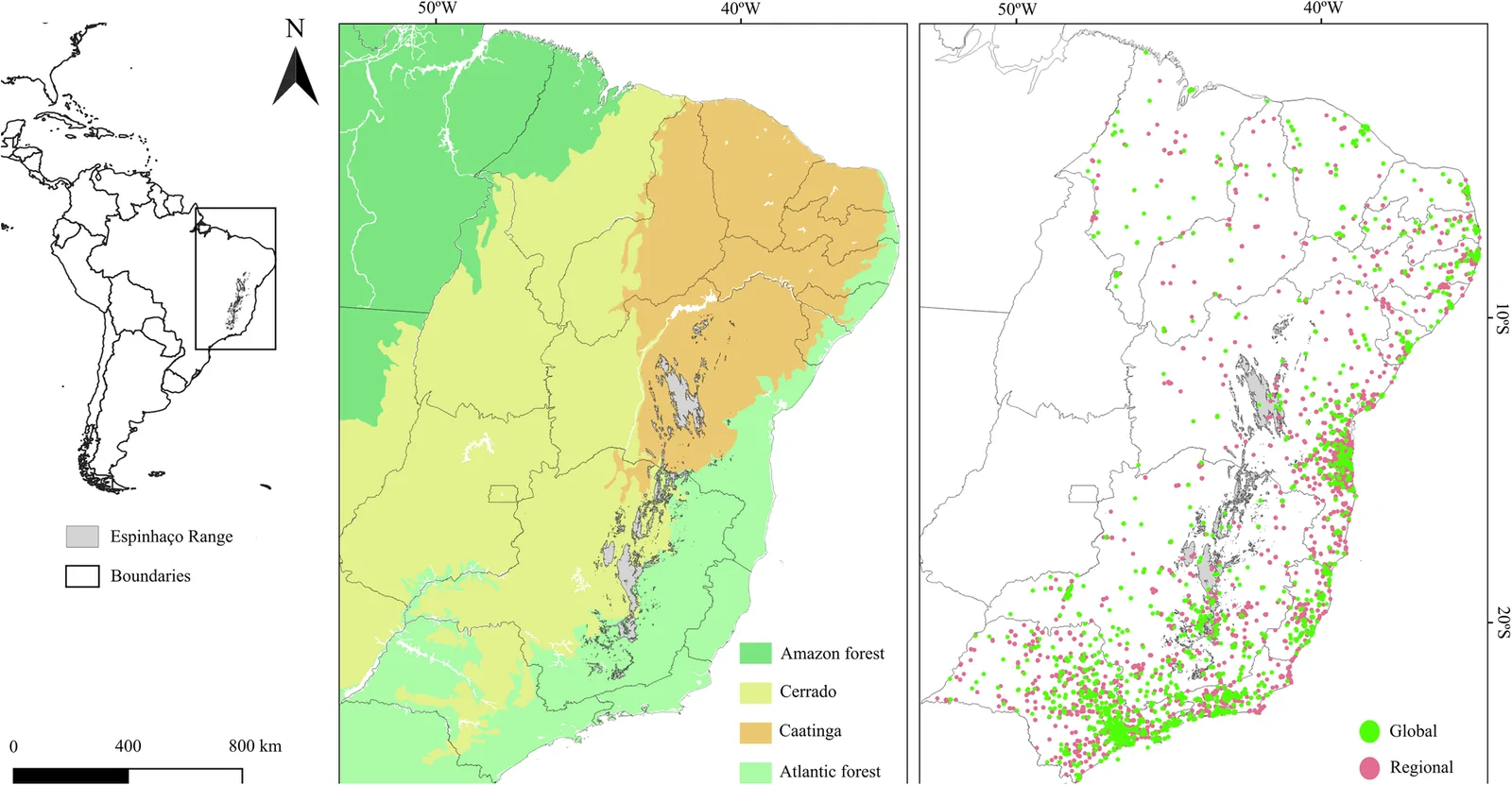
Geographic distribution of ant occurrence records used for species distribution modeling. The inset map shows the American continents and the location of the Espinhaço Range. The left panel depicts the spatial extent of the Espinhaço Range and the major Brazilian biomes surrounding it. The right panel shows the occurrence records used for modeling, concentrated in southeastern and northeastern Brazil, the two regions where the Espinhaço Range is located. Records were compiled from a global dataset (*n* = 2836) and a regional dataset (*n* = 21,483).

Duplicate coordinates were first removed, followed by a 1-km spatial thinning procedure, matching the 30 arc-s raster resolution, applied independently to each species to ensure that no two occurrence records of the same species occupied the same environmental pixel, thereby minimizing local pseudo-replication and reducing within-species sampling bias pattern^48–50^ associated with the pronounced south-biased sampling pattern observed in the datasets because both repositories exhibited a pronounced south-biased sampling (Fig. 6). Following these procedures, SDMs were fitted using three deliberately defined datasets: a global dataset, a regional dataset, and an additional combined dataset constructed as an integrative data source. The cleaned datasets, including the number of occurrence records used for each species in the models, are provided in Supplementary Tables S1 and S2. Species were retained only if they had at least 10 occurrence records, a commonly adopted threshold in SDM studies to ensure a minimum level of model reliability, given the known sensitivity of model performance to small sample sizes^20^.

### Environmental variables and niche modeling

From the 19 CHELSA v2.1 bioclimatic variables for 1981–2010 and elevation, we selected five for modeling: isothermality, mean diurnal temperature range, temperature seasonality, precipitation seasonality, and mean temperature of driest quarter and precipitation of the warmest quarter. We selected these variables by applying a variance inflation factor (VIF) filtered predictor set to limit (VIF < 10), which reduces multicollinearity^51^. The 1981–2010 period corresponds to a 30-year climatic normal widely used in ecological and biogeographic analyses^47^. We grouped our modeling algorithms by how they handle absences: (i) background-based methods, which contrast presences against the environmental background without assuming true absences, and (ii) pseudo-absence methods, which introduce randomly generated absence points during model fitting^52^. We implemented five algorithms with SSDM in R^31^. For background-based models we used MaxEnt^53^ and support vector machines^54^ (SVMs). For pseudo-absence models, we used Random Forest^55^, generalized linear models^56^ (GLMs), and generalized additive models^57^ (GAM). The number of background and pseudo-absence points were defined following the default configuration of the SSDM package, which balances the number of pseudo-absences with the number of presences and distributes them randomly within the accessible area of each species. This approach, as recommended, has been shown to provide stable and reliable model performance while avoiding biases associated with arbitrary pseudo-absence selection^58^. All modeling was run on the GLORIOSOS computing cluster, using R^59^. For each species–algorithm pair, we fitted suitability models, then produced group-level stacked species maps by summing species probabilities to obtain continuous richness surfaces (pSSDM)^49^.

We retained only species models with AUC ≥ 0.75 as a minimum discrimination threshold^60^ when building the group stacks. We then created two richness maps (one per algorithm group) and combined them by pixel-wise averaging to yield a consensus richness surface. Averaging across groups is intended to temper extrapolation by more permissive methods, such as MaxEnt, and to improve comparability across approaches^7^. To increase statistical stability, we used repeated 80/20 cross-validation with 15 random replicates for each model (summary scripts are included as Supplementary Code S1). To compare the results between the repositories, we first assessed the normality of the predicted species richness values using the Shapiro-Wilk test^61^. As the results indicated that the richness values were not normally distributed, we first calculated Spearman’s correlation to assess the relationship between the two models.

For subsequent environmental analyses, the combined model was used as the primary response surface from which predicted species richness values were extracted. Variable importance was first assessed using a jackknife procedure implemented in the *ssdm* package, allowing us to quantify the relative contribution of each environmental predictor to the richness models by evaluating changes in model performance when variables were excluded or used in isolation^31^. In addition, we explored latitudinal variation in environmental predictors across the Espinhaço Range to characterize how climatic conditions change along the mountain chain and to better contextualize the environmental gradients associated with areas of higher or lower predicted richness. This descriptive approach allowed us to assess how environmental predictors contribute to richness patterns and how their spatial variation aligns with the species richness.

## Supplementary information

Supplementary files

## Data Availability

The complete dataset in the analysis is included in the supplementary file with this submission.

## Code availability

All statistical analyses and visualizations were conducted in R (v4.3.1) using the following packages: CoordinateCleaner, devtools, tmap, dismo, raster, rgdal, randomForest, maptools, sp, rgeos, ncdf4, maps, foreach, doParallel, rnaturalearth, SSDM, magrittr, tidyverse, readxl, dplyr, rgbif, tiff, lme4 and lmerTest. The full R code used in this manuscript is also available in the supplementary files.
