# Supplementary material for: Data source effects on the perceived distribution of ant diversity in the Espinhaço Mountain Range, Brazil
Source: NPJ Biodivers. 2026 May 15;5:19. doi: 10.1038/s44185-026-00135-8 (PMC13234289; doi:10.1038/s44185-026-00135-8)
Supplement: Supplementary file 1 — Supplementary files [file 44185_2026_135_MOESM1_ESM.pdf]

Table S1. Global cleaned dataset

species	lon	lat
Acanthoponera mucronata	-43.598	-19.962
Acromyrmex aspersus	-43.88194444	-21.67888889
Acromyrmex balzani	-44.644375	-21.472643
Acromyrmex balzani	-43.683334	-20.483334
Acromyrmex coronatus	-43.643238	-19.770119
Acromyrmex hispidus	-43.88194444	-21.67888889
Acromyrmex laticeps	-43.683334	-20.483334
Acromyrmex niger	-43.643238	-19.770119
Anochetus neglectus	-44.654343	-21.475533
Atta laevigata	-43.59015	-19.328303
Atta laevigata	-43.601075	-19.304649
Atta laevigata	-43.600244	-19.313662
Atta laevigata	-44.296814	-17.499108
Atta sexdens	-43.643238	-19.770119
Atta sexdens	-43.89027778	-20.06416667
Brachymyrmex heeri	-43.54166667	-20.375
Camponotus arboreus	-43.596642	-18.246442
Camponotus arboreus	-43.619581	-18.144563
Camponotus arboreus	-43.586907	-19.352594
Camponotus atriceps	-43.01198	-16.86154
Camponotus blandus	-43.586907	-19.352594
Camponotus cingulatus	-43.988219	-20.192058
Camponotus cingulatus	-43.988102	-20.192259
Camponotus cingulatus	-43.54166667	-20.375
Camponotus crassus	-43.5	-19.16666667
Camponotus crassus	-43.88194444	-21.67888889
Camponotus crassus	-43.54166667	-20.375
Camponotus genatus	-43.88194444	-21.67888889
Camponotus leydigi	-43.586907	-19.352594
Camponotus melanoticus	-43.88194444	-21.67888889
Camponotus melanoticus	-43.89027778	-20.06416667
Camponotus renggeri	-42.079723	-12.793303

Camponotus renggeri	-42.035958	-12.860979
Camponotus renggeri	-43.88194444	-21.67888889
Camponotus rufipes	-43.505979	-20.381346

Camponotus rufipes	-41.47577	-12.775509
Camponotus rufipes	-43.652817	-20.312585
Camponotus rufipes	-43.977737	-20.088111
Camponotus rufipes	-43.759726	-20.097592
Camponotus rufipes	-43.89027778	-20.06416667
Camponotus rufipes	-43.94282	-20.178639
Camponotus rufipes	-43.916574	-19.957737
Camponotus rufipes	-43.5	-19.16666667
Camponotus rufipes	-43.54166667	-20.375
Camponotus sericeiventris	-43.594909	-19.293626
Camponotus sericeiventris	-43.338374	-18.429736
Camponotus sericeiventris	-43.839436	-20.256663
Camponotus sericeiventris	-43.903633	-19.951072
Camponotus sericeiventris	-43.935665	-21.725636
Camponotus sericeiventris	-43.990547	-18.530145
Camponotus sericeiventris	-44.998325	-21.729435
Camponotus sericeiventris	-43.931857	-19.978181
Camponotus sericeiventris	-43.948038	-20.04496
Camponotus sericeiventris	-43.461143	-18.419353
Camponotus sericeiventris	-43.563561	-17.146151
Camponotus sericeiventris	-44.052662	-20.088362
Camponotus sericeiventris	-43.87997	-19.92811
Camponotus sericeiventris	-43.400137	-18.399088
Camponotus sericeiventris	-43.5	-19.16666667
Centromyrmex brachycola	-44.65061	-21.482533
Cephalotes atratus	-43.841306	-20.257935
Cephalotes atratus	-43.986978	-18.52343
Cephalotes atratus	-43.877404	-20.026322
Cephalotes atratus	-43.497987	-18.416635
Cephalotes atratus	-43.172539	-16.407727
Cephalotes atratus	-43.586907	-19.352594
Cephalotes atratus	-43.5	-19.16666667
Cephalotes clypeatus	-43.586907	-19.352594
Cephalotes depressus	-40.85	-14.85
Cephalotes inca	-40.85	-14.85
Cephalotes pavonii	-43.88194444	-21.67888889
Cephalotes pusillus	-43.405978	-19.476618
Cephalotes pusillus	-43.034064	-16.852664
Cephalotes pusillus	-43.765909	-20.098423
Cephalotes pusillus	-43.643238	-19.770119

Cephalotes pusillus	-41.3698	-12.9963
Cephalotes pusillus	-43.88194444	-21.67888889
Cephalotes pusillus	-43.5	-19.16666667
Cephalotes ustus	-40.85	-14.85
Crematogaster chodati	-43.64324	-19.77012
Crematogaster evallans	-43.87111111	-20.02111111
Crematogaster evallans	-43.89027778	-20.06416667
Crematogaster sericea	-43.88194444	-21.67888889
Diaphoromyrma sofiae	-41.8	-12.81667
Discothyrea sexarticulata	-43.553345	-19.25456
Discothyrea sexarticulata	-43.551598	-19.253029
Discothyrea sexarticulata	-43.55179	-19.252981
Discothyrea sexarticulata	-43.55172	-19.252962
Dorymyrmex brunneus	-43.89027778	-20.06416667
Dorymyrmex goeldii	-43.61667	-19.36668
Dorymyrmex spurius	-43.89027778	-20.06416667
Eciton burchellii	-43.786651	-20.029328
Eciton quadriglume	-43.46	-20.11
Eciton quadriglume	-43.484781	-20.096421
Ectatomma brunneum	-43.586907	-19.352594
Ectatomma edentatum	-43.88194444	-21.67888889
Ectatomma muticum	-43.5	-19.16666667
Ectatomma opaciventre	-43.4875	-18.414752
Ectatomma opaciventre	-43.586907	-19.352594
Ectatomma planidens	-43.6173	-19.3544
Eurhopalothrix spectabilis	-43.553345	-19.25456
Eurhopalothrix spectabilis	-43.55261	-19.253231
Gnamptogenys caelata	-43.9408	-20.0881
Heteroponera dentinodis	-43.88194444	-21.67888889
Heteroponera inermis	-43.88194444	-21.67888889
Hylomyrma balzani	-43.514927	-20.426323
Hylomyrma primavesi	-43.53472	-19.26389
Hylomyrma primavesi	-43.53444	-19.26389
Hypoponera foreli	-43.88194444	-21.67888889
Labidus praedator	-41.458002	-12.781015

Labidus praedator	-43.948856	-20.176769
Labidus praedator	-44.009605	-20.052099
Lachnomyrmex victori	-43.514927	-20.426323
Linepithema cerradense	-43.88194444	-21.67888889
Linepithema humile	-43.54166667	-20.375
Linepithema leucomelas	-43.8833	-21.7
Linepithema micans	-43.5	-20.1333
Linepithema pulex	-43.88194444	-21.67888889
Mycetomoellerius urichii	-41.5741	-12.61725
Myrmelachista bambusarum	-43.505627	-20.398066
Myrmelachista catharinae	-40.83889	-14.841111
Myrmelachista catharinae	-43.550888	-19.247944
Myrmelachista catharinae	-43.50611	-20.428055
Myrmelachista catharinae	-43.643238	-19.770119
Myrmelachista gagatina	-43.514473	-20.42614
Myrmelachista gagatina	-43.508778	-20.434221
Myrmelachista gagatina	-43.514442	-20.42639
Myrmelachista gagatina	-40.83829	-14.841305
Myrmelachista gagatina	-43.514168	-20.426111
Myrmelachista gallicola	-43.51	-20.41
Myrmelachista gallicola	-43.5045	-20.396
Myrmelachista gallicola	-43.5117	-20.39367
Myrmelachista gallicola	-43.50813	-20.39641
Myrmelachista gallicola	-43.514446	-20.440556
Myrmelachista kloetersi	-43.5	-20.1345
Myrmelachista nodigera	-41.37	-13
Myrmelachista nodigera	-43.616943	-19.3675
Myrmelachista rudolphi	-43.5	-20.1345
Myrmelachista ruszkii	-43.586907	-19.352594
Neoponera curvinodis	-43.643238	-19.770119
Neoponera verenae	-43.643288	-19.770119
Neoponera villosa	-43.54166667	-20.375
Octostruma balzani	-43.88194444	-21.67888889
Octostruma rugifera	-43.88194444	-21.67888889
Octostruma stenognatha	-43.514927	-20.426323
Odontomachus chelifer	-43.464747	-20.370645
Odontomachus chelifer	-43.5	-19.16666667
Oxyepoecus myops	-43.403	-19.041
Oxyepoecus rastratus	-43.9361	-20.2953

Pachycondyla harpax	-44.6475	-21.47139
Pachycondyla marginata	-43.643238	-19.770119
Pachycondyla striata	-43.643238	-19.770119
Pachycondyla striata	-43.88194444	-21.67888889
Pachycondyla villosa	-43.71	-18.9
Paratrechina longicornis	-43.009261	-16.861861
Pheidole obscurithorax	-43.88194444	-21.67888889
Pheidole oxyops	-43.5	-19.16666667
Pheidole radoszkowskii	-43.88194444	-21.67888889
Procryptocerus goeldii	-43.5	-20.13333333
Pseudomyrmex gracilis	-43.586907	-19.352594
Pseudomyrmex termitarius	-43.643238	-19.770119
Pyramica crassicornis	-43.551796	-19.252981
Pyramica crassicornis	-43.506466	-20.426695
Sericomyrmex mayri	-41.5005	-12.9053
Sericomyrmex parvulus	-41.6833	-12.55
Sericomyrmex scrobifer	-40.84	-14.84
Solenopsis geminata	-42.1	-12.4
Solenopsis indet	-43.514927	-20.426323
Solenopsis invicta	-43.87111111	-20.02111111
Solenopsis invicta	-43.89027778	-20.06416667
Solenopsis saevissima	-42.1	-12.4
Solenopsis saevissima	-43.5	-19.16666667
Solenopsis substituta	-42.1	-12.4
Solenopsis sulfurea	-41.53552	-13.27808
Solenopsis tridens	-42.1	-12.4
Solenopsis virulens	-43.5	-19.16666667
Strumigenys denticulata	-43.61726	-19.37294
Strumigenys elongata	-43.6181	-19.367664
Strumigenys louisianae	-43.88194444	-21.67888889
Strumigenys saliens	-43.905556	-21.680616
Tapinoma atriceps	-43.55	-19.25
Tapinoma atriceps	-43.52	-19.25

Tapinoma melanocephalum	-43.931855	-19.978165
Tranopelta gilva	-41.3698	-12.9963
Wasmannia affinis	-43.514927	-20.426323
Wasmannia affinis	-43.88194444	-21.67888889
Wasmannia auropunctata	-43.88194444	-21.67888889

Table S2. Regional cleaned dataset

species	lon	lat
Acanthognathus rudis	-40,33972	-14,60186
Acanthoponera mucronata	-43,598	-19,962
Acanthoponera mucronata	-40,85	-14,85
Acanthoponera mucronata	-43,598	-19,962
Acanthostichus kirbyi	-40,85	-14,85
Acromyrmex aspersus	-43,88194444	-21,67888889
Acromyrmex aspersus	-44	-18
Acromyrmex aspersus	-43,88194	-21,67889
Acromyrmex aspersus	-43,5	-20,38
Acromyrmex aspersus	-43,50599	-20,19277
Acromyrmex aspersus	-43,68333	-20,51667
Acromyrmex aspersus	-43,50611	-20,4286
Acromyrmex aspersus	-43,51477	-20,42596
Acromyrmex aspersus	-43,50857	-20,3377
Acromyrmex balzani	-43,60554166	-19,26825246
Acromyrmex balzani	-43,60916399	-19,35970422
Acromyrmex balzani	-43,59419558	-19,29766774
Acromyrmex balzani	-43,58830378	-19,29373311
Acromyrmex balzani	-44,644375	-21,472643
Acromyrmex balzani	-43,683334	-20,483334
Acromyrmex balzani	-44	-18
Acromyrmex coronatus	-43,55709803	-19,29470834
Acromyrmex coronatus	-43,643238	-19,770119
Acromyrmex coronatus	-43,616667	-19,266667
Acromyrmex coronatus	-44	-18
Acromyrmex coronatus	-43,65	-19
Acromyrmex crassispinus	-43,51477	-20,42596
Acromyrmex crassispinus	-43,50806	-20,2875
Acromyrmex crassispinus	-43,50896	-20,43476
Acromyrmex crassispinus	-43,50611	-20,4286
Acromyrmex crassispinus	-43,50857	-20,3377
Acromyrmex crassispinus	-43,51477	-20,42596
Acromyrmex diciger	-44	-18
Acromyrmex hispidus	-43,88194444	-21,67888889
Acromyrmex hispidus	-43,88194	-21,67889
Acromyrmex landolti	-44	-18
Acromyrmex landolti	-43,68333	-20,51667
Acromyrmex landolti	-44	-18
Acromyrmex landolti	-44	-18
Acromyrmex laticeps	-43,683334	-20,483334
Acromyrmex laticeps	-43,50562	-20,4278
Acromyrmex multicinodus	-43,5	-20,38333
Acromyrmex niger	-43,643238	-19,770119
Acromyrmex niger	-44	-18
Acromyrmex niger	-43,5	-20,38

Acromyrmex nigrosetosus	-44	-18
Acromyrmex nigrosetosus	-43,50806	-20,2875
Acromyrmex ospersus	-43,50857	-20,43377
Acromyrmex rugosus	-44	-18
Acromyrmex rugosus	-44	-18
Acromyrmex subterraneus	-43,53618284	-19,26587124
Acromyrmex subterraneus	-43,53812065	-19,26736431
Acromyrmex subterraneus	-43,54217766	-19,25934853
Acromyrmex subterraneus	-43,55222211	-19,24632213
Acromyrmex subterraneus	-43,57628714	-19,23067245
Acromyrmex subterraneus	-43,50870855	-19,19974214
Acromyrmex subterraneus	-43,50768546	-19,21746248
Acromyrmex subterraneus	-43,51038812	-19,21493199
Acromyrmex subterraneus	-43,512842	-19,21340145
Acromyrmex subterraneus	-43,5154595	-19,22641461
Acromyrmex subterraneus	-43,54063815	-19,23901387
Acromyrmex subterraneus	-43,55193109	-19,25301164
Acromyrmex subterraneus	-43,5556887	-19,24451843
Acromyrmex subterraneus	-43,51276833	-19,23850274
Acromyrmex subterraneus	-43,51249022	-19,23886878
Acromyrmex subterraneus	-43,51693237	-19,25473991
Acromyrmex subterraneus	-43,60516305	-19,26909995
Acromyrmex subterraneus	-43,62102337	-19,36582779
Acromyrmex subterraneus	-43,60954595	-19,35895404
Acromyrmex subterraneus	-43,55497532	-19,29484162
Acromyrmex subterraneus	-43,55417476	-19,29365398
Acromyrmex subterraneus	-43,59473756	-19,29644893
Acromyrmex subterraneus	-43,58674466	-19,29347931
Acromyrmex subterraneus	-43,58798761	-19,26227658
Acromyrmex subterraneus	-43,58389892	-19,26227113
Acromyrmex subterraneus	-44	-18
Acromyrmex subterraneus	-43,45882	-20,23164
Acromyrmex subterraneus	-43,616667	-19,266667
Acromyrmex subterraneus	-43,50796	-19,21721
Acromyrmex subterraneus	-43,51284	-19,21324
Acromyrmex subterraneus	-43,51555	-19,22628
Acromyrmex subterraneus	-43,54059	-19,23923
Acromyrmex subterraneus	-43,55207	-19,25299
Acromyrmex subterraneus	-43,55396	-19,24394
Acromyrmex subterraneus	-43,51269	-19,2387
Acromyrmex subterraneus	-43,51695	-19,25501
Acromyrmex subterraneus	-43,54231	-19,25955
Acromyrmex subterraneus	-43,50877	-19,1995
Acromyrmex subterraneus	-44	-18
Anochetus altisquamis	-43,51249022	-19,23886878
Anochetus altisquamis	-43,51276833	-19,23850274
Anochetus altisquamis	-43,51686758	-19,25521592
Anochetus altisquamis	-43,51269	-19,2387

Anochetus altisquamis	-43,51695	-19,25501
Anochetus altisquamis	-44	-18
Anochetus diegensis	-41,66667	-12,73333
Anochetus fumatus	-44	-18
Anochetus inermis	-43,57593703	-19,227891
Anochetus inermis	-43,5771965	-19,23012117
Anochetus inermis	-43,51239273	-19,23875252
Anochetus inermis	-43,5171208	-19,25477696
Anochetus inermis	-43,60672133	-19,26853116
Anochetus inermis	-43,60575238	-19,26979146
Anochetus inermis	-43,60954595	-19,35895404
Anochetus inermis	-43,60916399	-19,35970422
Anochetus inermis	-43,55417476	-19,29365398
Anochetus inermis	-43,58862606	-19,26193929
Anochetus inermis	-43,51269	-19,2387
Anochetus inermis	-43,51695	-19,25501
Anochetus negectus	-44	-18
Anochetus neglectus	-44,654343	-21,475533
Anochetus neglectus	-44,654342	-21,475533
Anochetus neglectus	-44,65434	-21,47553
Anochetus neglectus	-41,35	-13,6
Anochetus neglectus	-43,55	-19,32
Anochetus oriens	-41,35	-13,6
Anochetus simoni	-40,33972	-14,60186
Apterostigmagr. pilosum	-43,616667	-19,266667
Atta bisphaerica	-44	-18
Atta capiguara	-44	-18
Atta laevigata	-43,59015	-19,328303
Atta laevigata	-43,601075	-19,304649
Atta laevigata	-43,600244	-19,313662
Atta laevigata	-44,296814	-17,499108
Atta laevigata	-44	-18
Atta laevigata	-43,594	-19,29
Atta sexdens	-43,5766878	-19,22981079
Atta sexdens	-43,60634255	-19,27007644
Atta sexdens	-43,60954595	-19,35895404
Atta sexdens	-43,60916399	-19,35970422
Atta sexdens	-43,55709803	-19,29470834
Atta sexdens	-43,58944908	-19,26236669
Atta sexdens	-43,58887115	-19,26359874
Atta sexdens	-43,58374184	-19,2626567
Atta sexdens	-43,643238	-19,770119
Atta sexdens	-43,89027778	-20,06416667
Atta sexdens	-43,616667	-19,266667
Atta sexdens	-44	-18
Atta sexdens	-43,68333	-20,51667
Atta sexdens	-43,53884024	-19,26565348
Atta sexdens	-43,57297277	-19,22959956

Atta sexdens	-43,57665964	-19,22886573
Atta sexdens	-43,60725786	-19,266002
Atta sexdens	-43,60602505	-19,27408459
Atta sexdens	-43,60935476	-19,35928596
Atta sexdens	-43,58887115	-19,26359874
Atta sexdens	-43,58354487	-19,2630799
Atta sexdens	-44	-18
Basiceros convexiceps	-44	-18
Basiceros disciger	-43,54259047	-19,25948038
Basiceros disciger	-43,51067009	-19,21557186
Basiceros disciger	-43,51566595	-19,22640212
Basiceros disciger	-43,51038812	-19,21493199
Basiceros disciger	-43,61930802	-19,3672191
Basiceros disciger	-43,62044871	-19,36814563
Basiceros disciger	-43,51081	-19,20915
Basiceros disciger	-43,51555	-19,22628
Basiceros disciger	-43,54231	-19,25955
Basiceros disciger	-44	-18
Basiceros disciger	-40,33972	-14,60186
Basiceros disciger	-43,86667	-21,66667
Basiceros scambognathus	-40,43279	-13,43588
Basiceros scambognathus	-44	-18
Belonopelta curvata	-44	-18
Brachymyrmex admostus	-44	-18
Brachymyrmex admotus	-44	-18
Brachymyrmex admotus	-43,5	-20,13333
Brachymyrmex cordemoyi	-43,5386581	-19,26607518
Brachymyrmex cordemoyi	-43,57703196	-19,22896966
Brachymyrmex cordemoyi	-43,57761668	-19,22925783
Brachymyrmex cordemoyi	-43,60682149	-19,26881321
Brachymyrmex cordemoyi	-43,55433955	-19,29329633
Brachymyrmex cordemoyi	-43,5574266	-19,2939668
Brachymyrmex cordemoyi	-43,58993556	-19,2939704
Brachymyrmex cordemoyi	-43,58821459	-19,26322608
Brachymyrmex cordemoyi	-43,53637193	-19,26544771
Brachymyrmex cordemoyi	-43,53812065	-19,26736431
Brachymyrmex cordemoyi	-43,5771965	-19,23012117
Brachymyrmex cordemoyi	-43,57689064	-19,23097428
Brachymyrmex cordemoyi	-43,60535257	-19,26870232
Brachymyrmex cordemoyi	-43,62007379	-19,36781379
Brachymyrmex cordemoyi	-43,62044871	-19,36814563
Brachymyrmex cordemoyi	-43,60954595	-19,35895404
Brachymyrmex cordemoyi	-43,55497532	-19,29484162
Brachymyrmex cordemoyi	-43,58792231	-19,29276416
Brachymyrmex cordemoyi	-43,5929424	-19,29714278
Brachymyrmex cordemoyi	-43,58674466	-19,29347931
Brachymyrmex cordemoyi	-43,58821459	-19,26322608
Brachymyrmex cordemoyi	-43,58887115	-19,26359874

Brachymyrmex cordemoyi	-43,58354487	-19,2630799
Brachymyrmex cordemoyi	-43,594	-19,29
Brachymyrmex feitosai	-43,86667	-21,66667
Brachymyrmex heeri	-43,54166667	-20,375
Brachymyrmex heeri	-43,54167	-20,375
Brachymyrmex heeri	-43,5	-20,4
Brachymyrmex heeri	-43,5	-20,13333
Brachymyrmex heeri	-43,56667	-18,18333
Brachymyrmex patagonicus	-44,03111	-20,05933
Brachymyrmex patagonicus	-44,01166	-20,05977
Brachymyrmex patagonicus	-44,02138	-20,05955
Brachymyrmex pictus	-43,53637193	-19,26544771
Brachymyrmex pictus	-43,5386581	-19,26607518
Brachymyrmex pictus	-43,5752322	-19,22795009
Brachymyrmex pictus	-43,57356537	-19,22774909
Brachymyrmex pictus	-43,60516305	-19,26909995
Brachymyrmex pictus	-43,60497655	-19,26951637
Brachymyrmex pictus	-43,61970456	-19,36750618
Brachymyrmex pictus	-43,62007379	-19,36781379
Brachymyrmex pictus	-43,60954595	-19,35895404
Brachymyrmex pictus	-43,55453208	-19,29292551
Brachymyrmex pictus	-43,55378919	-19,29447139
Brachymyrmex pictus	-43,59380012	-19,29607602
Brachymyrmex pictus	-43,58749366	-19,29428162
Brachymyrmex pictus	-43,58635783	-19,29374769
Brachymyrmex pictus	-43,58906569	-19,26318266
Brachymyrmex pictus	-43,58846588	-19,26102131
Brachymyrmex pictus	-43,58617628	-19,26141224
Brachymyrmex pictus	-43,56667	-18,18333
Brachymyrmex pictus	-43,5	-20,4
Brachymyrmex pictus	-43,594	-19,29
Camponotus abdominalis	-44	-18
Camponotus abscisus	-43,50878	-20,43416
Camponotus acuta	-43,50836	-20,43379
Camponotus ager	-43,50667	-20,4286
Camponotus ager	-43,50611	-20,4286
Camponotus ager	-43,5	-20,4
Camponotus ager	-43,50599	-20,19277
Camponotus ager	-43,50718	-20,19246
Camponotus ager	-43,50857	-20,43377
Camponotus alboannulatus	-43,54167	-20,375
Camponotus alboannulatus	-43,54166	-20,375
Camponotus alboannulatus	-43,50857	-20,43377
Camponotus arboreus	-43,60685444	-19,26823025
Camponotus arboreus	-43,60603527	-19,27437158
Camponotus arboreus	-43,596642	-18,246442
Camponotus arboreus	-43,619581	-18,144563
Camponotus arboreus	-43,586907	-19,352594

Camponotus arboreus	-43,616667	-19,266667
Camponotus arboreus	-44	-18
Camponotus arboreus	-43,56667	-18,18333
Camponotus atriceps	-43,57320461	-19,22974491
Camponotus atriceps	-43,60600694	-19,2747227
Camponotus atriceps	-43,588673	-19,26015847
Camponotus atriceps	-43,01198	-16,86154
Camponotus atriceps	-43,5	-20,4
Camponotus atriceps	-43,45882	-20,23164
Camponotus balzani	-43,50562	-20,4278
Camponotus balzani	-43,50857	-20,43377
Camponotus bidens	-43,58697667	-19,26169588
Camponotus blandus	-43,53812065	-19,26736431
Camponotus blandus	-43,53379182	-19,26554787
Camponotus blandus	-43,57761668	-19,22925783
Camponotus blandus	-43,57590409	-19,23054487
Camponotus blandus	-43,60643718	-19,26963774
Camponotus blandus	-43,60935476	-19,35928596
Camponotus blandus	-43,5539938	-19,29405481
Camponotus blandus	-43,59456489	-19,29685922
Camponotus blandus	-43,58713743	-19,29451112
Camponotus blandus	-43,58879512	-19,26152489
Camponotus blandus	-43,58906569	-19,26318266
Camponotus blandus	-43,58318394	-19,2638813
Camponotus blandus	-43,5402354	-19,23933799
Camponotus blandus	-43,51291836	-19,2386407
Camponotus blandus	-43,586907	-19,352594
Camponotus blandus	-43,54059	-19,23923
Camponotus blandus	-43,51269	-19,2387
Camponotus blandus	-44	-18
Camponotus blandus	-43,594	-19,29
Camponotus bonariensis	-43,88194	-21,67889
Camponotus bonariensis	-43,88194	-21,67889
Camponotus cameranoi	-43,50667	-20,4286
Camponotus cf. fastigatus	-43,50879	-20,43476
Camponotus cf. fastigatus	-43,51443	-20,42619
Camponotus cingulatus	-43,57703799	-19,23056072
Camponotus cingulatus	-43,55193109	-19,25301164
Camponotus cingulatus	-43,51291836	-19,2386407
Camponotus cingulatus	-43,55709803	-19,29470834
Camponotus cingulatus	-43,58374184	-19,2626567
Camponotus cingulatus	-43,988219	-20,192058
Camponotus cingulatus	-43,988102	-20,192259
Camponotus cingulatus	-43,54166667	-20,375
Camponotus cingulatus	-43,616667	-19,266667
Camponotus cingulatus	-43,55207	-19,25299
Camponotus cingulatus	-43,51269	-19,2387
Camponotus cingulatus	-43,54167	-20,375

Camponotus cingulatus	-43,49673	-20,2269
Camponotus cingulatus	-43,50808	-20,21398
Camponotus cingulatus	-43,50581	-20,19359
Camponotus cingulatus	-43,51206	-20,21405
Camponotus cingulatus	-43,51826	-20,20577
Camponotus cingulatus	-43,51696	-20,19747
Camponotus cingulatus	-43,50599	-20,19277
Camponotus cingulatus	-43,5147	-20,18679
Camponotus cingulatus	-43,51557	-20,16975
Camponotus cingulatus	-40,33972	-14,60186
Camponotus cingulatus	-43,50667	-20,4286
Camponotus cingulatus	-43,50857	-20,3377
Camponotus cingulatus	-43,51477	-20,42596
Camponotus clypeatus	-44	-18
Camponotus coriolanus	-43,5	-20,13333
Camponotus coriolanus	-44	-18
Camponotus crassus	-43,53848334	-19,26651581
Camponotus crassus	-43,54255216	-19,25967802
Camponotus crassus	-43,55093289	-19,24823379
Camponotus crassus	-43,57379302	-19,2279412
Camponotus crassus	-43,57539447	-19,23100102
Camponotus crassus	-43,57703196	-19,22896966
Camponotus crassus	-43,57628714	-19,23067245
Camponotus crassus	-43,55382892	-19,24401468
Camponotus crassus	-43,51283647	-19,21323683
Camponotus crassus	-43,5402354	-19,23933799
Camponotus crassus	-43,55193109	-19,25301164
Camponotus crassus	-43,51239273	-19,23875252
Camponotus crassus	-43,60575238	-19,26979146
Camponotus crassus	-43,60935476	-19,35928596
Camponotus crassus	-43,60916399	-19,35970422
Camponotus crassus	-43,55417476	-19,29365398
Camponotus crassus	-43,59493964	-19,29602548
Camponotus crassus	-43,59456489	-19,29685922
Camponotus crassus	-43,58821459	-19,26322608
Camponotus crassus	-43,58887115	-19,26359874
Camponotus crassus	-43,58318394	-19,2638813
Camponotus crassus	-43,5	-19,16666667
Camponotus crassus	-43,88194444	-21,67888889
Camponotus crassus	-43,54166667	-20,375
Camponotus crassus	-43,616667	-19,266667
Camponotus crassus	-43,50972	-20,43833
Camponotus crassus	-43,54059	-19,23923
Camponotus crassus	-43,55207	-19,25299
Camponotus crassus	-43,51269	-19,2387
Camponotus crassus	-43,54231	-19,25955
Camponotus crassus	-43,55396	-19,24394
Camponotus crassus	-41,66667	-12,73333

Camponotus crassus	-44,03111	-20,05933
Camponotus crassus	-44,01166	-20,05977
Camponotus crassus	-44	-18
Camponotus crassus	-43,89693	-21,71422
Camponotus crassus	-43,88333	-21,7
Camponotus crassus	-43,88194	-21,67889
Camponotus crassus	-43,54166	-20,375
Camponotus crassus	-43,5	-19,16667
Camponotus crassus	-44,02138	-20,05955
Camponotus crassus	-43,5	-20,4
Camponotus crassus	-43,50871	-20,40742
Camponotus crassus	-43,594	-19,29
Camponotus crassus	-43,56667	-18,18333
Camponotus crassus	-43,50562	-20,4278
Camponotus crassus	-43,50857	-20,3377
Camponotus crassus	-43,51477	-20,42596
Camponotus diversipalpus	-43,50562	-20,4278
Camponotus diversipalpus	-43,50857	-20,43377
Camponotus fastigatus	-43,57703799	-19,23056072
Camponotus fastigatus	-43,60935476	-19,35928596
Camponotus fastigatus	-43,60916399	-19,35970422
Camponotus fastigatus	-43,5086	-20,43467
Camponotus fastigatus	-43,54167	-20,375
Camponotus fastigatus	-43,50653	-20,42841
Camponotus fastigatus	-43,51429	-20,42627
Camponotus genatus	-43,88194444	-21,67888889
Camponotus genatus	-44,03111	-20,05933
Camponotus genatus	-44,01166	-20,05977
Camponotus genatus	-44	-18
Camponotus genatus	-43,88333	-21,7
Camponotus genatus	-43,88194	-21,67889
Camponotus genatus	-44,02138	-20,05955
Camponotus inca rector	-44	-18
Camponotus jheringi	-44	-18
Camponotus latangulus	-43,58839899	-19,26072735
Camponotus lespesii	-43,54212686	-19,25955221
Camponotus lespesii	-43,55222211	-19,24632213
Camponotus lespesii	-43,5744194	-19,22840908
Camponotus lespesii	-43,5556887	-19,24451843
Camponotus lespesii	-43,50898289	-19,19937015
Camponotus lespesii	-43,50784513	-19,21756575
Camponotus lespesii	-43,51104224	-19,21521471
Camponotus lespesii	-43,512842	-19,21340145
Camponotus lespesii	-43,51543218	-19,22601705
Camponotus lespesii	-43,5402354	-19,23933799
Camponotus lespesii	-43,55193109	-19,25301164
Camponotus lespesii	-43,51249022	-19,23886878
Camponotus lespesii	-43,51686758	-19,25521592

Camponotus lespesii	-43,60725786	-19,266002
Camponotus lespesii	-43,62044871	-19,36814563
Camponotus lespesii	-43,616667	-19,266667
Camponotus lespesii	-43,50796	-19,21721
Camponotus lespesii	-43,51284	-19,21324
Camponotus lespesii	-43,51555	-19,22628
Camponotus lespesii	-43,54059	-19,23923
Camponotus lespesii	-43,55207	-19,25299
Camponotus lespesii	-43,5523	-19,24623
Camponotus lespesii	-43,51269	-19,2387
Camponotus lespesii	-43,51695	-19,25501
Camponotus lespesii	-43,54231	-19,25955
Camponotus lespesii	-43,50877	-19,1995
Camponotus lespesii	-43,50576	-20,21305
Camponotus lespesii	-43,50581	-20,19359
Camponotus lespesii	-43,49673	-20,2269
Camponotus lespesii	-43,51696	-20,19747
Camponotus leydigi	-43,57689064	-19,23097428
Camponotus leydigi	-43,60535257	-19,26870232
Camponotus leydigi	-43,60634339	-19,26858036
Camponotus leydigi	-43,60575238	-19,26979146
Camponotus leydigi	-43,60634255	-19,27007644
Camponotus leydigi	-43,60610191	-19,27049839
Camponotus leydigi	-43,586907	-19,352594
Camponotus leydigi	-44	-18
Camponotus leydigi	-43,594	-19,29
Camponotus macrocephalus	-44	-18
Camponotus melanoticus	-43,53454082	-19,2638678
Camponotus melanoticus	-43,54255216	-19,25967802
Camponotus melanoticus	-43,5386581	-19,26607518
Camponotus melanoticus	-43,55219219	-19,24620646
Camponotus melanoticus	-43,57689064	-19,23097428
Camponotus melanoticus	-43,50886052	-19,19928314
Camponotus melanoticus	-43,51099782	-19,21516165
Camponotus melanoticus	-43,51566595	-19,22640212
Camponotus melanoticus	-43,54063815	-19,23901387
Camponotus melanoticus	-43,55204844	-19,2528342
Camponotus melanoticus	-43,5556887	-19,24451843
Camponotus melanoticus	-43,51291836	-19,2386407
Camponotus melanoticus	-43,51686758	-19,25521592
Camponotus melanoticus	-43,60682149	-19,26881321
Camponotus melanoticus	-43,60603527	-19,27437158
Camponotus melanoticus	-43,62065004	-19,36671224
Camponotus melanoticus	-43,62138044	-19,36499219
Camponotus melanoticus	-43,60935476	-19,35928596
Camponotus melanoticus	-43,55659034	-19,29551871
Camponotus melanoticus	-43,55433955	-19,29329633
Camponotus melanoticus	-43,59201838	-19,29497782

Camponotus melanoticus	-43,58790983	-19,29397836
Camponotus melanoticus	-43,58824267	-19,26276759
Camponotus melanoticus	-43,58318394	-19,2638813
Camponotus melanoticus	-43,55222211	-19,24632213
Camponotus melanoticus	-43,88194444	-21,67888889
Camponotus melanoticus	-43,89027778	-20,06416667
Camponotus melanoticus	-43,616667	-19,266667
Camponotus melanoticus	-43,51081	-19,20915
Camponotus melanoticus	-43,51555	-19,22628
Camponotus melanoticus	-43,54059	-19,23923
Camponotus melanoticus	-43,55207	-19,25299
Camponotus melanoticus	-43,5523	-19,24623
Camponotus melanoticus	-43,51269	-19,2387
Camponotus melanoticus	-43,51695	-19,25501
Camponotus melanoticus	-43,54231	-19,25955
Camponotus melanoticus	-43,50877	-19,1995
Camponotus melanoticus	-41,66667	-12,73333
Camponotus melanoticus	-43,88194	-21,67889
Camponotus melanoticus	-43,68333	-20,51667
Camponotus melanoticus	-43,86667	-21,66667
Camponotus melanoticus	-43,56667	-18,18333
Camponotus melanoticus	-43,50562	-20,4278
Camponotus melanoticus	-43,50857	-20,43377
Camponotus myrmaphaneus	-43,50562	-20,4278
Camponotus myrmaphaneus	-43,50857	-20,43377
Camponotus novagrandensis	-43,50857	-20,3377
Camponotus novagrandensis	-43,50562	-20,4278
Camponotus novagrandensis	-43,51477	-20,42596
Camponotus novogranadensis	-43,53418258	-19,26471345
Camponotus novogranadensis	-43,57761668	-19,22925783
Camponotus novogranadensis	-43,60643718	-19,26963774
Camponotus novogranadensis	-43,60935476	-19,35928596
Camponotus novogranadensis	-43,5574266	-19,2939668
Camponotus novogranadensis	-43,59419558	-19,29766774
Camponotus novogranadensis	-43,58713743	-19,29451112
Camponotus novogranadensis	-43,58887115	-19,26359874
Camponotus novogranadensis	-43,58862606	-19,26193929
Camponotus novogranadensis	-43,58318394	-19,2638813
Camponotus novogranadensis	-44	-18
Camponotus novogranadensis	-43,51444	-20,44056
Camponotus novogranadensis	-43,5	-20,4
Camponotus novogranadensis	-43,51444	-20,44055
Camponotus novograndensis	-43,56667	-18,18333
Camponotus personatus	-43,68333	-20,51667
Camponotus propinquus	-44	-18
Camponotus punctulatus	-43,60672133	-19,26853116
Camponotus renggeri	-43,53830405	-19,26693684
Camponotus renggeri	-43,5768541	-19,22937987

Camponotus renggeri	-43,60558407	-19,27022221
Camponotus renggeri	-43,60935476	-19,35928596
Camponotus renggeri	-43,55531571	-19,29406738
Camponotus renggeri	-43,59380012	-19,29607602
Camponotus renggeri	-43,58924758	-19,26275661
Camponotus renggeri	-43,58862606	-19,26193929
Camponotus renggeri	-43,58354487	-19,2630799
Camponotus renggeri	-42,079723	-12,793303
Camponotus renggeri	-42,035958	-12,860979
Camponotus renggeri	-43,88194444	-21,67888889
Camponotus renggeri	-40,43279	-13,43588
Camponotus renggeri	-44	-18
Camponotus renggeri	-43,88333	-21,7
Camponotus renggeri	-43,88194	-21,67889
Camponotus renggeri	-43,49673	-20,2269
Camponotus renggeri	-43,56667	-18,18333
Camponotus renggeri	-43,50857	-20,43377
Camponotus renggeri	-43,50611	-20,4286
Camponotus rufipes	-43,53398041	-19,26513791
Camponotus rufipes	-43,54217766	-19,25934853
Camponotus rufipes	-43,53830405	-19,26693684
Camponotus rufipes	-43,55412891	-19,24364579
Camponotus rufipes	-43,57610467	-19,23012905
Camponotus rufipes	-43,50898289	-19,19937015
Camponotus rufipes	-43,50802568	-19,21711237
Camponotus rufipes	-43,51104224	-19,21521471
Camponotus rufipes	-43,51328122	-19,21331612
Camponotus rufipes	-43,5154595	-19,22641461
Camponotus rufipes	-43,5402354	-19,23933799
Camponotus rufipes	-43,55204844	-19,2528342
Camponotus rufipes	-43,55556205	-19,24489209
Camponotus rufipes	-43,51276833	-19,23850274
Camponotus rufipes	-43,5171208	-19,25477696
Camponotus rufipes	-43,60554166	-19,26825246
Camponotus rufipes	-43,62065004	-19,36671224
Camponotus rufipes	-43,62102337	-19,36582779
Camponotus rufipes	-43,60954595	-19,35895404
Camponotus rufipes	-43,55552307	-19,29367712
Camponotus rufipes	-43,55453208	-19,29292551
Camponotus rufipes	-43,59493964	-19,29602548
Camponotus rufipes	-43,58713341	-19,29322399
Camponotus rufipes	-43,58862606	-19,26193929
Camponotus rufipes	-43,58374184	-19,2626567
Camponotus rufipes	-43,505979	-20,381346
Camponotus rufipes	-41,47577	-12,775509
Camponotus rufipes	-43,652817	-20,312585
Camponotus rufipes	-43,977737	-20,088111
Camponotus rufipes	-43,759726	-20,097592

Camponotus rufipes	-43,877641	-20,026227
Camponotus rufipes	-43,94282	-20,178639
Camponotus rufipes	-43,916574	-19,957737
Camponotus rufipes	-43,5	-19,16666667
Camponotus rufipes	-43,89027778	-20,06416667
Camponotus rufipes	-43,54166667	-20,375
Camponotus rufipes	-44	-18
Camponotus rufipes	-43,616667	-19,266667
Camponotus rufipes	-43,50836	-20,43379
Camponotus rufipes	-43,50877	-19,1995
Camponotus rufipes	-43,51284	-19,21324
Camponotus rufipes	-43,51081	-19,20915
Camponotus rufipes	-43,51555	-19,22628
Camponotus rufipes	-43,54059	-19,23923
Camponotus rufipes	-43,55207	-19,25299
Camponotus rufipes	-43,55089	-19,24794
Camponotus rufipes	-43,51269	-19,2387
Camponotus rufipes	-43,51695	-19,25501
Camponotus rufipes	-43,54231	-19,25955
Camponotus rufipes	-43,55396	-19,24394
Camponotus rufipes	-40,43279	-13,43588
Camponotus rufipes	-44,03111	-20,05933
Camponotus rufipes	-44	-18
Camponotus rufipes	-43,89693	-21,71422
Camponotus rufipes	-43,88333	-21,7
Camponotus rufipes	-43,50972	-20,43833
Camponotus rufipes	-43,54167	-20,375
Camponotus rufipes	-43,5	-20,38333
Camponotus rufipes	-43,5	-19,16667
Camponotus rufipes	-44,02138	-20,05955
Camponotus rufipes	-43,5	-20,4
Camponotus rufipes	-43,49673	-20,2269
Camponotus rufipes	-43,5147	-20,18679
Camponotus rufipes	-43,68333	-20,51667
Camponotus rufipes	-43,594	-19,29
Camponotus rufipes	-43,86667	-21,66667
Camponotus rufipes	-43,56667	-18,18333
Camponotus rufipes	-43,50562	-20,4278
Camponotus rufipes	-43,5144	-20,42649
Camponotus rufipes	-43,51477	-20,42596
Camponotus rufipes	-43,50857	-20,3377
Camponotus rufipes	-43,53848334	-19,26651581
Camponotus rufipes	-43,5771965	-19,23012117
Camponotus rufipes	-43,57665964	-19,22886573
Camponotus rufipes	-43,60575238	-19,26979146
Camponotus rufipes	-43,55681053	-19,29509978
Camponotus rufipes	-43,55725486	-19,29432621
Camponotus rufipes	-43,59380012	-19,29607602

Camponotus rufipes	-43,58971546	-19,29438681
Camponotus rufipes	-43,58824267	-19,26276759
Camponotus rufipes	-43,58879512	-19,26152489
Camponotus scipio	-43,68333	-20,51667
Camponotus senex	-43,53848334	-19,26651581
Camponotus senex	-43,53379182	-19,26554787
Camponotus senex	-43,57665964	-19,22886573
Camponotus senex	-43,57628714	-19,23067245
Camponotus senex	-43,50768546	-19,21746248
Camponotus senex	-43,51283647	-19,21323683
Camponotus senex	-43,60477941	-19,26995122
Camponotus senex	-43,60954595	-19,35895404
Camponotus senex	-43,60935476	-19,35928596
Camponotus senex	-43,55417476	-19,29365398
Camponotus senex	-43,58951974	-19,29479929
Camponotus senex	-43,59380012	-19,29607602
Camponotus senex	-43,5929424	-19,29714278
Camponotus senex	-43,58944908	-19,26236669
Camponotus senex	-43,58869806	-19,26403485
Camponotus senex	-43,58374184	-19,2626567
Camponotus senex	-43,58318394	-19,2638813
Camponotus senex	-43,50667	-20,4286
Camponotus senex	-43,50611	-20,4286
Camponotus senex	-43,50857	-20,3377
Camponotus senex	-43,50562	-20,4278
Camponotus senex	-43,51477	-20,42596
Camponotus senex	-43,51444	-20,44056
Camponotus senex	-43,54167	-20,375
Camponotus senex	-43,5147	-20,18679
Camponotus senex	-43,52055	-20,18055
Camponotus senex	-43,51557	-20,16975
Camponotus senex	-43,50599	-20,19277
Camponotus senex	-43,50808	-20,21398
Camponotus senex	-43,50857	-20,43377
Camponotus sericeiventris	-43,60739406	-19,26632957
Camponotus sericeiventris	-43,60725786	-19,266002
Camponotus sericeiventris	-43,60723883	-19,26576605
Camponotus sericeiventris	-43,594909	-19,293626
Camponotus sericeiventris	-43,338374	-18,429736
Camponotus sericeiventris	-43,839157	-20,256665
Camponotus sericeiventris	-43,903342	-19,951102
Camponotus sericeiventris	-43,935665	-21,725636
Camponotus sericeiventris	-43,990547	-18,530145
Camponotus sericeiventris	-44,998325	-21,729435
Camponotus sericeiventris	-43,931857	-19,978181
Camponotus sericeiventris	-43,948038	-20,04496
Camponotus sericeiventris	-43,461143	-18,419353
Camponotus sericeiventris	-43,563561	-17,146151

Camponotus sericeiventris	-44,052662	-20,088362
Camponotus sericeiventris	-43,87997	-19,92811
Camponotus sericeiventris	-43,400137	-18,399088
Camponotus sericeiventris	-43,5	-19,16666667
Camponotus sericeiventris	-44	-18
Camponotus sericeiventris	-43,5	-19,16667
Camponotus sericeiventris	-43,50581	-20,19359
Camponotus sericeiventris	-43,50718	-20,19246
Camponotus sericeventris	-43,58862606	-19,26193929
Camponotus sexguttatus	-43,60612437	-19,27350305
Camponotus sexguttatus	-43,60600694	-19,2747227
Camponotus tenuiscapus	-43,50857	-20,3377
Camponotus tenuiscapus	-43,50562	-20,4278
Camponotus tenuiscapus	-43,51477	-20,42596
Camponotus terbimaculatus	-44	-18
Camponotus trapeziceps	-43,53848334	-19,26651581
Camponotus trapeziceps	-43,57650792	-19,22930259
Camponotus trapeziceps	-43,5170695	-19,25526789
Camponotus trapeziceps	-43,60664757	-19,26921252
Camponotus trapeziceps	-43,60916399	-19,35970422
Camponotus trapeziceps	-43,55659034	-19,29551871
Camponotus trapeziceps	-43,5929424	-19,29714278
Camponotus trapeziceps	-43,58944908	-19,26236669
Camponotus trapeziceps	-43,58879512	-19,26152489
Camponotus trapeziceps	-43,58374184	-19,2626567
Camponotus trapeziceps	-43,594	-19,29
Camponotus tripartitus	-43,60707195	-19,268001
Camponotus tripartitus	-43,60558407	-19,27022221
Camponotus tripartitus	-43,60935476	-19,35928596
Camponotus tripartitus	-44	-18
Camponotus vittatus	-43,53884024	-19,26565348
Camponotus vittatus	-43,57665964	-19,22886573
Camponotus vittatus	-43,60535257	-19,26870232
Camponotus vittatus	-43,60935476	-19,35928596
Camponotus vittatus	-43,55453208	-19,29292551
Camponotus vittatus	-43,59456489	-19,29685922
Camponotus vittatus	-43,58887115	-19,26359874
Camponotus vittatus	-43,58879512	-19,26152489
Camponotus vittatus	-43,594	-19,29
Camponotus vittatus	-43,50667	-20,4286
Camponotus vittatus	-43,50611	-20,4286
Camponotus westermanni	-43,51283647	-19,21323683
Camponotus westermanni	-43,594	-19,29
Camponotus westermanni	-43,56667	-18,18333
Campotnous fastigatus	-43,50562	-20,4278
Campotnous fastigatus	-43,51477	-20,42596
Campotnous fastigatus	-43,50857	-20,3377
Carebara brasiliana	-43,50898289	-19,19937015

Carebara brasiliana	-43,50877	-19,1995
Carebara urichi	-43,57320461	-19,22974491
Carebara urichi	-43,58853185	-19,26043608
Centromyrmex brachycola	-44,65061	-21,482533
Centromyrmex brachycola	-44	-18
Centromyrmex brachycola	-43,51557	-20,16975
Cephalote pusillus	-43,50857	-20,3377
Cephalotes angustus	-44	-18
Cephalotes angustus	-44	-18
Cephalotes atratus	-43,51283647	-19,21323683
Cephalotes atratus	-43,60643718	-19,26963774
Cephalotes atratus	-43,841306	-20,257935
Cephalotes atratus	-43,986978	-18,52343
Cephalotes atratus	-43,877404	-20,026322
Cephalotes atratus	-43,497987	-18,416635
Cephalotes atratus	-43,172539	-16,407727
Cephalotes atratus	-43,586907	-19,352594
Cephalotes atratus	-43,5	-19,16666667
Cephalotes atratus	-43,616667	-19,266667
Cephalotes atratus	-40,43279	-13,43588
Cephalotes atratus	-44	-18
Cephalotes atratus	-43,5	-19,16667
Cephalotes atratus	-44,02138	-20,05955
Cephalotes betoi	-43,55569591	-19,29324931
Cephalotes borgmeieri	-44	-18
Cephalotes clypeatus	-43,586907	-19,352594
Cephalotes clypeatus	-44	-18
Cephalotes depressus	-40,85	-14,85
Cephalotes depressus	-44	-18
Cephalotes depressus	-43,89693	-21,71422
Cephalotes depressus	-40,85	-14,85
Cephalotes eduarduli	-44	-18
Cephalotes eduarduli	-43,594	-19,29
Cephalotes inca	-40,85	-14,85
Cephalotes inca	-40,85	-14,85
Cephalotes maculatus	-44,01166	-20,05977
Cephalotes maculatus	-44	-18
Cephalotes maculatus	-43,56667	-18,18333
Cephalotes minutus	-43,60707195	-19,268001
Cephalotes minutus	-40,43279	-13,43588
Cephalotes minutus	-44	-18
Cephalotes minutus	-43,88333	-21,7
Cephalotes minutus	-44	-18
Cephalotes pallens	-44	-18
Cephalotes pallidicephalus	-44	-18
Cephalotes pallidoides	-43,60610191	-19,27049839
Cephalotes patellaris	-44	-18
Cephalotes pavonii	-43,88194444	-21,67888889

Cephalotes pavonii	-43,88194	-21,67889
Cephalotes pellans	-43,57581122	-19,22815804
Cephalotes pellans	-44	-18
Cephalotes persilimilis	-44	-18
Cephalotes persimilis	-43,50878	-20,43483
Cephalotes persimilis	-43,50878	-20,43416
Cephalotes persimilis	-43,50562	-20,4278
Cephalotes persimilis	-43,50536	-20,42768
Cephalotes persimplex	-44	-18
Cephalotes pineli	-43,50667	-20,4286
Cephalotes pinelii	-44	-18
Cephalotes pinelii	-44	-18
Cephalotes pusillus	-43,57297277	-19,22959956
Cephalotes pusillus	-43,5768541	-19,22937987
Cephalotes pusillus	-43,5771965	-19,23012117
Cephalotes pusillus	-43,57590409	-19,23054487
Cephalotes pusillus	-43,50768546	-19,21746248
Cephalotes pusillus	-43,51054427	-19,21544027
Cephalotes pusillus	-43,51283647	-19,21323683
Cephalotes pusillus	-43,60609579	-19,26899141
Cephalotes pusillus	-43,60602505	-19,27408459
Cephalotes pusillus	-43,60954595	-19,35895404
Cephalotes pusillus	-43,60916399	-19,35970422
Cephalotes pusillus	-43,55725486	-19,29432621
Cephalotes pusillus	-43,5913195	-19,29668278
Cephalotes pusillus	-43,5929424	-19,29714278
Cephalotes pusillus	-43,58792231	-19,29276416
Cephalotes pusillus	-43,58821459	-19,26322608
Cephalotes pusillus	-43,58389892	-19,26227113
Cephalotes pusillus	-43,58318394	-19,2638813
Cephalotes pusillus	-43,405978	-19,476618
Cephalotes pusillus	-43,034031	-16,852672
Cephalotes pusillus	-43,765909	-20,098423
Cephalotes pusillus	-43,643238	-19,770119
Cephalotes pusillus	-41,3698	-12,9963
Cephalotes pusillus	-43,88194444	-21,67888889
Cephalotes pusillus	-43,5	-19,16666667
Cephalotes pusillus	-43,616667	-19,266667
Cephalotes pusillus	-40,43279	-13,43588
Cephalotes pusillus	-41,66667	-12,73333
Cephalotes pusillus	-44	-18
Cephalotes pusillus	-43,88194	-21,67889
Cephalotes pusillus	-43,54167	-20,375
Cephalotes pusillus	-43,5087	-20,43404
Cephalotes pusillus	-43,5	-19,16667
Cephalotes pusillus	-43,5	-20,4
Cephalotes pusillus	-43,54166	-20,375
Cephalotes pusillus	-43,594	-19,29

Cephalotes pusillus	-41,3698	-12,9963
Cephalotes pusillus	-43,56667	-18,18333
Cephalotes pusillus	-43,50821	-20,43387
Cephalotes pusillus	-44	-18
Cephalotes striativentris	-44	-18
Cephalotes ustus	-40,85	-14,85
Cephalotes ustus	-40,43279	-13,43588
Cephalotes ustus	-44	-18
Cephalotes ustus	-40,85	-14,85
Cephalotes ustus	-44	-18
Basiceros scambognathus	-41,66667	-12,73333
Crematogaster acuata	-43,51477	-20,42596
Crematogaster acuata	-43,50857	-20,3377
Crematogaster acuta	-43,53830405	-19,26693684
Crematogaster acuta	-43,57650792	-19,22930259
Crematogaster acuta	-43,57610467	-19,23012905
Crematogaster acuta	-43,60634255	-19,27007644
Crematogaster acuta	-43,60916399	-19,35970422
Crematogaster acuta	-43,55453208	-19,29292551
Crematogaster acuta	-43,59183549	-19,29539139
Crematogaster acuta	-43,59146987	-19,29622689
Crematogaster acuta	-43,5929424	-19,29714278
Crematogaster acuta	-43,58879512	-19,26152489
Crematogaster acuta	-43,58887115	-19,26359874
Crematogaster acuta	-43,58318394	-19,2638813
Crematogaster acuta	-43,54167	-20,375
Crematogaster acuta	-43,50857	-20,43377
Crematogaster acuta	-43,50591	-20,42794
Crematogaster acuta	-43,51466	-20,4262
Crematogaster ampla	-43,50611	-20,4286
Crematogaster arata	-43,5	-20,13333
Crematogaster arcuata	-43,55378919	-19,29447139
Crematogaster arcuata	-43,58845146	-19,26234732
Crematogaster brasiliensis	-43,53812065	-19,26736431
Crematogaster brasiliensis	-43,54259047	-19,25948038
Crematogaster brasiliensis	-43,55219219	-19,24620646
Crematogaster brasiliensis	-43,51328122	-19,21331612
Crematogaster brasiliensis	-43,51561717	-19,22602904
Crematogaster brasiliensis	-43,5170695	-19,25526789
Crematogaster brasiliensis	-43,51693237	-19,25473991
Crematogaster brasiliensis	-43,60643718	-19,26963774
Crematogaster brasiliensis	-43,55569591	-19,29324931
Crematogaster brasiliensis	-43,58776398	-19,26282191
Crematogaster brasiliensis	-43,58887115	-19,26359874
Crematogaster brasiliensis	-43,51284	-19,21324
Crematogaster brasiliensis	-43,51555	-19,22628
Crematogaster brasiliensis	-43,51695	-19,25501
Crematogaster brasiliensis	-43,54231	-19,25955

Crematogaster brasiliensis	-43,5523	-19,24623
Crematogaster brasiliensis	-43,51444	-20,44056
Crematogaster brevispinosa	-44	-18
Crematogaster brevispinosa	-44	-18
Crematogaster brevispinosa	-44	-18
Crematogaster chodati	-43,64324	-19,77012
Crematogaster crinosa	-43,59320551	-19,29679611
Crematogaster crinosa	-43,594	-19,29
Crematogaster curvispinosa	-44	-18
Crematogaster curvispinosa	-43,68333	-20,51667
Crematogaster erecta	-43,50768546	-19,21746248
Crematogaster erecta	-43,51099782	-19,21516165
Crematogaster erecta	-43,51326537	-19,2131401
Crematogaster erecta	-43,51276833	-19,23850274
Crematogaster erecta	-43,616667	-19,266667
Crematogaster erecta	-43,50796	-19,21721
Crematogaster erecta	-43,51081	-19,20915
Crematogaster erecta	-43,51284	-19,21324
Crematogaster erecta	-43,51269	-19,2387
Crematogaster erecta	-44,01166	-20,05977
Crematogaster erecta	-43,51444	-20,44056
Crematogaster erecta	-43,594	-19,29
Crematogaster erecta	-43,51477	-20,42596
Crematogaster erecta	-43,50857	-20,3377
Crematogaster evallans	-43,87111111	-20,02111111
Crematogaster evallans	-43,89027778	-20,06416667
Crematogaster evallans	-43,50667	-20,4286
Crematogaster fuliginea	-44	-18
Crematogaster goeldi	-43,51477	-20,42596
Crematogaster goeldi	-43,50857	-20,3377
Crematogaster goeldi	-43,50562	-20,4278
Crematogaster goeldii	-43,54167	-20,375
Crematogaster goeldii	-44,02138	-20,05955
Crematogaster goeldii	-43,50856	-20,43384
Crematogaster goeldii	-43,50878	-20,43483
Crematogaster goeldii	-43,50896	-20,43476
Crematogaster goeldii	-43,51429	-20,4262
Crematogaster goeldii	-43,50591	-20,42794
Crematogaster indet	-43,5149	-20,4263
Crematogaster lutzi	-43,5	-20,13333
Crematogaster magnifica	-44	-18
Crematogaster moelleri	-44,01166	-20,05977
Crematogaster nitidiceps	-43,5	-20,13333
Crematogaster obscurata	-43,55378919	-19,29447139
Crematogaster obscurata	-43,59165218	-19,29582565
Crematogaster quadriformis	-43,616667	-19,266667
Crematogaster quadriformis	-43,6	-18,25
Crematogaster quadriformis	-43,54167	-20,375

Crematogaster quadriformis	-43,50585	-20,42815
Crematogaster quadriformis	-43,50857	-20,43377
Crematogaster quadriformis	-43,51446	-20,42615
Crematogaster rochai	-44	-18
Crematogaster sericea	-43,88194444	-21,67888889
Crematogaster sericea	-43,88194	-21,67889
Crematogaster sericea	-43,51444	-20,44056
Crematogaster stigmatica	-44	-18
Crematogaster subtonsa	-44	-18
Crematogaster torosa	-43,55257985	-19,24608199
Crematogaster torosa	-43,55382892	-19,24401468
Crematogaster torosa	-43,5523	-19,24623
Crematogaster torosa	-43,55396	-19,24394
Crematogaster torosa	-43,54167	-20,375
Crematogaster torosa	-43,50888	-20,43446
Crematogaster torosa	-43,50857	-20,3377
Crematogaster torosa	-44	-18
Crematogaster tortosa	-43,51458	-20,42612
Crematogaster tortosa	-43,50562	-20,42787
Crematogaster tortosa	-43,50865	-20,43487
Crematogaster unciata	-44	-18
Cyphomyrmex auritus	-44	-18
Cyphomyrmex dixus	-43,54212686	-19,25955221
Cyphomyrmex dixus	-43,55078621	-19,24817931
Cyphomyrmex dixus	-43,50870855	-19,19974214
Cyphomyrmex dixus	-43,50784513	-19,21756575
Cyphomyrmex dixus	-43,51104224	-19,21521471
Cyphomyrmex dixus	-43,51566595	-19,22640212
Cyphomyrmex dixus	-43,55193109	-19,25301164
Cyphomyrmex dixus	-43,55556205	-19,24489209
Cyphomyrmex dixus	-43,50796	-19,21721
Cyphomyrmex dixus	-43,51081	-19,20915
Cyphomyrmex dixus	-43,51555	-19,22628
Cyphomyrmex dixus	-43,55207	-19,25299
Cyphomyrmex dixus	-43,55573	-19,2447
Cyphomyrmex dixus	-43,51695	-19,25501
Cyphomyrmex dixus	-43,54231	-19,25955
Cyphomyrmex dixus	-43,55089	-19,24794
Cyphomyrmex dixus	-43,50877	-19,1995
Cyphomyrmex hamulatus	-43,54217766	-19,25934853
Cyphomyrmex hamulatus	-43,55204844	-19,2528342
Cyphomyrmex hamulatus	-43,55257985	-19,24608199
Cyphomyrmex hamulatus	-43,50802568	-19,21711237
Cyphomyrmex hamulatus	-43,51283647	-19,21323683
Cyphomyrmex hamulatus	-43,5170695	-19,25526789
Cyphomyrmex hamulatus	-43,50796	-19,21721
Cyphomyrmex hamulatus	-43,51284	-19,21324
Cyphomyrmex hamulatus	-43,55207	-19,25299

Cyphomyrmex hamulatus	-43,51695	-19,25501
Cyphomyrmex hamulatus	-43,54231	-19,25955
Cyphomyrmex hamulatus	-43,5523	-19,24623
Cyphomyrmex indet	-43,5065	-20,4267
Cyphomyrmex minutus	-43,51269	-19,2387
Cyphomyrmex minutus	-43,54231	-19,25955
Cyphomyrmex minutus	-43,68333	-20,51667
Cyphomyrmex peltatus	-41,66667	-12,73333
Cyphomyrmex rimosus	-44	-18
Cyphomyrmex rimosus	-43,50857	-20,43377
Cyphomyrmex salvini	-43,50667	-20,4286
Cyphomyrmex salvini	-43,50611	-20,4286
Cyphomyrmex transversus	-44	-18
Cyphomyrmex transversus	-40,33972	-14,60186
Cyphomyrmex tranversus	-40,43279	-13,43588
Diaphoromyrma sofiae	-41,8	-12,81667
Diaphoromyrma sofiae	-41,8	-12,81667
Dinoponera australis	-44	-18
Dinoponera gigantea	-44	-18
Dinoponera grandis australis	-44	-18
Dinoponera lucida	-44	-18
Dinoponera quadriceps	-42,665444	-13,854411
Discothyrea sexarticulata	-43,553345	-19,25456
Discothyrea sexarticulata	-43,551598	-19,253029
Discothyrea sexarticulata	-43,55179	-19,252981
Discothyrea sexarticulata	-43,55172	-19,252962
Discothyrea sexarticulata	-43,51552639	-19,22649834
Discothyrea sexarticulata	-40,33972	-14,60186
Dolichoderus attelaboides	-44	-18
Dolichoderus bispinosus	-43,51283647	-19,21323683
Dolichoderus bispinosus	-43,616667	-19,266667
Dolichoderus lutosus	-41,66667	-12,73333
Dorymyrmex brunneus	-43,53848334	-19,26651581
Dorymyrmex brunneus	-43,53398041	-19,26513791
Dorymyrmex brunneus	-43,57297277	-19,22959956
Dorymyrmex brunneus	-43,57610467	-19,23012905
Dorymyrmex brunneus	-43,51099782	-19,21516165
Dorymyrmex brunneus	-43,60600694	-19,2747227
Dorymyrmex brunneus	-43,60558407	-19,27022221
Dorymyrmex brunneus	-43,60916399	-19,35970422
Dorymyrmex brunneus	-43,55659034	-19,29551871
Dorymyrmex brunneus	-43,59146987	-19,29622689
Dorymyrmex brunneus	-43,59493964	-19,29602548
Dorymyrmex brunneus	-43,58924758	-19,26275661
Dorymyrmex brunneus	-43,58354487	-19,2630799
Dorymyrmex brunneus	-43,89027778	-20,06416667
Dorymyrmex brunneus	-43,51081	-19,20915
Dorymyrmex brunneus	-43,5	-20,4

Dorymyrmex goeldii	-43,53848334	-19,26651581
Dorymyrmex goeldii	-43,53398041	-19,26513791
Dorymyrmex goeldii	-43,57703799	-19,23056072
Dorymyrmex goeldii	-43,60516305	-19,26909995
Dorymyrmex goeldii	-43,60916399	-19,35970422
Dorymyrmex goeldii	-43,55378919	-19,29447139
Dorymyrmex goeldii	-43,59183549	-19,29539139
Dorymyrmex goeldii	-43,5913195	-19,29668278
Dorymyrmex goeldii	-43,59320551	-19,29679611
Dorymyrmex goeldii	-43,58906569	-19,26318266
Dorymyrmex goeldii	-43,58862606	-19,26193929
Dorymyrmex goeldii	-43,58338041	-19,2635513
Dorymyrmex goeldii	-43,61667	-19,36668
Dorymyrmex goeldii	-43,594	-19,29
Dorymyrmex pyramicos	-43,57689064	-19,23097428
Dorymyrmex pyramicos	-43,57590409	-19,23054487
Dorymyrmex pyramicos	-43,60535257	-19,26870232
Dorymyrmex pyramicos	-43,60935476	-19,35928596
Dorymyrmex pyramicos	-43,55417476	-19,29365398
Dorymyrmex pyramicos	-43,59419558	-19,29766774
Dorymyrmex pyramicos	-43,58887115	-19,26359874
Dorymyrmex pyramicos	-43,58354487	-19,2630799
Dorymyrmex pyramicus	-43,53637193	-19,26544771
Dorymyrmex pyramicus	-43,53398041	-19,26513791
Dorymyrmex pyramicus	-43,57644095	-19,23024799
Dorymyrmex pyramicus	-43,57590409	-19,23054487
Dorymyrmex pyramicus	-43,60664757	-19,26921252
Dorymyrmex pyramicus	-43,60935476	-19,35928596
Dorymyrmex pyramicus	-43,55453208	-19,29292551
Dorymyrmex pyramicus	-43,59165218	-19,29582565
Dorymyrmex pyramicus	-43,59493964	-19,29602548
Dorymyrmex pyramicus	-43,58792231	-19,29276416
Dorymyrmex pyramicus	-43,58845146	-19,26234732
Dorymyrmex pyramicus	-43,58354487	-19,2630799
Dorymyrmex spurius	-43,89027778	-20,06416667
Dorymyrmez goeldi	-43,57703799	-19,23056072
Dorymyrmez goeldi	-43,57610467	-19,23012905
Dorymyrmez goeldi	-43,60610191	-19,27049839
Dorymyrmez goeldi	-43,60954595	-19,35895404
Dorymyrmez goeldi	-43,55453208	-19,29292551
Dorymyrmez goeldi	-43,59419558	-19,29766774
Dorymyrmez goeldi	-43,58821459	-19,26322608
Dorymyrmez goeldi	-43,58879512	-19,26152489
Dorymyrmez goeldi	-43,58318394	-19,2638813
Eciton burchelli	-44	-18
Eciton burchellii	-43,786651	-20,029328
Eciton coecum	-44	-18
Eciton crassicorne	-44	-18

Eciton diana	-44	-18
Eciton guerini	-44	-18
Eciton illigeri	-44	-18
Eciton mexicanum	-43,60634255	-19,27007644
Eciton mexicanum	-44	-18
Eciton pilosum	-44	-18
Eciton praedator	-44	-18
Eciton quadriglume	-43,46	-20,11
Eciton quadriglume	-43,484781	-20,096421
Eciton quadriglume	-44	-18
Eciton quadriglumem	-43,50667	-20,4286
Eciton quadriglumem	-43,50611	-20,4286
Eciton quadriglumi	-44	-18
Eciton reichenspergeri	-44	-18
Eciton spinolae	-44	-18
Eciton vagans	-43,60592564	-19,26937329
Eciton vagans	-43,60575238	-19,26979146
Eciton vagans	-43,55516265	-19,29446225
Eciton vagans	-43,5771965	-19,23012117
Eciton vagans	-43,60916399	-19,35970422
Eciton vagans	-44	-18
Eciton vagans	-44	-18
Eciton vagans	-44	-18
Eciton vagans	-43,68333	-20,51667
Ectatomma brunneum	-43,57539447	-19,23100102
Ectatomma brunneum	-43,5771965	-19,23012117
Ectatomma brunneum	-43,60609579	-19,26899141
Ectatomma brunneum	-43,60575238	-19,26979146
Ectatomma brunneum	-43,60916399	-19,35970422
Ectatomma brunneum	-43,59146987	-19,29622689
Ectatomma brunneum	-43,586907	-19,352594
Ectatomma brunneum	-43,616667	-19,266667
Ectatomma brunneum	-41,44295	-11,96887
Ectatomma brunneum	-40,43279	-13,43588
Ectatomma brunneum	-41,66667	-12,73333
Ectatomma edentatum	-43,53673093	-19,26461413
Ectatomma edentatum	-43,5752322	-19,22795009
Ectatomma edentatum	-43,57665964	-19,22886573
Ectatomma edentatum	-43,60554166	-19,26825246
Ectatomma edentatum	-43,60477941	-19,26995122
Ectatomma edentatum	-43,60954595	-19,35895404
Ectatomma edentatum	-43,55516265	-19,29446225
Ectatomma edentatum	-43,55709803	-19,29470834
Ectatomma edentatum	-43,59165218	-19,29582565
Ectatomma edentatum	-43,5935055	-19,29643543
Ectatomma edentatum	-43,58792231	-19,29276416
Ectatomma edentatum	-43,58781084	-19,26253382
Ectatomma edentatum	-43,88194444	-21,67888889

Ectatomma edentatum	-43,616667	-19,266667
Ectatomma edentatum	-41,44295	-11,96887
Ectatomma edentatum	-43,95175	-20,09436
Ectatomma edentatum	-43,94906	-20,03067
Ectatomma edentatum	-43,94403	-20,10136
Ectatomma edentatum	-43,96932	-20,05064
Ectatomma edentatum	-43,95039	-20,02503
Ectatomma edentatum	-43,96778	-20,08602
Ectatomma edentatum	-43,95	-20,05
Ectatomma edentatum	-40,4308	-13,4411
Ectatomma edentatum	-44	-18
Ectatomma edentatum	-43,88333	-21,7
Ectatomma edentatum	-43,88194	-21,67889
Ectatomma edentatum	-43,45882	-20,23164
Ectatomma edentatum	-43,49673	-20,2269
Ectatomma edentatum	-43,50576	-20,21305
Ectatomma edentatum	-43,50718	-20,19246
Ectatomma edentatum	-43,5147	-20,18679
Ectatomma edentatum	-43,594	-19,29
Ectatomma edentatum	-43,5497	-20,4731
Ectatomma edentatum	-43,4881	-20,0981
Ectatomma edentatum	-43,50857	-20,3377
Ectatomma lugens	-41,44295	-11,96887
Ectatomma muticum	-43,5	-19,16666667
Ectatomma muticum	-41,44295	-11,96887
Ectatomma muticum	-43,5	-19,16667
Ectatomma muticum	-40,85	-14,85
Ectatomma opaciventre	-43,60575238	-19,26979146
Ectatomma opaciventre	-43,60954595	-19,35895404
Ectatomma opaciventre	-43,58906569	-19,26318266
Ectatomma opaciventre	-43,4875	-18,414752
Ectatomma opaciventre	-43,586907	-19,352594
Ectatomma opaciventre	-43,49135	-20,11722
Ectatomma opaciventre	-44	-18
Ectatomma opaciventre	-43,44961	-20,08184
Ectatomma permagnum	-43,5768541	-19,22937987
Ectatomma permagnum	-43,60682149	-19,26881321
Ectatomma permagnum	-43,60935476	-19,35928596
Ectatomma permagnum	-43,5891076	-19,29561845
Ectatomma permagnum	-43,616667	-19,266667
Ectatomma permagnum	-43,95278	-20,02861
Ectatomma permagnum	-44	-18
Ectatomma permagnum	-43,68333	-20,51667
Ectatomma permagnum	-43,44961	-20,08184
Ectatomma planidens	-43,53884024	-19,26565348
Ectatomma planidens	-43,60954595	-19,35895404
Ectatomma planidens	-43,60916399	-19,35970422
Ectatomma planidens	-43,58944908	-19,26236669

Ectatomma planidens	-43,58906569	-19,26318266
Ectatomma planidens	-43,58845146	-19,26234732
Ectatomma planidens	-43,6173	-19,3544
Ectatomma planidens	-44	-18
Ectatomma planidens	-43,65	-19
Ectatomma quadridens	-44	-18
Ectatomma quadridens	-40,4308	-13,4411
Ectatomma tuberculatum	-43,57644095	-19,23024799
Ectatomma tuberculatum	-43,60516305	-19,26909995
Ectatomma tuberculatum	-43,60954595	-19,35895404
Ectatomma tuberculatum	-41,44295	-11,96887
Ectatomma tuberculatum	-40,43279	-13,43588
Ectatomma tuberculatum	-44	-18
Ectatomma tuberculatum	-43,594	-19,29
Ectatomma tuberculatum	-40,4308	-13,4411
Ectatomma vizottoi	-41,66667	-12,73333
Ephebomyrmex abdominalis	-44	-18
Ephebomyrmex naegelii	-44	-18
Euponera marginata	-44	-18
Eurhopalothrix spectabilis	-43,553345	-19,25456
Eurhopalothrix spectabilis	-43,55261	-19,253231
Eurhopalothrix spectabilis	-43,55335	-19,25456
Forelius brasiliensis	-43,57761668	-19,22925783
Forelius brasiliensis	-43,60610191	-19,27049839
Forelius brasiliensis	-43,60935476	-19,35928596
Forelius brasiliensis	-43,59407036	-19,2956923
Forelius brasiliensis	-43,58944908	-19,26236669
Forelius brasiliensis	-43,58879512	-19,26152489
Forelius brasiliensis	-43,58318394	-19,2638813
Forelius maranhaoensis	-43,53398041	-19,26513791
Forelius maranhaoensis	-43,5774275	-19,22970358
Forelius maranhaoensis	-43,57610467	-19,23012905
Forelius maranhaoensis	-43,60516305	-19,26909995
Forelius maranhaoensis	-43,60916399	-19,35970422
Forelius maranhaoensis	-43,59419558	-19,29766774
Forelius maranhaoensis	-43,58713341	-19,29322399
Forelius maranhaoensis	-43,58887115	-19,26359874
Forelius maranhaoensis	-43,58879512	-19,26152489
Forelius maranhaoensis	-43,58338041	-19,2635513
Fulakora armigera	-43,60634339	-19,26858036
Gnamptogenys arcuata	-44	-18
Gnamptogenys caelata	-43,9408	-20,0881
Gnamptogenys caelata	-43,9408	-20,0881
Gnamptogenys fiebrigi	-44	-18
Gnamptogenys lavra	-44	-18
Gnamptogenys lucaris	-44	-18
Gnamptogenys pleurodon	-40,33972	-14,60186
Gnamptogenys pleurodon	-43,50611	-20,4286

Gnamptogenys regularis	-44	-18
Gnamptogenys reichenspergeri	-43,50611	-20,4286
Gnamptogenys simplicoides	-44	-18
Gnamptogenys striatula	-43,54217766	-19,25934853
Gnamptogenys striatula	-43,5526713	-19,2462011
Gnamptogenys striatula	-43,5768541	-19,22937987
Gnamptogenys striatula	-43,50898289	-19,19937015
Gnamptogenys striatula	-43,50802568	-19,21711237
Gnamptogenys striatula	-43,51038812	-19,21493199
Gnamptogenys striatula	-43,51328122	-19,21331612
Gnamptogenys striatula	-43,5154595	-19,22641461
Gnamptogenys striatula	-43,54058585	-19,23923389
Gnamptogenys striatula	-43,55236134	-19,25312371
Gnamptogenys striatula	-43,55556205	-19,24489209
Gnamptogenys striatula	-43,51249022	-19,23886878
Gnamptogenys striatula	-43,5171208	-19,25477696
Gnamptogenys striatula	-43,62044871	-19,36814563
Gnamptogenys striatula	-43,62138044	-19,36499219
Gnamptogenys striatula	-43,50796	-19,21721
Gnamptogenys striatula	-43,51081	-19,20915
Gnamptogenys striatula	-43,51555	-19,22628
Gnamptogenys striatula	-43,54059	-19,23923
Gnamptogenys striatula	-43,55207	-19,25299
Gnamptogenys striatula	-43,55573	-19,2447
Gnamptogenys striatula	-43,51269	-19,2387
Gnamptogenys striatula	-43,51695	-19,25501
Gnamptogenys striatula	-43,54231	-19,25955
Gnamptogenys striatula	-43,50877	-19,1995
Gnamptogenys striatula	-44	-18
Gnamptogenys striatula	-43,5	-20,13333
Gnamptogenys striatula	-43,45882	-20,23164
Gnamptogenys striatula	-43,50599	-20,19277
Gnamptogenys striatula	-43,51444	-20,44055
Gnamptogenys striatula	-43,86667	-21,66667
Gnamptogenys striatula	-43,50562	-20,4278
Gnamptogenys striatula	-43,50857	-20,3377
Gnamptogenys striatula	-43,51477	-20,42596
Gnamptogenys sulcata	-43,53418258	-19,26471345
Gnamptogenys sulcata	-43,55093289	-19,24823379
Gnamptogenys sulcata	-43,57628714	-19,23067245
Gnamptogenys sulcata	-43,57590409	-19,23054487
Gnamptogenys sulcata	-43,60609579	-19,26899141
Gnamptogenys sulcata	-43,60935476	-19,35928596
Gnamptogenys sulcata	-43,55453208	-19,29292551
Gnamptogenys sulcata	-43,59380012	-19,29607602
Gnamptogenys sulcata	-43,58674466	-19,29347931
Gnamptogenys sulcata	-43,58845146	-19,26234732
Gnamptogenys sulcata	-43,58879512	-19,26152489

Gnamptogenys sulcata	-43,55089	-19,24794
Gnamptogenys sulcata	-43,5	-20,4
Gnamptogenys sulcata	-43,68333	-20,51667
Gnamptogenys sulcata	-43,50857	-20,3377
Heteroponera angulata	-40,33972	-14,60186
Heteroponera dentinodis	-43,88194444	-21,67888889
Heteroponera dentinodis	-43,88194	-21,67889
Heteroponera dentinodis	-43,5	-20,13333
Heteroponera dolo	-44	-18
Heteroponera dolo	-43,5	-20,13333
Heteroponera flava	-43,50857	-20,43377
Heteroponera inemis	-43,88194	-21,67889
Heteroponera inermis	-43,88194444	-21,67888889
Heteroponera inermis	-43,88194	-21,67889
Heteroponera mayri	-40,33717	-14,61117
Heteroponera mayri	-43,86667	-21,66667
Heteroponera mayri	-43,50562	-20,4278
Heteroponera mayri	-43,50667	-20,4286
Heteroponera mayri	-43,50611	-20,4286
Hylomyrma balzani	-43,514927	-20,426323
Hylomyrma balzani	-43,55382892	-19,24401468
Hylomyrma balzani	-43,5744194	-19,22840908
Hylomyrma balzani	-43,508628	-19,19963803
Hylomyrma balzani	-43,50712312	-19,21733357
Hylomyrma balzani	-43,51038812	-19,21493199
Hylomyrma balzani	-43,51543218	-19,22601705
Hylomyrma balzani	-43,55571779	-19,24498463
Hylomyrma balzani	-43,51249022	-19,23886878
Hylomyrma balzani	-43,51693237	-19,25473991
Hylomyrma balzani	-43,54058585	-19,23923389
Hylomyrma balzani	-43,62007379	-19,36781379
Hylomyrma balzani	-43,6212258	-19,36546158
Hylomyrma balzani	-43,50796	-19,21721
Hylomyrma balzani	-43,51081	-19,20915
Hylomyrma balzani	-43,51555	-19,22628
Hylomyrma balzani	-43,55396	-19,24394
Hylomyrma balzani	-43,51269	-19,2387
Hylomyrma balzani	-43,51695	-19,25501
Hylomyrma balzani	-43,50877	-19,1995
Hylomyrma balzani	-40,43247	-13,43334
Hylomyrma balzani	-43,58153	-19,22611
Hylomyrma balzani	-43,55253	-19,25331
Hylomyrma balzani	-43,5149	-20,4263
Hylomyrma balzani	-43,50667	-20,4286
Hylomyrma balzani	-43,50611	-20,4286
Hylomyrma primavesi	-43,53472	-19,26389
Hylomyrma primavesi	-43,53444	-19,26389
Hylomyrma reitteri	-43,5386581	-19,26607518

Hylomyrma reitteri	-43,53418258	-19,26471345
Hylomyrma reitteri	-43,53398041	-19,26513791
Hylomyrma reitteri	-43,57644095	-19,23024799
Hylomyrma reitteri	-43,51038812	-19,21493199
Hylomyrma reitteri	-43,62065004	-19,36671224
Hylomyrma reitteri	-43,58790983	-19,29397836
Hylomyrma reitteri	-43,58713341	-19,29322399
Hylomyrma reitteri	-43,55096064	-19,24777488
Hylomyrma reitteri	-43,50870855	-19,19974214
Hylomyrma reitteri	-43,51566595	-19,22640212
Hylomyrma reitteri	-43,55586841	-19,24453829
Hylomyrma reitteri	-43,5171208	-19,25477696
Hylomyrma reitteri	-43,51686758	-19,25521592
Hylomyrma reitteri	-43,51555	-19,22628
Hylomyrma reitteri	-43,55573	-19,2447
Hylomyrma reitteri	-43,51695	-19,25501
Hylomyrma reitteri	-43,55089	-19,24794
Hylomyrma reitteri	-43,50877	-19,1995
Hylomyrma reitteri	-43,86667	-21,66667
Hypoclinea leuderwaldti	-44	-18
Hypoclinea luederwaldti	-44	-18
Hypoclinea lutosa	-44	-18
Hypoponera aliena	-44	-18
Hypoponera collegiana	-44	-18
Hypoponera distinguenda	-43,5744194	-19,22840908
Hypoponera distinguenda	-43,60725383	-19,26506046
Hypoponera distinguenda	-43,60600694	-19,2747227
Hypoponera distinguenda	-43,51552639	-19,22649834
Hypoponera distinguenda	-43,54058585	-19,23923389
Hypoponera distinguenda	-43,62065004	-19,36671224
Hypoponera distinguenda	-43,62102337	-19,36582779
Hypoponera distinguenda	-43,55531571	-19,29406738
Hypoponera distinguenda	-43,59165218	-19,29582565
Hypoponera distinguenda	-43,58777488	-19,2630908
Hypoponera distinguenda	-43,58718286	-19,26190484
Hypoponera distinguenda	-43,50562	-20,4278
Hypoponera distinguenda	-43,51477	-20,42596
Hypoponera foreli	-43,88194444	-21,67888889
Hypoponera foreli	-44	-18
Hypoponera foreli	-43,88194	-21,67889
Hypoponera foreli	-40,33972	-14,60186
Hypoponera foreli	-43,50667	-20,4286
Hypoponera foreli	-43,50562	-20,4278
Hypoponera iheringi	-43,44961	-20,08184
Hypoponera jheringi	-43,5	-20,13333
Hypoponera opaciceps	-43,44961	-20,08184
Hypoponera parva	-43,44961	-20,08184
Hypoponera schmalzi	-43,44961	-20,08184

Hypoponera trigona	-43,44961	-20,08184
Iridomyrmex leucomelas	-44	-18
Iridomyrmex leucomelas	-43,5	-20,13333
Kalathomyrmex emeryi	-43,57610467	-19,23012905
Kalathomyrmex emeryi	-43,60610191	-19,27049839
Kalathomyrmex emeryi	-43,60916399	-19,35970422
Kalathomyrmex emeryi	-43,55552307	-19,29367712
Kalathomyrmex emeryi	-43,59419558	-19,29766774
Kalathomyrmex emeryi	-43,58713341	-19,29322399
Kalathomyrmex emeryi	-43,58906569	-19,26318266
Kalathomyrmex emeryi	-43,88333	-21,7
Labidus coecus	-43,53398041	-19,26513791
Labidus coecus	-43,5771965	-19,23012117
Labidus coecus	-43,62102337	-19,36582779
Labidus coecus	-43,6212258	-19,36546158
Labidus coecus	-43,62138044	-19,36499219
Labidus coecus	-43,55681053	-19,29509978
Labidus coecus	-44	-18
Labidus nero	-44	-18
Labidus praedator	-41,458002	-12,781015
Labidus praedator	-43,948856	-20,176769
Labidus praedator	-44,009605	-20,052099
Labidus praedator	-43,50870855	-19,19974214
Labidus praedator	-43,55193109	-19,25301164
Labidus praedator	-43,60916399	-19,35970422
Labidus praedator	-43,55725486	-19,29432621
Labidus praedator	-43,58824267	-19,26276759
Labidus praedator	-43,616667	-19,266667
Labidus praedator	-43,50972	-20,43833
Labidus praedator	-43,55207	-19,25299
Labidus praedator	-43,50877	-19,1995
Labidus praedator	-44	-18
Labidus praedator	-43,45882	-20,23164
Labidus praedator	-43,68333	-20,51667
Labidus praedator	-43,50611	-20,4286
Labidus spininodis	-43,60634339	-19,26858036
Lachnomyrmex victori	-43,514927	-20,426323
Lachnomyrmex victori	-43,5149	-20,4263
Leptogenys crudelis	-43,61930802	-19,3672191
Leptogenys gorgona	-43,61930802	-19,3672191
Leptogenys iheringi	-43,50667	-20,4286
Leptogenys iheringi	-43,50611	-20,4286
Leptogenys luederwaldti	-43,86667	-21,66667
Leptothorax silvestrii	-44	-18
Linepithema aztecoides	-43,53830405	-19,26693684
Linepithema aztecoides	-43,57356537	-19,22774909
Linepithema aztecoides	-43,57360393	-19,23045502
Linepithema aztecoides	-43,5771965	-19,23012117

Linepithema aztecoides	-43,57650792	-19,22930259
Linepithema aztecoides	-43,55412891	-19,24364579
Linepithema aztecoides	-43,50768546	-19,21746248
Linepithema aztecoides	-43,5402354	-19,23933799
Linepithema aztecoides	-43,60723883	-19,26576605
Linepithema aztecoides	-43,60634339	-19,26858036
Linepithema aztecoides	-43,55681053	-19,29509978
Linepithema aztecoides	-43,5935055	-19,29643543
Linepithema aztecoides	-43,59419558	-19,29766774
Linepithema aztecoides	-43,5539166	-19,24407754
Linepithema aztecoides	-43,50796	-19,21721
Linepithema aztecoides	-43,51081	-19,20915
Linepithema aztecoides	-43,51284	-19,21324
Linepithema aztecoides	-43,54059	-19,23923
Linepithema aztecoides	-43,55396	-19,24394
Linepithema cerradense	-43,88194444	-21,67888889
Linepithema cerradense	-43,53398041	-19,26513791
Linepithema cerradense	-43,5774275	-19,22970358
Linepithema cerradense	-43,50768546	-19,21746248
Linepithema cerradense	-43,60575238	-19,26979146
Linepithema cerradense	-43,60634339	-19,26858036
Linepithema cerradense	-43,5539938	-19,29405481
Linepithema cerradense	-43,55709803	-19,29470834
Linepithema cerradense	-43,59165218	-19,29582565
Linepithema cerradense	-43,5891076	-19,29561845
Linepithema cerradense	-43,58869806	-19,26403485
Linepithema cerradense	-43,88333	-21,7
Linepithema cerradense	-43,88194	-21,67889
Linepithema gallardoi	-43,57379302	-19,2279412
Linepithema gallardoi	-43,5	-20,1333
Linepithema gallardoi	-43,5	-20,13333
Linepithema gallardoi	-43,44961	-20,08184
Linepithema humile	-43,54166667	-20,375
Linepithema humile	-43,616667	-19,266667
Linepithema humile	-43,54167	-20,375
Linepithema iniquum	-43,50802568	-19,21711237
Linepithema iniquum	-43,53673093	-19,26461413
Linepithema iniquum	-43,54217766	-19,25934853
Linepithema iniquum	-43,55096064	-19,24777488
Linepithema iniquum	-43,5752322	-19,22795009
Linepithema iniquum	-43,50870855	-19,19974214
Linepithema iniquum	-43,50768546	-19,21746248
Linepithema iniquum	-43,51099782	-19,21516165
Linepithema iniquum	-43,55193109	-19,25301164
Linepithema iniquum	-43,55571779	-19,24498463
Linepithema iniquum	-43,51686758	-19,25521592
Linepithema iniquum	-43,58845146	-19,26234732
Linepithema iniquum	-43,5	-20,1333

Linepithema iniquum	-43,50796	-19,21721
Linepithema iniquum	-43,51081	-19,20915
Linepithema iniquum	-43,55089	-19,24794
Linepithema iniquum	-43,55573	-19,2447
Linepithema iniquum	-43,51695	-19,25501
Linepithema iniquum	-43,54231	-19,25955
Linepithema iniquum	-43,55396	-19,24394
Linepithema iniquum	-43,50877	-19,1995
Linepithema iniquum	-40,43292	-13,43694
Linepithema iniquum	-43,45882	-20,23164
Linepithema iniquum	-43,54167	-20,375
Linepithema iniquum	-40,4308	-13,4411
Linepithema iniquum	-43,44961	-20,08184
Linepithema iniquum	-43,50536	-20,42768
Linepithema iniquum	-43,50857	-20,43377
Linepithema iniquum	-43,51429	-20,4262
Linepithema leucomelas	-43,8833	-21,7
Linepithema leucomelas	-43,5774275	-19,22970358
Linepithema leucomelas	-43,50898289	-19,19937015
Linepithema leucomelas	-43,50784513	-19,21756575
Linepithema leucomelas	-43,51283647	-19,21323683
Linepithema leucomelas	-43,51566595	-19,22640212
Linepithema leucomelas	-43,5556887	-19,24451843
Linepithema leucomelas	-43,60497655	-19,26951637
Linepithema leucomelas	-43,58869806	-19,26403485
Linepithema leucomelas	-43,8833	-21,7
Linepithema leucomelas	-43,5	-20,13333
Linepithema leucomelas	-43,50796	-19,21721
Linepithema leucomelas	-43,51284	-19,21324
Linepithema leucomelas	-43,51555	-19,22628
Linepithema leucomelas	-43,55573	-19,2447
Linepithema leucomelas	-43,50877	-19,1995
Linepithema leucomelas	-43,45882	-20,23164
Linepithema leucomelas	-43,50576	-20,21305
Linepithema leucomelas	-43,50581	-20,19359
Linepithema leucomelas	-43,90292	-21,7118
Linepithema leucomelas	-43,44961	-20,08184
Linepithema micans	-43,5	-20,1333
Linepithema micans	-43,5359986	-19,26630769
Linepithema micans	-43,53830405	-19,26693684
Linepithema micans	-43,53379182	-19,26554787
Linepithema micans	-43,5774275	-19,22970358
Linepithema micans	-43,57590409	-19,23054487
Linepithema micans	-43,50870855	-19,19974214
Linepithema micans	-43,50784513	-19,21756575
Linepithema micans	-43,51328122	-19,21331612
Linepithema micans	-43,51239273	-19,23875252
Linepithema micans	-43,5171208	-19,25477696

Linepithema micans	-43,60558407	-19,27022221
Linepithema micans	-43,60935476	-19,35928596
Linepithema micans	-43,60916399	-19,35970422
Linepithema micans	-43,55659034	-19,29551871
Linepithema micans	-43,58971546	-19,29438681
Linepithema micans	-43,5929424	-19,29714278
Linepithema micans	-43,58879512	-19,26152489
Linepithema micans	-43,58338041	-19,2635513
Linepithema micans	-43,50667	-20,4286
Linepithema micans	-43,50611	-20,4286
Linepithema micans	-43,5	-20,38333
Linepithema micans	-43,5	-20,1333
Linepithema micans	-43,50857	-20,43377
Linepithema micans	-43,50796	-19,21721
Linepithema micans	-43,51081	-19,20915
Linepithema micans	-43,51269	-19,2387
Linepithema micans	-43,51695	-19,25501
Linepithema micans	-43,50877	-19,1995
Linepithema micans	-43,54167	-20,375
Linepithema micans	-43,54166	-20,375
Linepithema micans	-43,45882	-20,23164
Linepithema micans	-43,68333	-20,51667
Linepithema micans	-43,594	-19,29
Linepithema micans	-43,44961	-20,08184
Linepithema micans	-43,4881	-20,0981
Linepithema micans	-43,50562	-20,4278
Linepithema micans	-43,50857	-20,3377
Linepithema micans	-43,51477	-20,42596
Linepithema neotropicum	-43,55222211	-19,24632213
Linepithema neotropicum	-43,508628	-19,19963803
Linepithema neotropicum	-43,51561717	-19,22602904
Linepithema neotropicum	-43,53654779	-19,26502635
Linepithema neotropicum	-43,54255216	-19,25967802
Linepithema neotropicum	-43,5771965	-19,23012117
Linepithema neotropicum	-43,5526713	-19,2462011
Linepithema neotropicum	-43,50870855	-19,19974214
Linepithema neotropicum	-43,51054427	-19,21544027
Linepithema neotropicum	-43,54063815	-19,23901387
Linepithema neotropicum	-43,51291836	-19,2386407
Linepithema neotropicum	-43,60554166	-19,26825246
Linepithema neotropicum	-43,60643718	-19,26963774
Linepithema neotropicum	-43,58993556	-19,2939704
Linepithema neotropicum	-43,58971546	-19,29438681
Linepithema neotropicum	-43,58354487	-19,2630799
Linepithema neotropicum	-43,5	-20,1333
Linepithema neotropicum	-43,51081	-19,20915
Linepithema neotropicum	-43,51555	-19,22628
Linepithema neotropicum	-43,54059	-19,23923

Linepithema neotropicum	-43,51269	-19,2387
Linepithema neotropicum	-43,54231	-19,25955
Linepithema neotropicum	-43,5523	-19,24623
Linepithema neotropicum	-43,50877	-19,1995
Linepithema pulex	-43,88194444	-21,67888889
Linepithema pulex	-43,5752322	-19,22795009
Linepithema pulex	-43,57566504	-19,22799083
Linepithema pulex	-43,54058585	-19,23923389
Linepithema pulex	-43,62089907	-19,36628007
Linepithema pulex	-43,58776398	-19,26282191
Linepithema pulex	-43,58846588	-19,26102131
Linepithema pulex	-43,58697667	-19,26169588
Linepithema pulex	-43,55093289	-19,24823379
Linepithema pulex	-43,51067009	-19,21557186
Linepithema pulex	-43,5154595	-19,22641461
Linepithema pulex	-43,51276833	-19,23850274
Linepithema pulex	-43,5170695	-19,25526789
Linepithema pulex	-43,57689064	-19,23097428
Linepithema pulex	-43,508628	-19,19963803
Linepithema pulex	-43,50870855	-19,19974214
Linepithema pulex	-43,50784513	-19,21756575
Linepithema pulex	-43,51099782	-19,21516165
Linepithema pulex	-43,51561717	-19,22602904
Linepithema pulex	-43,5402354	-19,23933799
Linepithema pulex	-43,51239273	-19,23875252
Linepithema pulex	-43,5170695	-19,25526789
Linepithema pulex	-43,51693237	-19,25473991
Linepithema pulex	-43,59201838	-19,29497782
Linepithema pulex	-43,5913195	-19,29668278
Linepithema pulex	-43,58824267	-19,26276759
Linepithema pulex	-43,50796	-19,21721
Linepithema pulex	-43,51081	-19,20915
Linepithema pulex	-43,51555	-19,22628
Linepithema pulex	-43,54059	-19,23923
Linepithema pulex	-43,51269	-19,2387
Linepithema pulex	-43,51695	-19,25501
Linepithema pulex	-43,55396	-19,24394
Linepithema pulex	-43,50877	-19,1995
Linepithema pulex	-43,88194	-21,67889
Linepithema pulex	-43,45882	-20,23164
Linepithema pulex	-43,50718	-20,19246
Linepithema pulex	-43,5147	-20,18679
Linepithema pulex	-43,50599	-20,19277
Megalomyrmex goeldii	-44	-18
Megalomyrmex goeldii	-40,33972	-14,60186
Megalomyrmex goeldii	-43,50562	-20,4278
Megalomyrmex goeldii	-43,50857	-20,43377
Megalomyrmex goeldii	-43,51477	-20,42596

Megalomyrmex leoninus	-43,45882	-20,23164
Megalomyrmex pusillus	-40,33972	-14,60186
Monacis bispinosa	-44	-18
Monomorium pharaonis	-44	-18
Mycetagroicus cerradensis	-44	-18
Mycetagroicus triangularis	-44	-18
Mycetarotes carinatus	-44	-18
Mycetarotes parallelus	-44	-18
Mycetarotes parallelus	-43,68333	-20,51667
Mycetomoellerius holmgreni	-43,67244	-20,34953
Mycetomoellerius urichii	-41,5741	-12,61725
Mycetophylax lectus	-43,57689064	-19,23097428
Mycetophylax lectus	-43,60554166	-19,26825246
Mycetophylax lectus	-43,60954595	-19,35895404
Mycetophylax lectus	-43,55417476	-19,29365398
Mycetophylax lectus	-43,59146987	-19,29622689
Mycetophylax lectus	-43,5935055	-19,29643543
Mycetophylax plaumanni	-40,33972	-14,60186
Mycocepurus goeldii	-43,53673093	-19,26461413
Mycocepurus goeldii	-43,57593703	-19,227891
Mycocepurus goeldii	-43,60592564	-19,26937329
Mycocepurus goeldii	-43,60610191	-19,27049839
Mycocepurus goeldii	-43,5539938	-19,29405481
Mycocepurus goeldii	-43,55709803	-19,29470834
Mycocepurus goeldii	-43,55681053	-19,29509978
Mycocepurus goeldii	-43,59183549	-19,29539139
Mycocepurus goeldii	-43,58374184	-19,2626567
Mycocepurus goeldii	-43,616667	-19,266667
Mycocepurus goeldii	-44	-18
Mycocepurus goeldii	-43,68333	-20,51667
Mycocepurus smithi	-44	-18
Mycocepurus smithii	-43,57628714	-19,23067245
Mycocepurus smithii	-43,57703799	-19,23056072
Mycocepurus smithii	-43,55709803	-19,29470834
Mycocepurus smithii	-44	-18
Myrmelachista bambusarum	-43,505627	-20,398066
Myrmelachista bruchi	-43,54059	-19,23923
Myrmelachista bruchi	-43,55089	-19,24794
Myrmelachista bruchi	-43,55573	-19,2447
Myrmelachista bruchi	-43,54231	-19,25955
Myrmelachista bruchi	-43,54167	-20,375
Myrmelachista catharinae	-40,83889	-14,841111
Myrmelachista catharinae	-43,550888	-19,247944
Myrmelachista catharinae	-43,50611	-20,428055
Myrmelachista catharinae	-43,643238	-19,770119
Myrmelachista catharinae	-43,54259047	-19,25948038
Myrmelachista catharinae	-43,55193109	-19,25301164
Myrmelachista catharinae	-43,57590409	-19,23054487

Myrmelachista catharinae	-43,55222211	-19,24632213
Myrmelachista catharinae	-43,55571779	-19,24498463
Myrmelachista catharinae	-43,54070504	-19,23915074
Myrmelachista catharinae	-43,62007379	-19,36781379
Myrmelachista catharinae	-43,58853185	-19,26043608
Myrmelachista catharinae	-43,5	-20,4
Myrmelachista catharinae	-43,50591	-20,42794
Myrmelachista catharinae	-43,50878	-20,43423
Myrmelachista catharinae	-43,51466	-20,4262
Myrmelachista catharinae	-43,50857	-20,3377
Myrmelachista gagatina	-43,514473	-20,42614
Myrmelachista gagatina	-43,508778	-20,434221
Myrmelachista gagatina	-43,514442	-20,42639
Myrmelachista gagatina	-40,83829	-14,841305
Myrmelachista gagatina	-43,514168	-20,426111
Myrmelachista gagatina	-44	-18
Myrmelachista gallicola	-43,51	-20,41
Myrmelachista gallicola	-43,5045	-20,396
Myrmelachista gallicola	-43,5117	-20,39367
Myrmelachista gallicola	-43,50813	-20,39641
Myrmelachista gallicola	-43,514446	-20,440556
Myrmelachista kloetersi	-43,5	-20,1345
Myrmelachista nodigera	-41,37	-13
Myrmelachista nodigera	-43,616943	-19,3675
Myrmelachista nodigera	-43,60707195	-19,268001
Myrmelachista nodigera	-44	-18
Myrmelachista nodigera	-43,65	-19
Myrmelachista nodigera	-43,61694	-19,3675
Myrmelachista nodigera	-43,51444	-20,44056
Myrmelachista nodigera	-43,5	-20,4
Myrmelachista nodigera	-43,50667	-20,4286
Myrmelachista rudolphi	-43,5	-20,1345
Myrmelachista ruszkii	-43,586907	-19,352594
Myrmelachista ruzskyi	-43,53884024	-19,26565348
Myrmelachista ruzskyi	-43,53379182	-19,26554787
Myrmelachista ruzskyi	-43,5771965	-19,23012117
Myrmelachista ruzskyi	-43,57665964	-19,22886573
Myrmelachista ruzskyi	-43,51261133	-19,21331143
Myrmelachista ruzskyi	-43,59473756	-19,29644893
Myrmelachista ruzskyi	-43,59493964	-19,29602548
Myrmelachista ruzskyi	-43,59419558	-19,29766774
Myrmelachista ruzskyi	-43,58805047	-19,26201842
Neivamyrmex asper	-43,55725486	-19,29432621
Neivamyrmex bohlsi	-44	-18
Neivamyrmex carettei	-44	-18
Neivamyrmex densepunctatum	-44	-18
Neivamyrmex diana	-44	-18
Neivamyrmex guerini	-44	-18

Neivamyrmex halidayi	-44	-18
Neivamyrmex hopei	-44	-18
Neivamyrmex jermanni	-44	-18
Neivamyrmex jerrmanni	-44	-18
Neivamyrmex leptognathus	-43,5	-20,13333
Neivamyrmex leptognathus	-44	-18
Neivamyrmex minensis	-44	-18
Neivamyrmex orthonotus	-44	-18
Neivamyrmex pertyi	-44	-18
Neivamyrmex pilosus	-44	-18
Neivamyrmex pseudops	-43,57630173	-19,22971783
Neivamyrmex pseudops	-43,55709803	-19,29470834
Neivamyrmex pseudops	-43,55433955	-19,29329633
Neivamyrmex pseudops	-43,58944908	-19,26236669
Neivamyrmex pseudops	-43,58338041	-19,2635513
Neivamyrmex pseudops	-44	-18
Neivamyrmex spinolai	-44	-18
Neivamyrmex swainsoni	-44	-18
Neivamyrmex swainsonii	-43,60954595	-19,35895404
Neoponera bactronica	-40,3411	-14,6147
Neoponera carinulata	-44	-18
Neoponera commutata	-44	-18
Neoponera concava	-40,3397	-14,6519
Neoponera crenata	-43,50712312	-19,21733357
Neoponera crenata	-43,51566595	-19,22640212
Neoponera crenata	-43,54070504	-19,23915074
Neoponera crenata	-43,51249022	-19,23886878
Neoponera crenata	-43,58853185	-19,26043608
Neoponera crenata	-43,50796	-19,21721
Neoponera crenata	-43,51284	-19,21324
Neoponera crenata	-43,51555	-19,22628
Neoponera crenata	-43,54059	-19,23923
Neoponera crenata	-43,51269	-19,2387
Neoponera crenata	-44	-18
Neoponera crenata	-43,50667	-20,4286
Neoponera crenata	-43,50857	-20,43377
Neoponera crenata	-44	-18
Neoponera curvinodis	-43,643238	-19,770119
Neoponera latinoda	-43,58853185	-19,26043608
Neoponera magnifica	-40,8389	-14,8411
Neoponera marginata	-43,67244	-20,34953
Neoponera obscuricornis	-44	-18
Neoponera obscuricornis	-43,50562	-20,4278
Neoponera obscuricornis	-43,50857	-20,43377
Neoponera unidentata	-44	-18
Neoponera venusta	-40,33972	-14,60186
Neoponera verenae	-43,643288	-19,770119
Neoponera verenae	-43,616667	-19,266667

Neoponera verenae	-43,68333	-20,51667
Neoponera villosa	-43,54166667	-20,375
Neoponera villosa	-43,60954595	-19,35895404
Neoponera villosa	-43,60916399	-19,35970422
Neoponera villosa	-43,58887115	-19,26359874
Neoponera villosa	-43,616667	-19,266667
Neoponera villosa	-44	-18
Neoponera villosa	-43,65	-19
Neoponera villosa	-43,66	-19,202
Nesomyrmex asper	-43,54167	-20,375
Nesomyrmex asper	-43,50896	-20,43476
Nesomyrmex asper	-43,50878	-20,43416
Nesomyrmex echinatinodis	-43,50667	-20,4286
Nesomyrmex spininodis	-44	-18
Nesomyrmex spininodis	-43,594	-19,29
Nomamyrmex esenbecki	-44	-18
Nomamyrmex esenbecki	-44,26107	-17,82969
Nomamyrmex esenbeckii	-43,5768541	-19,22937987
Nomamyrmex esenbeckii	-43,5771965	-19,23012117
Nomamyrmex esenbeckii	-43,58906569	-19,26318266
Nomamyrmex esenbeckii	-43,58887115	-19,26359874
Nomamyrmex hartigi	-44	-18
Nylanderia fulva	-43,50857	-20,3377
Nylanderia fulva	-43,50562	-20,4278
Nylanderia fulva	-43,51477	-20,42596
Ochetomyrmex neopolitus	-40,33717	-14,64783
Ochetomyrmex semipolitus	-43,57630173	-19,22971783
Ochetomyrmex semipolitus	-43,60516305	-19,26909995
Ochetomyrmex semipolitus	-43,60954595	-19,35895404
Ochetomyrmex semipolitus	-43,55531571	-19,29406738
Ochetomyrmex semipolitus	-43,5929424	-19,29714278
Ochetomyrmex semipolitus	-43,58792231	-19,29276416
Ochetomyrmex semipolitus	-43,58845146	-19,26234732
Ochetomyrmex semipolitus	-43,58906569	-19,26318266
Ochetomyrmex semipolitus	-44	-18
Ochetomyrmex semipolitus	-40,33972	-14,60186
Octostruma balsani	-43,50562	-20,4278
Octostruma balzani	-43,88194444	-21,67888889
Octostruma balzani	-43,88194	-21,67889
Octostruma balzani	-43,50562	-20,4278
Octostruma balzani	-43,50611	-20,4286
Octostruma iheringi	-43,59456489	-19,29685922
Octostruma iheringi	-43,59419558	-19,29766774
Octostruma jheringhi	-41,66667	-12,73333
Octostruma rugifera	-43,88194444	-21,67888889
Octostruma rugifera	-43,50712312	-19,21733357
Octostruma rugifera	-43,51038812	-19,21493199
Octostruma rugifera	-43,51261133	-19,21331143

Octostruma rugifera	-43,51552639	-19,22649834
Octostruma rugifera	-43,50768546	-19,21746248
Octostruma rugifera	-43,55193109	-19,25301164
Octostruma rugifera	-43,50796	-19,21721
Octostruma rugifera	-43,55207	-19,25299
Octostruma rugifera	-40,43279	-13,43588
Octostruma rugifera	-43,88194	-21,67889
Octostruma rugifera	-40,33972	-14,60186
Octostruma rugifera	-43,86667	-21,66667
Octostruma rugifera	-43,50562	-20,4278
Octostruma rugifera	-43,50857	-20,43377
Octostruma rugifera	-43,50857	-20,3377
Octostruma rugifera	-43,51477	-20,42596
Octostruma stenognatha	-43,514927	-20,426323
Octostruma stenognatha	-43,51261133	-19,21331143
Octostruma stenognatha	-43,51552639	-19,22649834
Octostruma stenognatha	-43,54058585	-19,23923389
Octostruma stenognatha	-43,61970456	-19,36750618
Octostruma stenognatha	-43,62102337	-19,36582779
Octostruma stenognatha	-43,5149	-20,4263
Octostruma stenognatha	-43,86667	-21,66667
Odontomachus bauri	-43,57581122	-19,22815804
Odontomachus bauri	-43,57328441	-19,23004179
Odontomachus bauri	-43,60725383	-19,26506046
Odontomachus bauri	-43,58776398	-19,26282191
Odontomachus bauri	-43,58853185	-19,26043608
Odontomachus bauri	-43,616667	-19,266667
Odontomachus bauri	-41,66667	-12,73333
Odontomachus brunneus	-43,58879512	-19,26152489
Odontomachus brunneus	-43,58374184	-19,2626567
Odontomachus brunneus	-44	-18
Odontomachus chelifer	-43,464747	-20,370645
Odontomachus chelifer	-43,5	-19,16666667
Odontomachus chelifer	-43,616667	-19,266667
Odontomachus chelifer	-43,55396	-19,24394
Odontomachus chelifer	-44	-18
Odontomachus chelifer	-43,5	-19,16667
Odontomachus chelifer	-43,68333	-20,51667
Odontomachus haematodus	-43,55412891	-19,24364579
Odontomachus haematodus	-43,5539166	-19,24407754
Odontomachus haematodus	-41,66667	-12,73333
Odontomachus haematodus	-44,03111	-20,05933
Odontomachus haematodus	-44,02138	-20,05955
Odontomachus haematodus	-40,33972	-14,60186
Odontomachus hastatus	-43,616667	-19,266667
Odontomachus meinerti	-43,60723883	-19,26576605
Odontomachus meinerti	-43,60600694	-19,2747227
Odontomachus meinerti	-43,58884516	-19,25988774

Odontomachus meinerti	-41,66667	-12,73333
Oxyepoecus browni	-43,616667	-19,266667
Oxyepoecus bruchi	-43,60609579	-19,26899141
Oxyepoecus bruchi	-40,33972	-14,60186
Oxyepoecus myops	-43,403	-19,041
Oxyepoecus rastratus	-43,9361	-20,2953
Oxyepoecus rastratus	-43,5	-20,13333
Oxyepoecus rastratus	-43,93674	-20,28669
Oxyepoecus rastratus	-43,9361	-20,2953
Oxyepoecus reticulatus	-44	-18
Pachycondyla bactronica	-41,25	-11,5
Pachycondyla carinulata	-44	-18
Pachycondyla crenata	-44	-18
Pachycondyla crenata	-43,45882	-20,23164
Pachycondyla crenata	-43,48828	-20,09808
Pachycondyla crenata	-43,51477	-20,42596
Pachycondyla crenata	-43,50821	-20,43387
Pachycondyla crenata	-43,50562	-20,4278
Pachycondyla crenata	-43,50857	-20,3377
Pachycondyla curvinodis	-40,85	-14,85
Pachycondyla harpax	-44,6475	-21,47139
Pachycondyla harpax	-43,54259047	-19,25948038
Pachycondyla harpax	-43,55222211	-19,24632213
Pachycondyla harpax	-43,57320461	-19,22974491
Pachycondyla harpax	-43,508628	-19,19963803
Pachycondyla harpax	-43,5154595	-19,22641461
Pachycondyla harpax	-43,54017296	-19,23919332
Pachycondyla harpax	-43,62089907	-19,36628007
Pachycondyla harpax	-43,6208735	-19,36834068
Pachycondyla harpax	-43,58805047	-19,26201842
Pachycondyla harpax	-43,58718286	-19,26190484
Pachycondyla harpax	-43,616667	-19,266667
Pachycondyla harpax	-43,51555	-19,22628
Pachycondyla harpax	-43,54059	-19,23923
Pachycondyla harpax	-43,54231	-19,25955
Pachycondyla harpax	-43,5523	-19,24623
Pachycondyla harpax	-43,50877	-19,1995
Pachycondyla harpax	-41,66667	-12,73333
Pachycondyla harpax	-44	-18
Pachycondyla harpax	-43,45882	-20,23164
Pachycondyla harpax	-40,33972	-14,60186
Pachycondyla harpax	-43,50857	-20,43377
Pachycondyla harpax	-43,50611	-20,4286
Pachycondyla harpax	-43,50857	-20,3377
Pachycondyla harpax	-43,51477	-20,42596
Pachycondyla impressa	-43,57703196	-19,22896966
Pachycondyla impressa	-43,60554166	-19,26825246
Pachycondyla impressa	-41,66667	-12,73333

Pachycondyla marginata	-43,643238	-19,770119
Pachycondyla marginata	-44	-18
Pachycondyla marginata	-43,48828	-20,09808
Pachycondyla marginata	-43,50562	-20,4278
Pachycondyla marginata	-43,50857	-20,3377
Pachycondyla marginata	-43,51477	-20,42596
Pachycondyla metanotalis	-44	-18
Pachycondyla moesta	-44	-18
Pachycondyla striata	-43,643238	-19,770119
Pachycondyla striata	-43,88194444	-21,67888889
Pachycondyla striata	-43,53454082	-19,2638678
Pachycondyla striata	-43,5359986	-19,26630769
Pachycondyla striata	-43,53830405	-19,26693684
Pachycondyla striata	-43,54255216	-19,25967802
Pachycondyla striata	-43,55222211	-19,24632213
Pachycondyla striata	-43,57665964	-19,22886573
Pachycondyla striata	-43,50898289	-19,19937015
Pachycondyla striata	-43,50768546	-19,21746248
Pachycondyla striata	-43,51099782	-19,21516165
Pachycondyla striata	-43,51328122	-19,21331612
Pachycondyla striata	-43,51561717	-19,22602904
Pachycondyla striata	-43,54017296	-19,23919332
Pachycondyla striata	-43,55236134	-19,25312371
Pachycondyla striata	-43,5556887	-19,24451843
Pachycondyla striata	-43,51239273	-19,23875252
Pachycondyla striata	-43,5171208	-19,25477696
Pachycondyla striata	-43,62044871	-19,36814563
Pachycondyla striata	-43,62138044	-19,36499219
Pachycondyla striata	-43,55552307	-19,29367712
Pachycondyla striata	-43,58839899	-19,26072735
Pachycondyla striata	-43,58338041	-19,2635513
Pachycondyla striata	-43,50796	-19,21721
Pachycondyla striata	-43,51081	-19,20915
Pachycondyla striata	-43,51284	-19,21324
Pachycondyla striata	-43,51555	-19,22628
Pachycondyla striata	-43,54059	-19,23923
Pachycondyla striata	-43,55207	-19,25299
Pachycondyla striata	-43,5523	-19,24623
Pachycondyla striata	-43,51269	-19,2387
Pachycondyla striata	-43,51695	-19,25501
Pachycondyla striata	-43,54231	-19,25955
Pachycondyla striata	-43,50877	-19,1995
Pachycondyla striata	-44	-18
Pachycondyla striata	-43,88333	-21,7
Pachycondyla striata	-43,88194	-21,67889
Pachycondyla striata	-43,45882	-20,23164
Pachycondyla striata	-43,49673	-20,2269
Pachycondyla striata	-43,50576	-20,21305

Pachycondyla striata	-43,50581	-20,19359
Pachycondyla striata	-43,50718	-20,19246
Pachycondyla striata	-43,5147	-20,18679
Pachycondyla striata	-43,52055	-20,18055
Pachycondyla striata	-43,51557	-20,16975
Pachycondyla striata	-40,33972	-14,60186
Pachycondyla striata	-43,68333	-20,51667
Pachycondyla striata	-43,86667	-21,66667
Pachycondyla striata	-43,48828	-20,09808
Pachycondyla striata	-43,45154	-20,35168
Pachycondyla striata	-43,50611	-20,4286
Pachycondyla striata	-43,50857	-20,43377
Pachycondyla striata	-43,50857	-20,3377
Pachycondyla striata	-43,51477	-20,42596
Pachycondyla striata	-43,616667	-19,266667
Pachycondyla unidentata	-41,66667	-12,73333
Pachycondyla unidentata	-44	-18
Pachycondyla villosa	-43,71	-18,9
Pachycondyla villosa	-44	-18
Pachycondyla villosa	-43,54167	-20,375
Pachycondyla villosa	-40,4308	-13,4411
Pachycondyla villosa	-43,50611	-20,4286
Paracryptocerus angustus	-44	-18
Paracryptocerus minutus	-44	-18
Paracryptocerus pallens	-44	-18
Paracryptocerus pinelii	-44	-18
Paracryptocerus pusillus	-44	-18
Paracryptocerus striativentris	-44	-18
Paratrechina longicornis	-43,009261	-16,861861
Paratrechina longicornis	-44	-18
Pheidole minensis	-44	-18
Pheidole ambigua	-43,50712312	-19,21733357
Pheidole ambigua	-43,51038812	-19,21493199
Pheidole ambigua	-43,51261133	-19,21331143
Pheidole ambigua	-43,54058585	-19,23923389
Pheidole arcifera	-44	-18
Pheidole capilata	-43,57590409	-19,23054487
Pheidole capilata	-43,58318394	-19,2638813
Pheidole cardinalis	-43,5	-20,13333
Pheidole claviscapa	-44	-18
Pheidole cordiceps	-44	-18
Pheidole diligens	-41,66667	-12,73333
Pheidole diligens	-43,50562	-20,4278
Pheidole diligens	-43,51477	-20,42596
Pheidole diligens	-43,50857	-20,3377
Pheidole dorsata	-43,57593703	-19,227891
Pheidole dorsata	-43,60717546	-19,26541954
Pheidole dorsata	-43,60725786	-19,266002

Pheidole dorsata	-43,60602505	-19,27408459
Pheidole dorsata	-43,55659034	-19,29551871
Pheidole dorsata	-43,58971546	-19,29438681
Pheidole emeryi	-44	-18
Pheidole fallax	-43,5	-20,4
Pheidole fallax	-44	-18
Pheidole fimbriata	-44	-18
Pheidole flavens	-43,53454082	-19,2638678
Pheidole flavens	-43,57539447	-19,23100102
Pheidole flavens	-43,57665964	-19,22886573
Pheidole flavens	-43,60592564	-19,26937329
Pheidole flavens	-43,55531571	-19,29406738
Pheidole flavens	-43,5574266	-19,2939668
Pheidole flavens	-43,59407036	-19,2956923
Pheidole flavens	-43,58389892	-19,26227113
Pheidole gertrudae	-43,53618284	-19,26587124
Pheidole gertrudae	-43,53398041	-19,26513791
Pheidole gertrudae	-43,57379302	-19,2279412
Pheidole gertrudae	-43,57761668	-19,22925783
Pheidole gertrudae	-43,57644095	-19,23024799
Pheidole gertrudae	-43,60558407	-19,27022221
Pheidole gertrudae	-43,60600694	-19,2747227
Pheidole gertrudae	-43,51261133	-19,21331143
Pheidole gertrudae	-43,60954595	-19,35895404
Pheidole gertrudae	-43,58971546	-19,29438681
Pheidole gertrudae	-43,58718286	-19,26190484
Pheidole gertrudae	-43,58869806	-19,26403485
Pheidole gertrudae	-43,643239	-19,770119
Pheidole gertrudae	-44	-18
Pheidole gertrudae	-43,56667	-18,18333
Pheidole fera	-44	-18
Pheidole fera	-44	-18
Pheidole senilis	-44	-18
Pheidole jelskii	-43,53618284	-19,26587124
Pheidole jelskii	-43,54217766	-19,25934853
Pheidole jelskii	-43,53830405	-19,26693684
Pheidole jelskii	-43,55257985	-19,24608199
Pheidole jelskii	-43,57761668	-19,22925783
Pheidole jelskii	-43,50898289	-19,19937015
Pheidole jelskii	-43,51283647	-19,21323683
Pheidole jelskii	-43,51328122	-19,21331612
Pheidole jelskii	-43,51543218	-19,22601705
Pheidole jelskii	-43,5402354	-19,23933799
Pheidole jelskii	-43,55204844	-19,2528342
Pheidole jelskii	-43,55571779	-19,24498463
Pheidole jelskii	-43,51291836	-19,2386407
Pheidole jelskii	-43,51239273	-19,23875252
Pheidole jelskii	-43,51693237	-19,25473991

Pheidole jelskii	-43,60516305	-19,26909995
Pheidole jelskii	-43,62007379	-19,36781379
Pheidole jelskii	-43,6212258	-19,36546158
Pheidole jelskii	-43,60935476	-19,35928596
Pheidole jelskii	-43,55681053	-19,29509978
Pheidole jelskii	-43,55417476	-19,29365398
Pheidole jelskii	-43,5929424	-19,29714278
Pheidole jelskii	-43,58821459	-19,26322608
Pheidole jelskii	-43,58617628	-19,26141224
Pheidole jelskii	-43,51081	-19,20915
Pheidole jelskii	-43,51555	-19,22628
Pheidole jelskii	-43,54059	-19,23923
Pheidole jelskii	-43,55207	-19,25299
Pheidole jelskii	-43,55573	-19,2447
Pheidole jelskii	-43,51269	-19,2387
Pheidole jelskii	-43,51695	-19,25501
Pheidole jelskii	-43,54231	-19,25955
Pheidole jelskii	-43,55089	-19,24794
Pheidole jelskii	-43,50877	-19,1995
Pheidole obscrurithorax	-43,88333	-21,7
Pheidole obscrurithorax	-44,02138	-20,05955
Pheidole obscurithorax	-43,88194444	-21,67888889
Pheidole obscurithorax	-43,643289	-19,770119
Pheidole obscurithorax	-44,03111	-20,05933
Pheidole obscurithorax	-44,01166	-20,05977
Pheidole obscurithorax	-43,88194	-21,67889
Pheidole oxyops	-43,5	-19,16666667
Pheidole oxyops	-43,54212686	-19,25955221
Pheidole oxyops	-43,53812065	-19,26736431
Pheidole oxyops	-43,53454082	-19,2638678
Pheidole oxyops	-43,55093289	-19,24823379
Pheidole oxyops	-43,57593703	-19,227891
Pheidole oxyops	-43,5771965	-19,23012117
Pheidole oxyops	-43,55219219	-19,24620646
Pheidole oxyops	-43,50886052	-19,19928314
Pheidole oxyops	-43,50898289	-19,19937015
Pheidole oxyops	-43,50784513	-19,21756575
Pheidole oxyops	-43,512842	-19,21340145
Pheidole oxyops	-43,51561717	-19,22602904
Pheidole oxyops	-43,54017296	-19,23919332
Pheidole oxyops	-43,55236134	-19,25312371
Pheidole oxyops	-43,51239273	-19,23875252
Pheidole oxyops	-43,5171208	-19,25477696
Pheidole oxyops	-43,60717546	-19,26541954
Pheidole oxyops	-43,60603527	-19,27437158
Pheidole oxyops	-43,60664757	-19,26921252
Pheidole oxyops	-43,60954595	-19,35895404
Pheidole oxyops	-43,60935476	-19,35928596

Pheidole oxyops	-43,55378919	-19,29447139
Pheidole oxyops	-43,58713743	-19,29451112
Pheidole oxyops	-43,5929424	-19,29714278
Pheidole oxyops	-43,58879512	-19,26152489
Pheidole oxyops	-43,58389892	-19,26227113
Pheidole oxyops	-43,50796	-19,21721
Pheidole oxyops	-43,51081	-19,20915
Pheidole oxyops	-43,51284	-19,21324
Pheidole oxyops	-43,51555	-19,22628
Pheidole oxyops	-43,54059	-19,23923
Pheidole oxyops	-43,55207	-19,25299
Pheidole oxyops	-43,55573	-19,2447
Pheidole oxyops	-43,51269	-19,2387
Pheidole oxyops	-43,51695	-19,25501
Pheidole oxyops	-43,54231	-19,25955
Pheidole oxyops	-43,5523	-19,24623
Pheidole oxyops	-43,50877	-19,1995
Pheidole oxyops	-44	-18
Pheidole oxyops	-43,5	-20,13333
Pheidole oxyops	-43,5	-19,16667
Pheidole oxyops	-44,26107	-17,82969
Pheidole oxyops	-43,45882	-20,23164
Pheidole oxyops	-43,68333	-20,51667
Pheidole oxyops	-43,594	-19,29
Pheidole praeses	-43,506465	-20,426694
Pheidole pubiventris	-44	-18
Pheidole radoskowskii	-43,88194	-21,67889
Pheidole radoszkowkii	-43,50562	-20,4278
Pheidole radoszkowkii	-43,51477	-20,42596
Pheidole radoszkowskii	-43,88194444	-21,67888889
Pheidole radoszkowskii	-43,53398041	-19,26513791
Pheidole radoszkowskii	-43,54259047	-19,25948038
Pheidole radoszkowskii	-43,53848334	-19,26651581
Pheidole radoszkowskii	-43,57297277	-19,22959956
Pheidole radoszkowskii	-43,55412891	-19,24364579
Pheidole radoszkowskii	-43,508628	-19,19963803
Pheidole radoszkowskii	-43,51054427	-19,21544027
Pheidole radoszkowskii	-43,51328122	-19,21331612
Pheidole radoszkowskii	-43,51566595	-19,22640212
Pheidole radoszkowskii	-43,5402354	-19,23933799
Pheidole radoszkowskii	-43,55204844	-19,2528342
Pheidole radoszkowskii	-43,5170695	-19,25526789
Pheidole radoszkowskii	-43,60592564	-19,26937329
Pheidole radoszkowskii	-43,60717546	-19,26541954
Pheidole radoszkowskii	-43,60603527	-19,27437158
Pheidole radoszkowskii	-43,62138044	-19,36499219
Pheidole radoszkowskii	-43,62044871	-19,36814563
Pheidole radoszkowskii	-43,60954595	-19,35895404

Pheidole radoszkowskii	-43,55681053	-19,29509978
Pheidole radoszkowskii	-43,55453208	-19,29292551
Pheidole radoszkowskii	-43,59183549	-19,29539139
Pheidole radoszkowskii	-43,58635783	-19,29374769
Pheidole radoszkowskii	-43,58318394	-19,2638813
Pheidole radoszkowskii	-43,58924758	-19,26275661
Pheidole radoszkowskii	-43,616667	-19,266667
Pheidole radoszkowskii	-43,51284	-19,21324
Pheidole radoszkowskii	-43,51555	-19,22628
Pheidole radoszkowskii	-43,54059	-19,23923
Pheidole radoszkowskii	-43,55207	-19,25299
Pheidole radoszkowskii	-43,51695	-19,25501
Pheidole radoszkowskii	-43,54231	-19,25955
Pheidole radoszkowskii	-43,55396	-19,24394
Pheidole radoszkowskii	-43,50877	-19,1995
Pheidole radoszkowskii	-43,88333	-21,7
Pheidole radoszkowskii	-43,88194	-21,67889
Pheidole radoszkowskii	-43,5148	-20,426
Pheidole radoszkowskii	-43,50585	-20,42815
Pheidole radoszkowskii	-44	-18
Pheidole radoszkowskii	-43,68333	-20,51667
Pheidole reclusi	-43,60707195	-19,268001
Pheidole reflexans	-43,50562	-20,4278
Pheidole reflexans	-43,50857	-20,43377
Pheidole reflexans	-43,51414	-20,4264
Pheidole reflexans	-43,50857	-20,3377
Pheidole schwarzmaieri	-44	-18
Pheidole geraesensis	-44	-18
Pheidole sensitiva	-43,60717546	-19,26541954
Pheidole sensitiva	-43,58887115	-19,26359874
Pheidole subarmata	-43,53618284	-19,26587124
Pheidole subarmata	-43,53812065	-19,26736431
Pheidole subarmata	-43,55085947	-19,24773901
Pheidole subarmata	-43,57630173	-19,22971783
Pheidole subarmata	-43,51099782	-19,21516165
Pheidole subarmata	-43,51328122	-19,21331612
Pheidole subarmata	-43,54017296	-19,23919332
Pheidole subarmata	-43,60592564	-19,26937329
Pheidole subarmata	-43,62102337	-19,36582779
Pheidole subarmata	-43,60954595	-19,35895404
Pheidole subarmata	-43,55453208	-19,29292551
Pheidole subarmata	-43,55417476	-19,29365398
Pheidole subarmata	-43,58944908	-19,26236669
Pheidole subarmata	-43,58887115	-19,26359874
Pheidole subarmata	-43,58318394	-19,2638813
Pheidole subarmata	-43,51081	-19,20915
Pheidole subarmata	-43,51284	-19,21324
Pheidole subarmata	-43,54059	-19,23923

Pheidole subarmata	-43,55089	-19,24794
Pheidole susannae	-43,53812065	-19,26736431
Pheidole susannae	-43,57761668	-19,22925783
Pheidole susannae	-43,57398514	-19,22811789
Pheidole susannae	-43,60602505	-19,27408459
Pheidole susannae	-43,60741091	-19,26746741
Pheidole susannae	-43,51552639	-19,22649834
Pheidole termitobla	-43,51552639	-19,22649834
Pheidole termitobla	-43,62007379	-19,36781379
Pheidole termitobla	-43,62138044	-19,36499219
Pheidole termitobla	-43,6208735	-19,36834068
Pheidole triconstricta	-43,53848334	-19,26651581
Pheidole triconstricta	-43,53379182	-19,26554787
Pheidole triconstricta	-43,57689064	-19,23097428
Pheidole triconstricta	-43,57610467	-19,23012905
Pheidole triconstricta	-43,60497655	-19,26951637
Pheidole triconstricta	-43,60935476	-19,35928596
Pheidole triconstricta	-43,55725486	-19,29432621
Pheidole triconstricta	-43,594	-19,29
Pheidole triconstricta	-44	-18
Pheidole vafra	-43,60707195	-19,268001
Pheidole vafra	-43,60600694	-19,2747227
Pheidole vafra	-43,51261133	-19,21331143
Pheidole vafra	-43,51552639	-19,22649834
Pheidole vafra	-43,62138044	-19,36499219
Pogonomyrmex abdominalis	-44	-18
Pogonomyrmex barbatus	-43,53830405	-19,26693684
Pogonomyrmex barbatus	-43,5752322	-19,22795009
Pogonomyrmex barbatus	-43,5768541	-19,22937987
Pogonomyrmex barbatus	-43,60558407	-19,27022221
Pogonomyrmex barbatus	-43,60609579	-19,26899141
Pogonomyrmex barbatus	-43,60916399	-19,35970422
Pogonomyrmex barbatus	-43,55497532	-19,29484162
Pogonomyrmex barbatus	-43,55378919	-19,29447139
Pogonomyrmex barbatus	-43,5935055	-19,29643543
Pogonomyrmex barbatus	-43,58821459	-19,26322608
Pogonomyrmex barbatus	-43,58389892	-19,26227113
Pogonomyrmex naegelii	-43,5386581	-19,26607518
Pogonomyrmex naegelii	-43,57703799	-19,23056072
Pogonomyrmex naegelii	-43,57590409	-19,23054487
Pogonomyrmex naegelii	-43,5402354	-19,23933799
Pogonomyrmex naegelii	-43,60477941	-19,26995122
Pogonomyrmex naegelii	-43,60935476	-19,35928596
Pogonomyrmex naegelii	-43,55659034	-19,29551871
Pogonomyrmex naegelii	-43,58792231	-19,29276416
Pogonomyrmex naegelii	-43,59419558	-19,29766774
Pogonomyrmex naegelii	-43,58869806	-19,26403485
Pogonomyrmex naegelii	-43,54059	-19,23923

Pogonomyrmex naegelii	-40,43292	-13,43694
Pogonomyrmex naegelii	-43,5	-20,13333
Pogonomyrmex naegelii	-44,03111	-20,05933
Pogonomyrmex naegelii	-44,01166	-20,05977
Pogonomyrmex naegelii	-44,02138	-20,05955
Pogonomyrmex naegelii	-43,68333	-20,51667
Ponera distinguenda	-44	-18
Ponera foreli	-44	-18
Ponera iheringi	-44	-18
Ponera levillei	-44	-18
Ponera opaciceps	-44	-18
Prionopelta punctulata	-43,55709803	-19,29470834
Prionopelta punctulata	-43,58713743	-19,29451112
Prionopelta punctulata	-43,58846588	-19,26102131
Probolomyrmex brujitae	-40,33972	-14,60186
Procryptocerus adlerzi	-43,5	-20,13333
Procryptocerus adlerzi	-44	-18
Procryptocerus goeldii	-43,5	-20,13333333
Procryptocerus goeldii	-43,5	-20,13333
Procryptocerus goeldii	-44	-18
Procryptocerus hylaeus	-43,616667	-19,266667
Procryptocerus lepidus	-43,5	-20,13333
Procryptocerus lepidus	-44	-18
Procryptocerus montanus	-43,5526713	-19,2462011
Procryptocerus montanus	-43,55222211	-19,24632213
Procryptocerus montanus	-43,50886052	-19,19928314
Procryptocerus montanus	-43,50898289	-19,19937015
Procryptocerus montanus	-43,51328122	-19,21331612
Procryptocerus montanus	-43,50857	-20,3377
Procryptocerus montanus	-43,50562	-20,4278
Procryptocerus montanus	-43,51477	-20,42596
Procryptocerus montanus	-43,5	-20,13333
Procryptocerus montanus	-43,51284	-19,21324
Procryptocerus montanus	-43,5523	-19,24623
Procryptocerus montanus	-43,50877	-19,1995
Procryptocerus montanus	-44	-18
Procryptocerus montanus	-43,51429	-20,42627
Procryptocerus montanus	-43,50871	-20,43397
Procryptocerus montanus	-43,50536	-20,42768
Procryptocerus montanus	-43,51487	-20,42558
Procryptocerus sampaioi	-44	-18
Procryptocerus schmalzi	-43,5	-20,13333
Procryptocerus schmalzi	-44	-18
Procryptocerus schmitti	-43,60717546	-19,26541954
Procryptocerus schmitti	-43,60612437	-19,27350305
Procryptocerus schmitti	-43,58672152	-19,26158968
Procryptocerus sulcatus	-44	-18
Pseudomyrmex adustus	-44	-18

Pseudomyrmex cubaensis	-43,61694	-19,38417
Pseudomyrmex cubensis	-43,60725383	-19,26506046
Pseudomyrmex curacaensis	-44	-18
Pseudomyrmex elongatulus	-44	-18
Pseudomyrmex elongatus	-44	-18
Pseudomyrmex elongatus	-43,61694	-19,38417
Pseudomyrmex gracilis	-43,586907	-19,352594
Pseudomyrmex gracilis	-43,5171208	-19,25477696
Pseudomyrmex gracilis	-43,60935476	-19,35928596
Pseudomyrmex gracilis	-43,60916399	-19,35970422
Pseudomyrmex gracilis	-43,58944908	-19,26236669
Pseudomyrmex gracilis	-43,616667	-19,266667
Pseudomyrmex gracilis	-43,51695	-19,25501
Pseudomyrmex gracilis	-44	-18
Pseudomyrmex gracilis	-43,89693	-21,71422
Pseudomyrmex gracilis	-43,50896	-20,43476
Pseudomyrmex gracilis	-43,67244	-20,34953
Pseudomyrmex gracilis	-43,594	-19,29
Pseudomyrmex gracilis	-43,50667	-20,4286
Pseudomyrmex gracillis	-44	-18
Pseudomyrmex gracillis	-43,50562	-20,4278
Pseudomyrmex gracillis	-43,50857	-20,3377
Pseudomyrmex gracillis	-43,51477	-20,42596
Pseudomyrmex kuenckeli	-44	-18
Pseudomyrmex laevivertex	-43,50562	-20,4278
Pseudomyrmex laevivertex	-43,50878	-20,43416
Pseudomyrmex laevivertex	-43,50635	-20,42821
Pseudomyrmex laevivertex	-43,50584	-20,4278
Pseudomyrmex laevivertex	-43,51443	-20,42619
Pseudomyrmex laevivertex	-43,50667	-20,4286
Pseudomyrmex longus	-43,5	-20,13333
Pseudomyrmex mutica	-44	-18
Pseudomyrmex oculatus	-43,616667	-19,266667
Pseudomyrmex palidus	-43,50667	-20,4286
Pseudomyrmex palidus	-43,50611	-20,4286
Pseudomyrmex pallidus	-43,56667	-18,18333
Pseudomyrmex pallidus	-43,68333	-20,51667
Pseudomyrmex pallidus	-43,49715	-20,36933
Pseudomyrmex pallidus	-43,51444	-20,44055
Pseudomyrmex pallidus	-43,594	-19,29
Pseudomyrmex phillophilus	-43,51446	-20,42615
Pseudomyrmex phillophilus	-43,51444	-20,42601
Pseudomyrmex phillophilus	-43,50653	-20,42841
Pseudomyrmex phillophilus	-43,50529	-20,42786
Pseudomyrmex phyllophilus	-44	-18
Pseudomyrmex phyllophilus	-43,5	-20,38333
Pseudomyrmex rufiventris	-43,94208	-20,09783
Pseudomyrmex schuppi	-43,5	-20,13333

Pseudomyrmex schuppi	-44	-18
Pseudomyrmex schuppi	-43,6	-18,25
Pseudomyrmex schuppi	-43,5	-20,38333
Pseudomyrmex schuppi	-43,61694	-19,38417
Pseudomyrmex schuppi	-43,5059	-20,428
Pseudomyrmex schuppi	-43,5148	-20,42593
Pseudomyrmex schuppi	-43,50865	-20,4348
Pseudomyrmex shuppi	-43,67244	-20,34953
Pseudomyrmex simplex	-43,56667	-18,18333
Pseudomyrmex tenuis	-41,66667	-12,73333
Pseudomyrmex tenuissimus	-44	-18
Pseudomyrmex tenuissimus	-43,61694	-19,38417
Pseudomyrmex terminalis	-43,57761668	-19,22925783
Pseudomyrmex terminalis	-43,51693237	-19,25473991
Pseudomyrmex terminalis	-43,60664757	-19,26921252
Pseudomyrmex terminalis	-43,60916399	-19,35970422
Pseudomyrmex terminalis	-43,55709803	-19,29470834
Pseudomyrmex terminalis	-43,59380012	-19,29607602
Pseudomyrmex terminalis	-43,58869806	-19,26403485
Pseudomyrmex terminalis	-43,58862606	-19,26193929
Pseudomyrmex terminalis	-43,58374184	-19,2626567
Pseudomyrmex termitarius	-43,643238	-19,770119
Pseudomyrmex termitarius	-43,5386581	-19,26607518
Pseudomyrmex termitarius	-43,57665964	-19,22886573
Pseudomyrmex termitarius	-43,57590409	-19,23054487
Pseudomyrmex termitarius	-43,60592564	-19,26937329
Pseudomyrmex termitarius	-43,60916399	-19,35970422
Pseudomyrmex termitarius	-43,55417476	-19,29365398
Pseudomyrmex termitarius	-43,58792231	-19,29276416
Pseudomyrmex termitarius	-43,59320551	-19,29679611
Pseudomyrmex termitarius	-43,58887115	-19,26359874
Pseudomyrmex termitarius	-43,58862606	-19,26193929
Pseudomyrmex termitarius	-43,58374184	-19,2626567
Pseudomyrmex termitarius	-44	-18
Pseudomyrmex termitarius	-43,89693	-21,71422
Pseudomyrmex termitarius	-43,594	-19,29
Pseudomyrmex termitarius	-43,5	-20,38333
Pseudomyrmex termitarius	-43,57665	-19,22648
Pseudomyrmex termitarius	-43,54992	-19,2704
Pseudomyrmex termitarius	-43,56667	-18,18333
Pseudomyrmex termitarius	-43,50562	-20,4278
Pseudomyrmex termitarius	-43,50857	-20,43377
Pseudomyrmex unicolor	-44	-18
Pseudomyrmex unicolor	-43,61694	-19,38417
Pseudomyrmex urbanus	-44	-18
Rasopone arhuaca	-43,616667	-19,266667
Rogeria bruchi	-43,57540621	-19,22820054
Rogeria ciliosa	-40,33972	-14,60186

Rogeria minensis	-44	-18
Rogeria minensis	-44	-18
Sericomyrmex luederwaldti	-44	-18
Sericomyrmex mayri	-41,5005	-12,9053
Sericomyrmex mayri	-41,5005	-12,9053
Sericomyrmex parvulus	-41,6833	-12,55
Sericomyrmex parvulus	-41,6833	-12,55
Sericomyrmex scrobifer	-40,84	-14,84
Sericomyrmex scrobifer	-40,84	-14,84
Simopelta curvata	-44	-18
Simopelta curvata	-43,5	-20,13333
Simopelta curvata	-43,66437	-20,00434
Smithistruma epinotalis	-44	-18
Solenopsis basalis	-44	-18
Solenopsis bondari	-43,5	-19,16667
Solenopsis brevicornis	-44	-18
Solenopsis clytemnestra	-44	-18
Solenopsis clytemnestra	-44	-18
Solenopsis geminata	-40,43279	-13,43588
Solenopsis globularia	-44	-18
Solenopsis globularia	-44,26114	-17,82736
Solenopsis globularis	-43,53379182	-19,26554787
Solenopsis globularis	-43,5774275	-19,22970358
Solenopsis globularis	-43,60609579	-19,26899141
Solenopsis globularis	-43,55453208	-19,29292551
Solenopsis globularis	-43,5574266	-19,2939668
Solenopsis globularis	-43,59380012	-19,29607602
Solenopsis globularis	-43,5935055	-19,29643543
Solenopsis globularis	-43,58389892	-19,26227113
Solenopsis globularis	-43,58354487	-19,2630799
Solenopsis invicta	-43,87111111	-20,02111111
Solenopsis invicta	-43,89027778	-20,06416667
Solenopsis invicta	-43,50562	-20,4278
Solenopsis invicta	-43,50857	-20,43377
Solenopsis invicta	-43,50857	-20,3377
Solenopsis invicta	-43,51477	-20,42596
Solenopsis pollux	-40,33972	-14,60186
Solenopsis saevissima	-42,1	-12,4
Solenopsis saevissima	-43,5	-19,16666667
Solenopsis saevissima	-43,57650792	-19,22930259
Solenopsis saevissima	-43,60610191	-19,27049839
Solenopsis saevissima	-43,60916399	-19,35970422
Solenopsis saevissima	-43,58924758	-19,26275661
Solenopsis saevissima	-43,53848334	-19,26651581
Solenopsis saevissima	-43,53454082	-19,2638678
Solenopsis saevissima	-43,57540621	-19,22820054
Solenopsis saevissima	-43,57610467	-19,23012905
Solenopsis saevissima	-43,60612437	-19,27350305

Solenopsis saevissima	-43,60554166	-19,26825246
Solenopsis saevissima	-43,61970456	-19,36750618
Solenopsis saevissima	-43,60916399	-19,35970422
Solenopsis saevissima	-43,55569591	-19,29324931
Solenopsis saevissima	-43,55659034	-19,29551871
Solenopsis saevissima	-43,59380012	-19,29607602
Solenopsis saevissima	-43,58749366	-19,29428162
Solenopsis saevissima	-43,58776398	-19,26282191
Solenopsis saevissima	-43,58646127	-19,26144719
Solenopsis saevissima	-43,58617628	-19,26141224
Solenopsis saevissima	-43,58338041	-19,2635513
Solenopsis saevissima	-43,616667	-19,266667
Solenopsis saevissima	-44	-18
Solenopsis saevissima	-43,5	-19,16667
Solenopsis saevissima	-43,45882	-20,23164
Solenopsis saevissima	-43,44961	-20,08184
Solenopsis substituta	-42,1	-12,4
Solenopsis substituta	-43,53830405	-19,26693684
Solenopsis substituta	-43,5768541	-19,22937987
Solenopsis substituta	-43,60609579	-19,26899141
Solenopsis substituta	-43,60935476	-19,35928596
Solenopsis substituta	-43,55378919	-19,29447139
Solenopsis substituta	-43,59183549	-19,29539139
Solenopsis substituta	-43,59320551	-19,29679611
Solenopsis substituta	-43,58869806	-19,26403485
Solenopsis substituta	-43,58879512	-19,26152489
Solenopsis substituta	-43,58389892	-19,26227113
Solenopsis substituta	-43,58374184	-19,2626567
Solenopsis substituta	-43,53398041	-19,26513791
Solenopsis substituta	-43,5774275	-19,22970358
Solenopsis substituta	-43,60477941	-19,26995122
Solenopsis substituta	-43,60916399	-19,35970422
Solenopsis substituta	-43,55417476	-19,29365398
Solenopsis substituta	-43,58864483	-19,2933773
Solenopsis substituta	-43,58944908	-19,26236669
Solenopsis substituta	-43,58389892	-19,26227113
Solenopsis sulfurea	-41,53552	-13,27808
Solenopsis tridens	-42,1	-12,4
Solenopsis virulens	-43,5	-19,16666667
Solenopsisis geminata	-42,1	-12,4
Sphinctomyrmex stali	-43,54212686	-19,25955221
Sphinctomyrmex stali	-43,54231	-19,25955
Strumigenys appretiata	-41,66667	-12,73333
Strumigenys crassicornis	-43,551796	-19,252981
Strumigenys crassicornis	-43,506466	-20,426695
Strumigenys crassicornis	-43,51261133	-19,21331143
Strumigenys crassicornis	-43,51552639	-19,22649834
Strumigenys crassicornis	-43,61970456	-19,36750618

Strumigenys crassicornis	-44	-18
Strumigenys crassicornis	-43,5149	-20,4263
Strumigenys denticulata	-43,61726	-19,37294
Strumigenys denticulata	-43,5752322	-19,22795009
Strumigenys denticulata	-43,57360393	-19,23045502
Strumigenys denticulata	-43,51566595	-19,22640212
Strumigenys denticulata	-43,60725786	-19,266002
Strumigenys denticulata	-43,605945	-19,273767
Strumigenys denticulata	-43,51038812	-19,21493199
Strumigenys denticulata	-43,58646127	-19,26144719
Strumigenys denticulata	-41,66667	-12,73333
Strumigenys denticulata	-44	-18
Strumigenys denticulata	-43,51555	-19,22628
Strumigenys eggersi	-43,57360393	-19,23045502
Strumigenys eggersi	-43,5574266	-19,2939668
Strumigenys elongata	-43,6181	-19,367664
Strumigenys elongata	-43,5752322	-19,22795009
Strumigenys elongata	-43,60739406	-19,26632957
Strumigenys elongata	-43,60725383	-19,26506046
Strumigenys elongata	-43,60612437	-19,27350305
Strumigenys elongata	-43,51038812	-19,21493199
Strumigenys elongata	-43,5574266	-19,2939668
Strumigenys elongata	-40,33972	-14,60186
Strumigenys louisianae	-43,88194444	-21,67888889
Strumigenys louisianae	-43,53618284	-19,26587124
Strumigenys louisianae	-43,53398041	-19,26513791
Strumigenys louisianae	-43,53379182	-19,26554787
Strumigenys louisianae	-43,88194	-21,67889
Strumigenys oglobini	-43,59201838	-19,29497782
Strumigenys perparva	-41,66667	-12,73333
Strumigenys reticeps	-43,588673	-19,26015847
Strumigenys saliens	-43,905556	-21,680616
Strumigenys saliens	-44	-18
Strumigenys schulzi	-43,60916399	-19,35970422
Strumigenys schulzi	-43,5929424	-19,29714278
Tapinoma atriceps	-43,55	-19,25
Tapinoma atriceps	-43,52	-19,25
Tapinoma atriceps	-43,5768541	-19,22937987
Tapinoma atriceps	-43,51067009	-19,21557186
Tapinoma atriceps	-43,51566595	-19,22640212
Tapinoma atriceps	-43,55586841	-19,24453829
Tapinoma atriceps	-43,60592564	-19,26937329
Tapinoma atriceps	-43,51038812	-19,21493199
Tapinoma atriceps	-43,62044871	-19,36814563
Tapinoma atriceps	-43,58887115	-19,26359874
Tapinoma atriceps	-43,58338041	-19,2635513
Tapinoma atriceps	-43,51081	-19,20915
Tapinoma atriceps	-43,51555	-19,22628

Tapinoma atriceps	-43,55573	-19,2447
Tapinoma atriceps	-43,86667	-21,66667
Tapinoma atriceps	-43,48664	-20,09866
Tapinoma atriceps	-43,50611	-20,4286
Tapinoma melanocephalum	-43,931855	-19,978165
Tapinoma melanocephalum	-43,57689064	-19,23097428
Tapinoma melanocephalum	-43,60634339	-19,26858036
Tapinoma melanocephalum	-43,60634255	-19,27007644
Tapinoma melanocephalum	-43,55453208	-19,29292551
Tapinoma melanocephalum	-43,58318394	-19,2638813
Tapinoma melanocephalum	-43,54167	-20,375
Tapinoma melanocephalum	-43,50562	-20,4278
Tapinoma melanocephalum	-43,50857	-20,3377
Termitopone commutata	-44	-18
Termitopone marginata	-44	-18
Tetramorium simillimum	-44	-18
Thaumatomyrmex atrox	-40,43279	-13,43588
Thaumatomyrmex atrox	-40,4308	-13,4411
Thaumatomyrmex contumax	-40,43279	-13,43588
Thaumatomyrmex contumax	-40,4308	-13,4411
Thaumatomyrmex fraxini	-40,85	-14,85
Trachymyrmex dichrous	-44	-18
Trachymyrmex holmgreni	-43,67242	-20,34903
Trachymyrmex holmgreni	-43,67222	-20,34889
Trachypheidole aper	-44	-18
Tranopelta amblyops	-44	-18
Tranopelta gilva	-41,3698	-12,9963
Tranopelta gilva	-43,5771965	-19,23012117
Tranopelta gilva	-43,60516305	-19,26909995
Tranopelta gilva	-43,60916399	-19,35970422
Tranopelta gilva	-41,66667	-12,73333
Tranopelta gilva	-41,3698	-12,9963
Typhlomyrmex major	-43,86667	-21,66667
Typhlomyrmex rogenhoferi	-43,5	-20,13333
Typhlomyrmex rogenhoferi	-44	-18
Wasmannia affines	-43,50857	-20,3377
Wasmannia affines	-43,50562	-20,4278
Wasmannia affines	-43,51477	-20,42596
Wasmannia affinis	-43,514927	-20,426323
Wasmannia affinis	-43,88194444	-21,67888889
Wasmannia affinis	-43,51038812	-19,21493199
Wasmannia affinis	-43,51261133	-19,21331143
Wasmannia affinis	-43,51552639	-19,22649834
Wasmannia affinis	-43,61970456	-19,36750618
Wasmannia affinis	-43,88194	-21,67889
Wasmannia affinis	-43,5149	-20,4263
Wasmannia affinis	-43,50562	-20,4278
Wasmannia affinis	-43,50857	-20,43377

Wasmannia affinis	-43,50667	-20,4286
Wasmannia affinis	-43,50611	-20,4286
Wasmannia auropunctata	-43,88194444	-21,67888889
Wasmannia auropunctata	-43,53673093	-19,26461413
Wasmannia auropunctata	-43,57540621	-19,22820054
Wasmannia auropunctata	-43,57379302	-19,2279412
Wasmannia auropunctata	-43,60725786	-19,266002
Wasmannia auropunctata	-43,60477941	-19,26995122
Wasmannia auropunctata	-43,55516265	-19,29446225
Wasmannia auropunctata	-43,55497532	-19,29484162
Wasmannia auropunctata	-43,59419558	-19,29766774
Wasmannia auropunctata	-43,58674466	-19,29347931
Wasmannia auropunctata	-43,616667	-19,266667
Wasmannia auropunctata	-43,51017	-20,43486
Wasmannia auropunctata	-41,66667	-12,73333
Wasmannia auropunctata	-44,03111	-20,05933
Wasmannia auropunctata	-44,01166	-20,05977
Wasmannia auropunctata	-44	-18
Wasmannia auropunctata	-43,88194	-21,67889
Wasmannia auropunctata	-44,02138	-20,05955
Wasmannia auropunctata	-43,50808	-20,21398
Wasmannia auropunctata	-43,51696	-20,19747
Wasmannia auropunctata	-43,5147	-20,18679
Wasmannia auropunctata	-43,52055	-20,18055
Wasmannia auropunctata	-43,51557	-20,16975
Wasmannia auropunctata	-43,50599	-20,19277
Wasmannia auropunctata	-40,33972	-14,60186
Wasmannia auropunctata	-43,594	-19,29
Wasmannia auropunctata	-43,50857	-20,3377
Wasmannia auropunctata	-43,50562	-20,4278
Wasmannia auropunctata	-43,51477	-20,42596
Wasmannia lutzi	-43,53418258	-19,26471345
Wasmannia lutzi	-43,54255216	-19,25967802
Wasmannia lutzi	-43,55096064	-19,24777488
Wasmannia lutzi	-43,57644095	-19,23024799
Wasmannia lutzi	-43,50870855	-19,19974214
Wasmannia lutzi	-43,51099782	-19,21516165
Wasmannia lutzi	-43,51552639	-19,22649834
Wasmannia lutzi	-43,54063815	-19,23901387
Wasmannia lutzi	-43,55193109	-19,25301164
Wasmannia lutzi	-43,55586841	-19,24453829
Wasmannia lutzi	-43,51249022	-19,23886878
Wasmannia lutzi	-43,51276833	-19,23850274
Wasmannia lutzi	-43,5170695	-19,25526789
Wasmannia lutzi	-43,60558407	-19,27022221
Wasmannia lutzi	-43,61970456	-19,36750618
Wasmannia lutzi	-43,6208735	-19,36834068
Wasmannia lutzi	-43,55681053	-19,29509978

Wasmannia lutzi	-43,5929424	-19,29714278
Wasmannia lutzi	-43,58805047	-19,26201842
Wasmannia lutzi	-43,51081	-19,20915
Wasmannia lutzi	-43,51555	-19,22628
Wasmannia lutzi	-43,54059	-19,23923
Wasmannia lutzi	-43,55207	-19,25299
Wasmannia lutzi	-43,55089	-19,24794
Wasmannia lutzi	-43,51269	-19,2387
Wasmannia lutzi	-43,51695	-19,25501
Wasmannia lutzi	-43,54231	-19,25955
Wasmannia lutzi	-43,50877	-19,1995
Wasmannia lutzi	-43,68333	-20,51667
Wasmannia rochai	-44	-18
Wasmannia rochai	-43,50667	-20,4286
Wasmannia sigmoidea	-43,49673	-20,2269

Code S1. Cleaning and modelling code

```
pkg=c("CoordinateCleaner","devtools","VSURF","tmap","dismo","raster","rgdal","ran
d omForest",
"maptools","sp","rgeos","ncdf4","maps","foreach","doParallel","rnaturalearth","SSDM",
"magrittr","tidyverse","readxl","dplyr","rgbif","tiff")

lapply(pkg, require, character.only=T)


####import data from data file.csv####

#File -> Import dataset -> From excel




####Variables in Campos rupestres - Importei todas as variáveis ambientais,
coloquei uma opção para importar a partir de um arquivo único ou a partir de
vários####

#alternative with one file

setwd("/home/esturdivant/DAVI/TCC DAVI 4.0")

predictors <- stack("C:/Users/daviv/OneDrive/Área de Trabalho/Modeling/variables
2081-2100/pessimistic/SN/5 min/Earth_veg/ssp_585_veg.tif")
```

```
writeRaster(predictors, paste0(names(predictors),".tif"), bylayer=TRUE,
format="GTiff")

plot(predictors)


#alternative with a list of files

espinhaco_files <- list.files(path = "/home/esturdivant/DAVI/TCC DAVI
4.0/wc2.1_2.5m_bio", pattern='tif', full.names=TRUE)

predictors <- stack(espinhaco_files)

plot(predictors)


#establishment of an area to crop environmental data - Cortei minhas variáveis para
o shapefile utilizado

AREA=shapefile("/home/esturdivant/DAVI/cadeia do espinhaco.shp")

AREA=shapefile("C:/Users/daviv/OneDrive/Área de
Trabalho/Modeling/shapefiles/shapefile_CR/cadeia do espinhaco.shp")

projection(AREA)="+proj=longlat +datum=WGS84"

plot(AREA

) library(sf)

shapefile_path <- "C:/Users/daviv/OneDrive/Área de
Trabalho/Modeling/shapefiles/shapefile_CR/cr.shp"

cr_shape <- st_read(shapefile_path)

str(cr_shape)

nrow(cr_shape)


####opção para pegar shapefile das regioes do brasil####

#how to crop some regions of a brasil.shp#

regions <- c("Nordeste", "Sudeste")

subset_shapefile <- AREA[AREA$NM_REGIAO %in% regions,]

projection(subset_shapefile)="+proj=longlat +datum=WGS84"
```

writeOGR(obj = subset_shapefile, dsn = "C:/Users/daviv/OneDrive/Área de Trabalho/Modeling/ShapefilesSN/shapefileSN.shp", layer = "subset_shapefile", driver = "ESRI Shapefile")

projection(AREA)="+proj=longlat +datum=WGS84"

plot(AREA)

#or

Load the natural earth data for Brazil brazil

<- ne_states(country = "brazil")

Define the regions (states) you want to extract regions

<- c("Minas Gerais", "Bahia")

Subset the data to get polygons for Minas Gerais and Bahia

subset_shapefile <- brazil[brazil$name %in% regions,]

Set the projection for the subset_shapefile

st_crs(subset_shapefile) <- "+proj=longlat +datum=WGS84"

projection(subset_shapefile) <- "+proj=longlat +datum=WGS84"

Write the combined shapefile containing both states

writeOGR(obj = subset_shapefile, dsn = "C:/Users/daviv/OneDrive/Área de Trabalho/Modeling/shapefiles/ShapefileMGBA//MinasGerais_Bahia.shp", layer = "MinasGerais_Bahia", driver = "ESRI Shapefile")

Plot the subset_shapefile (optional)

plot(subset_shapefile)

####crop variables on the shapefile - cortei cada uma das variáveis para meu shapefile#####

```
crop <- crop(predictors, AREA, snap="out")

predictors_calibration=stack(crop)

plot(predictors_calibration)

mask <- mask(predictors_calibration, AREA)

plot(mask)

predictors_final=stack(mask)

plot(predictors_final)


bio1=raster(predictors_final, 1)

writeRaster(bio1, filename = "C:/Users/daviv/OneDrive/Área de Trabalho/Modeling
new/1970CR//Annual temperature.tif", format = "GTiff", overwrite = TRUE)


bio2=raster(predictors_final, 2)

writeRaster(bio2, filename = "C:/Users/daviv/OneDrive/Área de Trabalho/Modeling
new/1970/Mean Temperature of Warmest Quarter.tif", format = "GTiff", overwrite =
TRUE )


bio3=raster(predictors_final,3)

writeRaster(bio3, filename = "C:/Users/daviv/OneDrive/Área de Trabalho/Modeling
new/1970/Mean Temperature of Coldest Quarter.tif", format = "GTiff", overwrite =
TRUE)


bio4=raster(predictors_final,4)

writeRaster(bio4, filename = "C:/Users/daviv/OneDrive/Área de Trabalho/Modeling
new/1970/Annual Precipitation.tif", format = "GTiff", overwrite = TRUE)


bio5=raster(predictors_final,5)

writeRaster(bio5, filename = "C:/Users/daviv/OneDrive/Área de Trabalho/Modeling
new/1970/Precipitation of Wettest Month.tif", format = "GTiff", overwrite = TRUE)
```

```
bio6=raster(predictors_final,6)

writeRaster(bio6, filename = "C:/Users/daviv/OneDrive/Área de Trabalho/Modeling new/1970/Precipitation of Driest Month.tif", format = "GTiff", overwrite = TRUE)


bio7=raster(predictors_final,7)

writeRaster(bio7, filename = "C:/Users/daviv/OneDrive/Área de Trabalho/Modeling new/1970/Precipitation Seasonality (Coefficient of Variation).tif", format = "GTiff", overwrite = TRUE)


bio8=raster(predictors_final,8)

writeRaster(bio8, filename = "C:/Users/daviv/OneDrive/Área de Trabalho/Modeling new/1970/Precipitation of Wettest Quarter.tif", format = "GTiff", overwrite = TRUE)


bio9=raster(predictors_final,9)

writeRaster(bio9, filename = "C:/Users/daviv/OneDrive/Área de Trabalho/Modeling new/1970/Precipitation of Driest Quarter.tif", format = "GTiff", overwrite = TRUE)


bio10=raster(predictors_final,10)

writeRaster(bio10, filename = "C:/Users/daviv/OneDrive/Área de Trabalho/Modeling new/1970/Precipitation of Warmest Quarter.tif", format = "GTiff", overwrite = TRUE)


bio11=raster(predictors_final,11)

writeRaster(bio11, filename = "C:/Users/daviv/OneDrive/Área de Trabalho/Modeling new/1970/Precipitation of Coldest Quarter.tif", format = "GTiff", overwrite = TRUE)


bio12=raster(predictors_final,12)

writeRaster(bio12, filename = "C:/Users/daviv/OneDrive/Área de Trabalho/Modeling new/1970/Mean Diurnal Range (Mean of monthly (max temp - min temp)).tif", format = "GTiff", overwrite = TRUE)
```

```
bio13=raster(predictors_final,13)

writeRaster(bio13, filename = "C:/Users/daviv/OneDrive/Área de Trabalho/Modeling new/1970/Isothermality.tif", format = "GTiff", overwrite = TRUE)

bio14=raster(predictors_final,14)

writeRaster(bio14, filename = "C:/Users/daviv/OneDrive/Área de Trabalho/Modeling new/1970/Temperature Seasonality.tif", format = "GTiff",overwrite = TRUE)

bio15=raster(predictors_final,15)

writeRaster(bio15, filename = "C:/Users/daviv/OneDrive/Área de Trabalho/Modeling new/1970/Max Temperature of Warmest Month.tif", format = "GTiff", overwrite = TRUE)

bio16=raster(predictors_final,16)

writeRaster(bio16, filename = "C:/Users/daviv/OneDrive/Área de Trabalho/Modeling new/1970/Min Temperature of Coldest Month.tif", format = "GTiff",overwrite = TRUE)

bio17=raster(predictors_final,17)

writeRaster(bio17, filename = "C:/Users/daviv/OneDrive/Área de Trabalho/Modeling new/1970/Temperature Annual Range.tif", format = "GTiff",overwrite = TRUE)

bio18=raster(predictors_final,18)

writeRaster(bio18, filename = "C:/Users/daviv/OneDrive/Área de Trabalho/Modeling new/1970/Mean Temperature of Wettest Quarter.tif", format = "GTiff",overwrite = TRUE)

bio19=raster(predictors_final,19)
```

```
writeRaster(bio19, filename = "C:/Users/daviv/OneDrive/Área de
Trabalho/Modeling new/1970/Mean Temperature of Driest Quarter.tif", format =
"GTiff",overwrite = TRUE)


bio20=raster(predictors_final,20)

writeRaster(bio20, filename = "C:/Users/daviv/OneDrive/Área de Trabalho/Modeling
new/1970/Elevation.tif", format = "GTiff",overwrite = TRUE)



save(predictors_final, file = "predictors_final.rda",overwrite=T)

load(file = "predictors_final.rda")


####Get occurrence data ants from my region####

key <- name_backbone(name = "Formicidae")$usageKey

genus1=occ_search(taxonKey = key,country = "BR",limit=67000,year =

1970:2023,) genus1 = GBIF_CURRENT_0084530_230530130749713_csv

names(genus1)

genus1=genus1$data

names(genus1)

latlon=cbind(genus1$genus,genus1$decimalLongitude,genus1$decimalLatitude)

colnames(latlon) <- c("genus","lon","lat")

latlon = as.data.frame(latlon)

Species_formicidae=na.omit(latlon)

write.csv(Species_formicidae,"C:/Users/daviv/OneDrive/Área de
Trabalho/Modeling/raw_data/genus_gbif.csv", row.names = FALSE)

write.csv(Genus_formicidae,"C:/Users/daviv/OneDrive/Documentos/program/data
/separed data/Genus_formicidae.csv", row.names = FALSE)


#merge the three (gbif,mata e lab)# df1

<- read.csv("genus_gbif.csv")
```

```
df1 <- as.data.frame(df1)

df2 <- read.csv("genus_lab.csv")

df2 <- as.data.frame(df2)

df3 <- read.csv("genus_mata.csv")

df3 <- as.data.frame(df3)

df3 <- na.omit(df3)

merged_df <- rbind(df2, df3)

write.csv(merged_df,"C:/Users/daviv/OneDrive/Área de
Trabalho/Modeling/raw_data/genus_LM.csv", row.names =
FALSE)

setwd("C:/Users/daviv/OneDrive/Área de Trabalho/Modeling/raw_data")

genus_LM <- read.csv("genus_LGM.csv")



#Recortei as ocorrencias para meu shapefile#

setwd("C:/Users/daviv/OneDrive/Área de Trabalho/Modeling/genus_final") latlon

<- genus_finalLM

latlon = cbind(genus_finalgbif$sp,genus_finalgbif$x,genus_finalgbif$y)

colnames(latlon) <- c("genus","lon","lat")

species_project1=latlon

species_project1=as.data.frame(species_project1)

species_project1=na.omit(species_project1)

class(species_project1)

species_project1$lon <- as.numeric(species_project1$lon)

species_project1$lat <- as.numeric(species_project1$lat)

coordinates(species_project1)=~lon+lat

class(wrld_simpl)

projection(wrld_simpl)="+proj=longlat +datum=WGS84"

plot(wrld_simpl,col="gray 70")
```

```
projection(species_project1)="+proj=longlat +datum=WGS84"
class(species_project1)
area= shapefile("area.shp")
plot(AREA,col="gray 70")
projection(AREA)="+proj=longlat +datum=WGS84"
points(species_project1,col='green',pch=20,cex=0.75)
points(species_project1,col='black',cex=0.75)
over_points1=over(species_project1,as(AREA,"SpatialPolygons"))
species_project_over1=species_project1[!is.na(over_points1),]
plot(AREA,col="gray 90")
points(species_project_over1,col='red',pch=20,cex=0.75)
points(species_project_over1,col='black',cex=0.75)
write.csv(species_project_over1,"C:/Users/daviv/OneDrive/Área de Trabalho/Modeling/genus_SE/genus_SE_LMCR.csv", row.names = FALSE)


#Só corrigi um problema de uma das ocorrências#
genus1 <-
cbind(genus_SE_LGM$genus,genus_SE_LGM$lon,genus_SE_LGM$lat)
genus1 <- genus_SE_LM
colnames(genus1) <- c("genus","lon","lat")
genus1 <- as.data.frame(genus1)
rownames(genus1)[rownames(genus1) == "Mycetomoellerius/Paratrachymyrmex"] <- "Mycetomoellerius"
rownames(genus1) <- gsub("Mycetomoellerius/Paratrachymyrmex", "Mycetomoellerius", rownames(genus1))
genus1 = genus1[order(genus1$genus), ]
genus1 <- df1


setwd("C:/Users/daviv/OneDrive/Área de Trabalho/Modeling/Genus_BR_Cleaned")
```

```
####LIMPEZA DOS DADOS####

genus1 <-
cbind(GBIF_BRASIL$genus,GBIF_BRASIL$decimalLongitude,GBIF_BRASIL$de
cim alLatitude)

colnames(genus1) <- c("genus","lon","lat")

genus1 = as.data.frame(genus1)

genus1 <- na.omit(genus1)

a = unique(genus1$genus)


teste = function(latlon){
 latlon = subset(genus1, genus==i)


 ####plot a map to mark our points####
 library(maptools)
 data(wrld_simpl)
 plot(wrld_simpl, axes=TRUE, col="light yellow")


 #plot the points inside the map points(latlon, col='orange', pch=20, cex=0.75)
 points(latlon[,2:3], col='orange', pch=20, cex=0.75)
 points(latlon[,2:3], col='red', cex=0.75)


 ####cleaning dupplicated points####
 dupl1 <- duplicated(latlon,incomparables = FALSE)
 head(dupl1)
 dupl1=cbind(latlon,dupl1)
 df_dupl1 <- as.data.frame(dupl1)
 attach(df_dupl1)
 species_sem_duplicados1=dupl1[which(df_dupl1$dupl1=='FALSE'),]
 species_sem_duplicados1=species_sem_duplicados1[,1:3]
```

```
species_sem_duplicados1 = as.data.frame(species_sem_duplicados1)
attach(species_sem_duplicados1)

####defining geografical conditions####
library(sp)
species_project1=species_sem_duplicados1
species_project1=as.data.frame(species_project1)
species_project1=na.omit(species_project1)
species_project1$lat <- as.numeric(species_project1$lat)
species_project1$lon <- as.numeric(species_project1$lon)
class(species_project1)
coordinates(species_project1)=~lon+lat
class(wrld_simpl)
projection(species_project1)="+proj=longlat +datum=WGS84"
class(species_project1)
plot(wrld_simpl,col="gray 70")
####use 6 to put the random forest####
if(nrow(species_project1) > 6){
r1=raster(species_project1)
  res(r1)= 0.00833333 #ca. 1 km
  r1=extend(r1,extent(r1)+0.082)
  selecionados1=gridSample(species_project1,r1,n=1)
  selecionados1
  class(selecionados1)
  selecionados1=as.data.frame(selecionados1)
  points(selecionados1,col='red',pch=20,cex=0.75)
  points(selecionados1,col='black',cex=0.75)
  df = (i)
```

```
    selecionados1 = cbind(df,selecionados1)
    colnames(selecionados1) = c("genus","lon","lat")
    write.csv(selecionados1,paste(i,".csv",sep=""), row.names = FALSE)
  } else {
    selecionados1=as.data.frame(species_project1)
    write.csv(selecionados1,paste(i,".csv",sep=""), row.names = FALSE)
  }
}


for(i in a){ teste(latlon)
}

g_names = list.files(all.files = T, pattern = ".csv", full.names = F, recursive = TRUE)
xlxs <- lapply(g_names, read_csv)
merged = bind_rows(xlxs)
write.csv(merged,"genus_finalgbif.csv", row.names = FALSE)


####library packages to SSDM####

pkg <-
c("rJava","CoordinateCleaner","usdm","devtools","rgbif","VSURF","tmap","dismo","raster","rgdal","randomForest",
"maptools","sp","rgeos","ncdf4","maps","foreach","doParallel","rnaturalearthdata","SSDM","magrittr","tidyverse","readxl","dplyr","rgbif","tiff","sf","gdal","geos")
lapply(pkg, require, character.only=T)
```

```
####just the SSDM process####

env1 <- load_var("/home/davivilaca/Modeling/variables 2081-2100/otimistic/SN/5
min/IPSL/used/",files = NULL,format = ".tif", categorical = 'NULL', verbose = FALSE)

env <- load_var("/home/davivilaca/1970cr/VIF/",files = NULL, categorical = 'NULL',
verbose = FALSE)

env

occorrences <- load_occ("/home/davivilaca/Modeling/genus_finalfinal",env,file =
"genus_finalLM.csv",Xcol="lon",Ycol="lat",Spcol="genus",sep=",",verbose=TRUE,G
UI
=FALSE,GeoRes = TRUE)

occorrences <- load_occ("/home/davivilaca/Modeling/genus_finalfinal",env,file =
"genus_finalgbif.csv",Xcol="lon",Ycol="lat",Spcol="genus",sep=",",verbose=TRUE,G
U I=FALSE,GeoRes = TRUE)

#apply a method of sub-sample to deal with samPle bias IF YOU

NEED# set.seed(42)

subset_occurrences <- occorrences[sample(nrow(occorrences),
nrow(occorrences)*0.5), ]

write.csv(subset_occurrences, file =
"/home/davivilaca/Modeling/subsampletest0.5LM.csv")

occorrences <- load_occ("/home/davivilaca/Modeling",env,file =
"subsampletest0.5LM.csv",Xcol="lon",Ycol="lat",Spcol="genus",sep=",",verbose=
TR UE,GUI=FALSE,GeoRes = TRUE)

obj  <-  stack_modelling(c("MAXENT","SVM"),
```

```
occorrences,
env,
rep = 20,
Xcol = 'lon',
Ycol = 'lat', Spcol = 'genus',
Pcol = NULL,
name = "LM MODEL MAXENT",
method =
"PRR.pSSDM",
verbose =TRUE ,
parallel = TRUE,
uncertainty = TRUE,
cores=10,path = "/home/davivilaca",
save=TRUE,
cv = "k-fold", metric
= "TSS", cv.param
= c(5,5),
axes.metric = "AUC",
emsemble.metric = "Kappa",
emsemble.thresh = 0.75,
endemism = c("CWEI","Binary"))


obj1 <- stack_modelling(c("GAM","GLM","RF"),
occorrences,
env,
rep = 10,
Xcol = 'lon',
Ycol = 'lat', Spcol = 'genus',
```

```
Pcol = NULL,
name = "LM PSEUDOABSENCES",
method =
"PRR.pSSDM",
verbose =TRUE ,
parallel = TRUE,
uncertainty = TRUE,
cores=10,path = "/home/davivilaca",
save=TRUE,
cv = "k-fold",
metric = "TSS",
cv.param = c(5,5),
axes.metric = "AUC",
emsemble.metric = "Kappa",
emsemble.thresh = 0.75,
endemism = c("CWEI","Binary"))

plot(obj1)
plot(obj@diversity.map, main = 'Distribution of ants')
SSDM_proj_ot <- project(obj = obj1, Env = env1)
writeRaster(SSDM_proj@diversity.map, filename = "projectionot", path =
"/home/davivilaca", format = "GTiff" )

####Calculate the mean of two models####
# Load the two existing .tif models
model1 <- raster('/home/davivilaca/LM MODEL
MAXENT/Stack/Rasters/Diversity.tif')
```

```
model2 <- raster('/home/davivilaca/LM
PSEUDOABSENCES/Stack/Rasters/Diversity.tif')


# Extract data from each model

data_model1 <- extract(model1)

data_model2 <- extract(model2)


# Calculate the mean

mean_data <- (data_model1 + data_model2) / 2


# Create a new raster with the same extent, resolution, and projection as the original
models

new_raster <- raster(model1)


# Replace the values in the new raster with the mean data values(new_raster)

<- mean_data


# Write the new raster to a .tif file

writeRaster(new_raster, "mean_modelLM.tif", format = "GTiff", overwrite = TRUE)


library(terra)


# Carregar os arquivos .tif

global_richness <- rast("/home/esturdivant/DAVI/QGIS/mean (1).tif")

regional_richness <- rast("/home/esturdivant/DAVI/QGIS/mean_modelLM (2).tif")



global_richness <- resample(global_richness, regional_richness, method =
"bilinear")
```

```
# Extrair os valores dos rasters

global_values <- values(global_richness)

regional_values <- values(regional_richness)


# Remover NAs para calcular a correlação

complete_data <- na.omit(data.frame(Global = global_values, Regional =
regional_values))


# Calcular a correlação de Pearson

correlacao_pearson <- cor(complete_data$mean..1.,
complete_data$mean_modelLM..2., method = "pearson")

# Calcular a correlação de Pearson e o valor p

resultado_cor <- cor.test(complete_data$mean..1.,
complete_data$mean_modelLM..2., method = "pearson")


# Exibir o coeficiente de correlação e o valor p

coeficiente_cor <- resultado_cor$estimate  # Coeficiente de correlação

valor_p <- resultado_cor$p.value # Valor p


print(paste("Coeficiente de correlação: ", coeficiente_cor))

print(paste("Valor p: ", valor_p))

print(correlacao_pearson)

library(ggplot2)


# Criar o gráfico de dispersão
```

```
grafico_dispersao <- ggplot(complete_data, aes(x = mean..1., y =
mean_modelLM..2.)) +
  geom_point(color = "#2E86C1", alpha = 0.7, size = 2) +  # Cor e tamanho dos
pontos
  geom_smooth(method = "lm", color = "red", se = FALSE) + # Linha de regressão
linear
  labs(title = "Correlação de Riqueza entre Repositórios",
       x = "Repositório JUNTOS",
       y = "Repositório REGIONAL")
  + theme_minimal() +
  theme(
    panel.grid.major = element_blank(),  # Remover grids principais
    panel.grid.minor = element_blank(), # Remover grids menores
    plot.title = element_text(hjust = 0.5, size = 16, face = "bold"), # Centralizar título
    axis.title = element_text(size = 14),
    axis.text = element_text(size = 12)
  ) +
  # Adicionar anotação de correlação de Pearson
  annotate("text", x = Inf, y = Inf, label = "Pearson corr.: 0.99",
           hjust = 1.1, vjust = 2, size = 5, color = "black")

# Exibir o gráfico
print(grafico_dispersao)
```
